# PITX2 induction leads to impaired cardiomyocyte function in arrhythmogenic cardiomyopathy

**DOI:** 10.1016/j.stemcr.2023.01.015

**Published:** 2023-03-02

**Authors:** Sebastiaan J. van Kampen, Su Ji Han, Willem B. van Ham, Eirini Kyriakopoulou, Elizabeth W. Stouthart, Birgit Goversen, Jantine Monshouwer-Kloots, Ilaria Perini, Hesther de Ruiter, Petra van der Kraak, Aryan Vink, Linda W. van Laake, Judith A. Groeneweg, Teun P. de Boer, Hoyee Tsui, Cornelis J. Boogerd, Toon A.B. van Veen, Eva van Rooij

**Affiliations:** 1 Hubrecht Institute, Royal Netherlands Academy of Arts and Sciences (KNAW) and University Medical Center Utrecht, Uppsalalaan 8, 3584 CT Utrecht, the Netherlands; 2 Department of Medical Physiology, University Medical Center Utrecht, Utrecht, the Netherlands; 3 Department of Physiology, Amsterdam University Medical Centers, Amsterdam Cardiovascular Sciences, Location VU Medical Center, the Netherlands; 4 Department of Pathology, University Medical Centre Utrecht, Utrecht, the Netherlands; 5 Department of Cardiology, University Medical Center Utrecht, Utrecht, the Netherlands

**Keywords:** arrhythmogenic cardiomyopathy, induced pluripotent stem cells, PITX2, desmoplakin, desmosome, cardiomyocyte, function

## Abstract

Arrhythmogenic cardiomyopathy (ACM) is an inherited progressive disease characterized by electrophysiological and structural remodeling of the ventricles. However, the disease-causing molecular pathways, as a consequence of desmosomal mutations, are poorly understood. Here, we identified a novel missense mutation within desmoplakin in a patient clinically diagnosed with ACM. Using CRISPR-Cas9, we corrected this mutation in patient-derived human induced pluripotent stem cells (hiPSCs) and generated an independent knockin hiPSC line carrying the same mutation. Mutant cardiomyocytes displayed a decline in connexin 43, NaV1.5, and desmosomal proteins, which was accompanied by a prolonged action potential duration. Interestingly, paired-like homeodomain 2 (PITX2), a transcription factor that acts a repressor of connexin 43, NaV1.5, and desmoplakin, was induced in mutant cardiomyocytes. We validated these results in control cardiomyocytes in which *PITX2* was either depleted or overexpressed. Importantly, knockdown of *PITX2* in patient-derived cardiomyocytes is sufficient to restore the levels of desmoplakin, connexin 43, and NaV1.5.

## Highlights

• The p.Tyr1188His mutation in desmoplakin drives arrhythmogenic cardiomyopathy
• Mutant cardiomyocytes show a global decline in intercalated disc proteins
• Mutant cardiomyocytes show induced levels of the transcriptional repressor PITX2
• Suppression of *PITX2* restores expression of desmoplakin, connexin 43, and NaV1.5

## Graphical abstract

**Figure:**
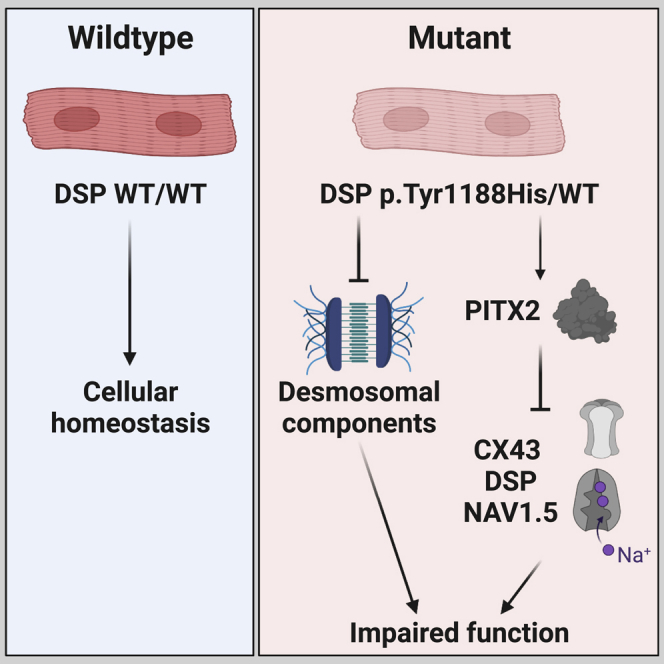

## Teaser

Here, van Kampen and colleagues investigated the consequences of a novel missense mutation in desmoplakin, identified in a patient diagnosed with arrhythmogenic cardiomyopathy. The authors observed reduced levels of intercalated disc proteins and a prolongation of the action potential duration in mutant cardiomyocytes. Furthermore, they identified aberrant PITX2 signaling as potential disease characteristic, as knockdown could restore the molecular phenotype.

## Introduction

Desmosomes are essential multiprotein complexes localized at the intercalated disc, physically connecting the intermediate filament (IF) networks of neighboring cardiomyocytes (Vermij et al., 2017). Besides providing resilience against mechanical forces during cardiac contraction, the desmosomes also play a crucial role in signal transduction (Chen et al., 2014; Garcia-Gras et al., 2006). The desmosomes encompass five essential components, the cadherins desmoglein (DSG) and desmocollin (DSC), the armadillo proteins plakoglobin (JUP) and plakophillin-2 (PKP2), and desmoplakin (DSP). The classical view of desmosomes is that they operate as a single functioning structure. However, recent studies demonstrated a close connection between desmosomes, adherens junctions, gap junctions, and ion channels, together forming the area composita (Jansen et al., 2012; Veeraraghavan and Gourdie, 2016; Vermij et al., 2017).

Mutations in desmosomal genes are frequently identified as an underlying cause of cardiovascular disease, notably in patients diagnosed with arrhythmogenic cardiomyopathy (Basso et al., 2018; Marcus et al., 1982). This progressive condition is characterized by loss of cardiomyocytes, gradual replacement of the myocardium by fibro-fatty deposits, and life-threatening ventricular arrhythmias (Hoorntje et al., 2017). Patients bearing mutations in *DSP* frequently present a complex clinical phenotype characterized by a wide range of cardiac abnormalities (Gao et al., 2020; Wang et al., 2022). To date, a number of pivotal studies focused on the role of DSP in arrhythmogenic cardiomyopathy (ACM) pathogenesis. In one study, cardiomyocyte-specific overexpression of the *DSP p.Arg2834His* mutation resulted in increased levels of apoptotic cardiomyocytes, lipid accumulation, cardiac fibrosis, and dysfunction (Yang et al., 2006). Interestingly, and in concordance with the clinical phenotype, these mice displayed accelerated ACM pathogenesis when exposed to endurance exercise, which could be linked to aberrant Wnt/β-catenin signaling (Martherus et al., 2016). Similarly, cardiomyocyte-restricted deletion of one *Dsp* allele in mice led to accumulation of JUP in the nucleus, where it competes with β-catenin, subsequently affecting Wnt-signaling pathways (Garcia-Gras et al., 2006). In addition to an impaired Wnt/β-catenin signaling axis, diminished DSP levels lead to cardiac conductance abnormalities due to mislocalization and reduced levels of connexin 43 (CX43) and sodium voltage-gated channel, alpha subunit 5 (NaV1.5) at the intercalated disc, indicating that DSP plays a role in maintaining CX43 and NaV1.5 stability in cardiomyocytes (Gomes et al., 2012; Gusev et al., 2020; Lyon et al., 2014; Zhang et al., 2013). Even though these studies significantly increased our understanding of DSP-driven ACM, current treatments focus on relieving the symptoms rather than curing the disease due to a lack of therapeutic targets.

To contribute to a better understanding of the pathomolecular mechanisms at play, we used human induced pluripotent stem cell (hiPSC)-derived cardiomyocytes to extensively study a novel heterozygous missense mutation in *DSP* (gene: c.3562T>C; protein: p.Tyr1188His), identified in a patient with the clinical diagnosis of ACM. Compared with wild-type cardiomyocytes, we observed a prolonged action potential duration (APD) and reduced expression of desmosomal components, which was paralleled by abnormal levels of CX43 and NAV1.5. Strikingly, the transcription factor paired-like homeodomain 2 (PITX2), which acts as a repressor of structural and ion channel-related genes, was induced in these cells. Knockdown of *PITX2* in mutant cardiomyocytes led to restoration of DSP, CX43, and NaV1.5. Together, we identified *DSP c.3562T>C* as a novel pathogenic mutation and demonstrated that aberrant levels of PITX2 in response to mutant DSP might contribute to the electrophysiological abnormalities often seen in patients with ACM.

## Results

### A novel genetic missense mutation in *DSP* alters its homodimerization properties

**Figure 1:**
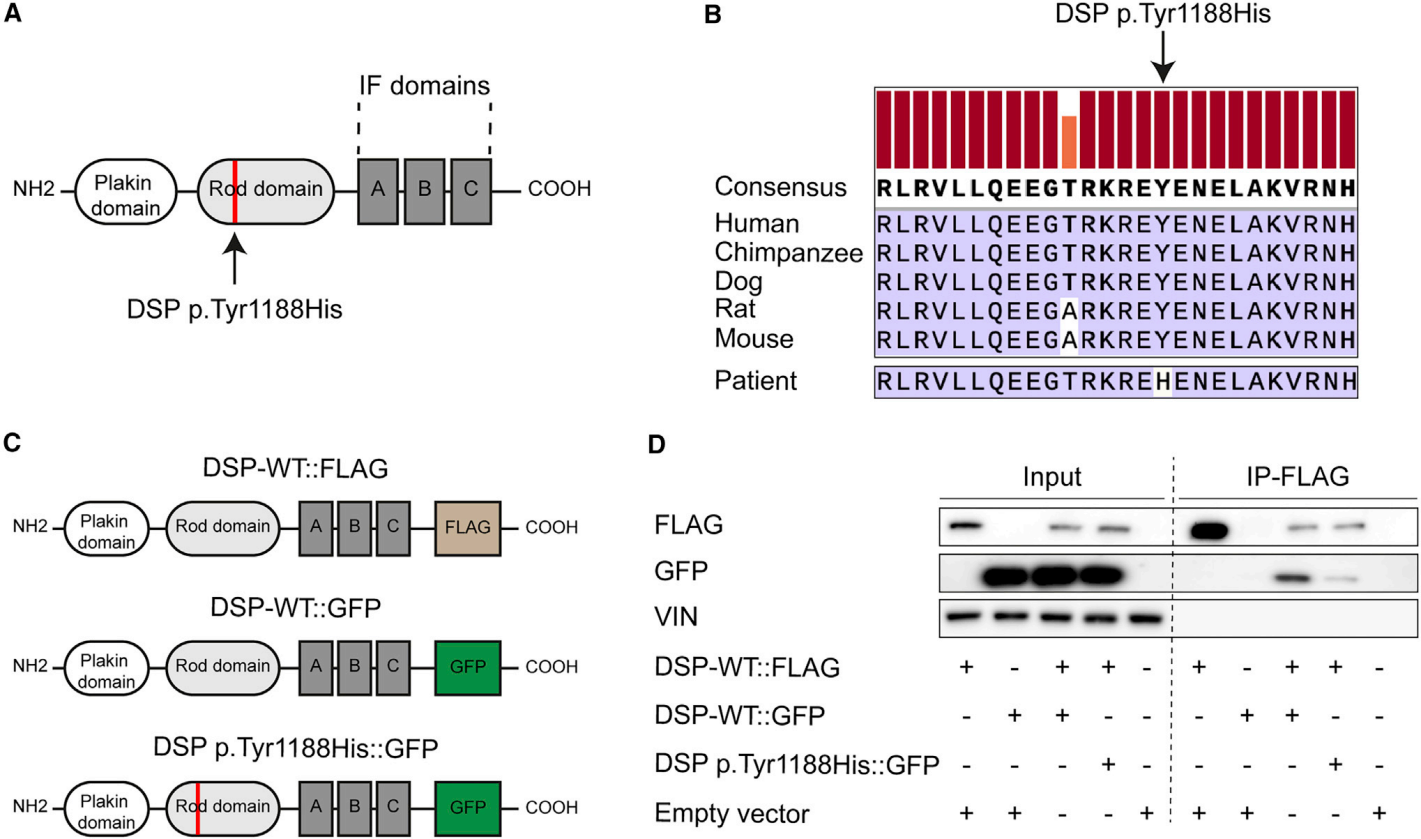
The novel missense mutation p.Tyr1188His in desmoplakin affects its homodimerization properties (A) Schematic of DSP displaying the functional domains. (B) Alignment of the protein sequence-of-interest for the indicated species. The height of the orange and red bars above each amino acid indicates the degree of conservation. (C) Overview of the constructs used to assess the binding properties between wild-type (WT) and mutant DSP molecules. (D) Representative immunoblots for FLAG, GFP, and vinculin (VIN) after co-immunoprecipitation of DSP-WT::FLAG in HEK293 cells transfected with the indicated conditions. (A–C) The novel mutation is either indicated in red or with an arrow. IF, intermediate filament; IP, immunoprecipitation.

In a patient diagnosed with ACM and experiencing monomorphic ventricular tachycardia, abnormal repolarization, and akinesia of the right ventricular apex (Table S1), we identified a novel missense mutation within the *DSP* gene (*c.3562T>C*) that translates to an amino acid substitution of a conserved tyrosine to a histidine (p.Tyr1188His; Figures 1A and 1B). No other mutations in cardiac genes were detected. As this mutation resides within the homodimerization domain (ROD domain) of DSP, we hypothesized that this would affect its homodimerization properties (Green et al., 1990; O’Keefe et al., 1989). To study the consequences of this *DSP p.Tyr1188His* mutation on homodimerization, we generated three constructs; two in which wild-type *DSP* is fused to either FLAG (DSP-WT:FLAG) or GFP (DSP-WT::GFP) and one encoding mutant *DSP* fused to GFP (DSP p.Tyr1188His::GFP; Figure 1C). Next, we transfected these constructs into HEK293 cells and performed FLAG pull-down assays after 48 h. Immunoblotting against FLAG and DSP revealed that mutant DSP protein can still interact with WT DSP, albeit with a lower binding affinity when compared with DSP-WT::FLAG co-transfected with DSP-WT::GFP (Figure 1D). These data indicate that the novel missense mutation in *DSP* affects the homodimerization properties of DSP molecules.

### Heterozygous *DSP p.Tyr1188His* hiPSC-derived cardiomyocytes display reduced desmosomal protein levels and impaired function

**Figure 2:**
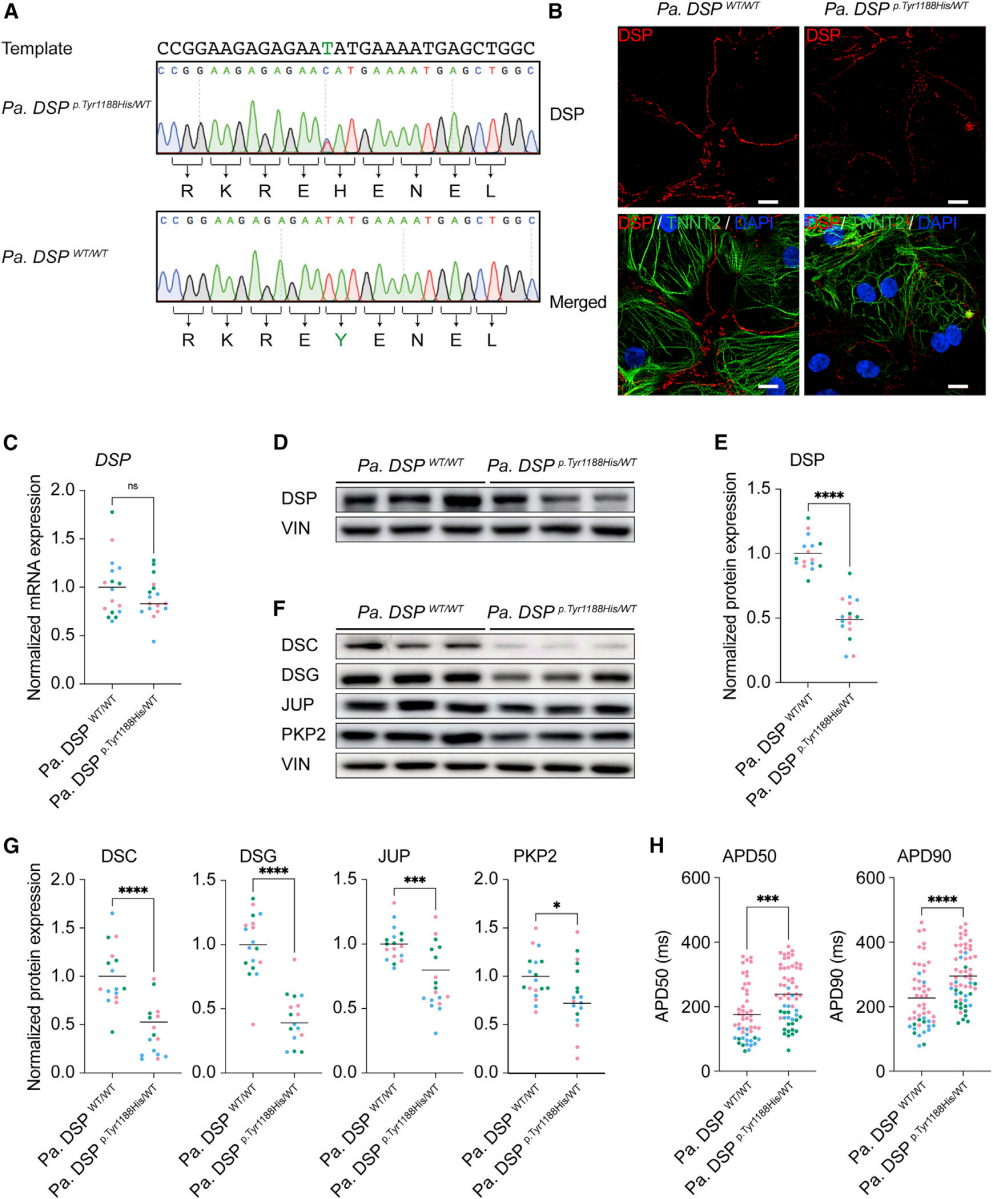
Heterozygous *Pa. DSP*^*p.Tyr1188His/WT*^ hiPSC-derived cardiomyocytes display reduced desmosomal protein levels and a prolonged action potential duration (A) Sanger sequencing traces of the patient-derived and corrected isogenic control hiPSC lines. The DNA template used for CRISPR-Cas9-targeting, correcting the *DSP*^*p.Tyr1188His*^ mutation (C > T), is shown. Intended mutation is indicated in green. (B–H) Molecular and functional analyses on 1-month-old hiPSC-derived cardiomyocytes obtained from three independent experiments. (B) Representative immunostainings for DSP. DSP in red; cardiac troponin T (TNNT2) in green; DAPI in blue. Scale bar: 10 μm. (C) Gene expression levels of *DSP* normalized to *GUS*. (D) Representative immunoblots for DSP. (E) Quantification of DSP protein levels normalized to VIN. (F) Representative immunoblots for DSC, DSG, JUP, and PKP2. (G) Quantification of the desmosomal protein levels. Values normalized to VIN. (H) Action potential duration (APD) measured at 50% and 90% of cardiomyocyte repolarization (*Pa. DSP^WT/WT^*, n = 50 cell clusters; *Pa. DSP*^*p.Tyr1188His/WT*^, n = 58 cell clusters). Data are plotted as mean. The dots in (C), (E), (G), and (H) represent technical replicates, whereas the color of each dot indicates the experimental origin (3 independent experiments; 4–34 technical replicates). Significance has been assessed by a two-tailed unpaired Student’s t test or two-tailed Mann-Whitney test when data were not normally distributed (^∗^p < 0.05, ^∗∗∗^p < 0.001, ^∗∗∗∗^p < 0.0001, ns, not significant).

To further understand the molecular consequences of this novel mutation, we reprogrammed patient skin fibroblasts to obtain hiPSCs bearing the heterozygous *DSP c.3562T>C* mutation (Table S2). Next, we corrected the mutant allele utilizing CRISPR-Cas9 in combination with a single-stranded DNA template, yielding an isogenic control line (Figures 2A and S1A). Hereinafter, we refer to these lines as *Pa. DSP^WT/WT^* and *Pa. DSP*^*p.Tyr1188His/WT*^*.* An additional synonymous mutation (blocking mutation) was included in the template to prevent recutting by Cas9 (Figure S1B). No genomic changes were observed for the top three predicted off-targets; ferredoxin 2 (*FDX1L*), integrator complex subunit 8 (*INTS8*), and zinc finger FYVE-type containing 27 (*ZFYVE27*; Figure S1C). The pluripotency markers Nanog homeobox (NANOG), POU class 5 homeobox 1 (OCT3/4), and SRY-box transcription factor 2 (SOX2) were expressed in *Pa. DSP*^*^WT/WT^*^ and *Pa. DSP*^*p.Tyr1188His/WT*^ hiPSCs (Figure S1D), and no changes in the karyotype were identified (Figures S1E and S1F). Directed differentiation of hiPSCs to cardiomyocytes yielded comparable percentages (90%–98%) of cardiac troponin T positive cells for both lines (Figure S2A). DSP, JUP, and PKP2 correctly localized to the cell periphery in 1-month-old mutant cardiomyocytes (Figures 2B, S2B, and S2C). However, molecular analyses revealed a significant reduction in DSP protein levels in mutant cells, whereas the mRNA levels were unaffected (Figures 2C–2E and S2D). Immunoblot analysis for desmosomal components DSC, DSG, JUP, and PKP2 showed a significant decline for all proteins in *Pa. DSP*^*p.Tyr1188His/WT*^ hiPSC-derived cardiomyocytes compared with the isogenic control (Figures 2F and 2G). Since the patient displayed abnormalities in the cardiac conduction system including repolarization irregularities and arrhythmias, we performed electrophysiology assays on mutant hiPSC-derived cardiomyocytes. We observed a prolonged APD at 50% and 90% of repolarization in mutant cardiomyocytes compared with control (Figure 2H). Together, cardiomyocytes bearing the novel *DSP*^*p.Tyr1188His*^ mutation show reduced desmosomal protein levels and a prolonged APD.

### Wnt-signaling- and ion-handling-related processes are affected in *Pa. DSP*^*p.Tyr1188His/WT*^ cardiomyocytes

**Figure 3:**
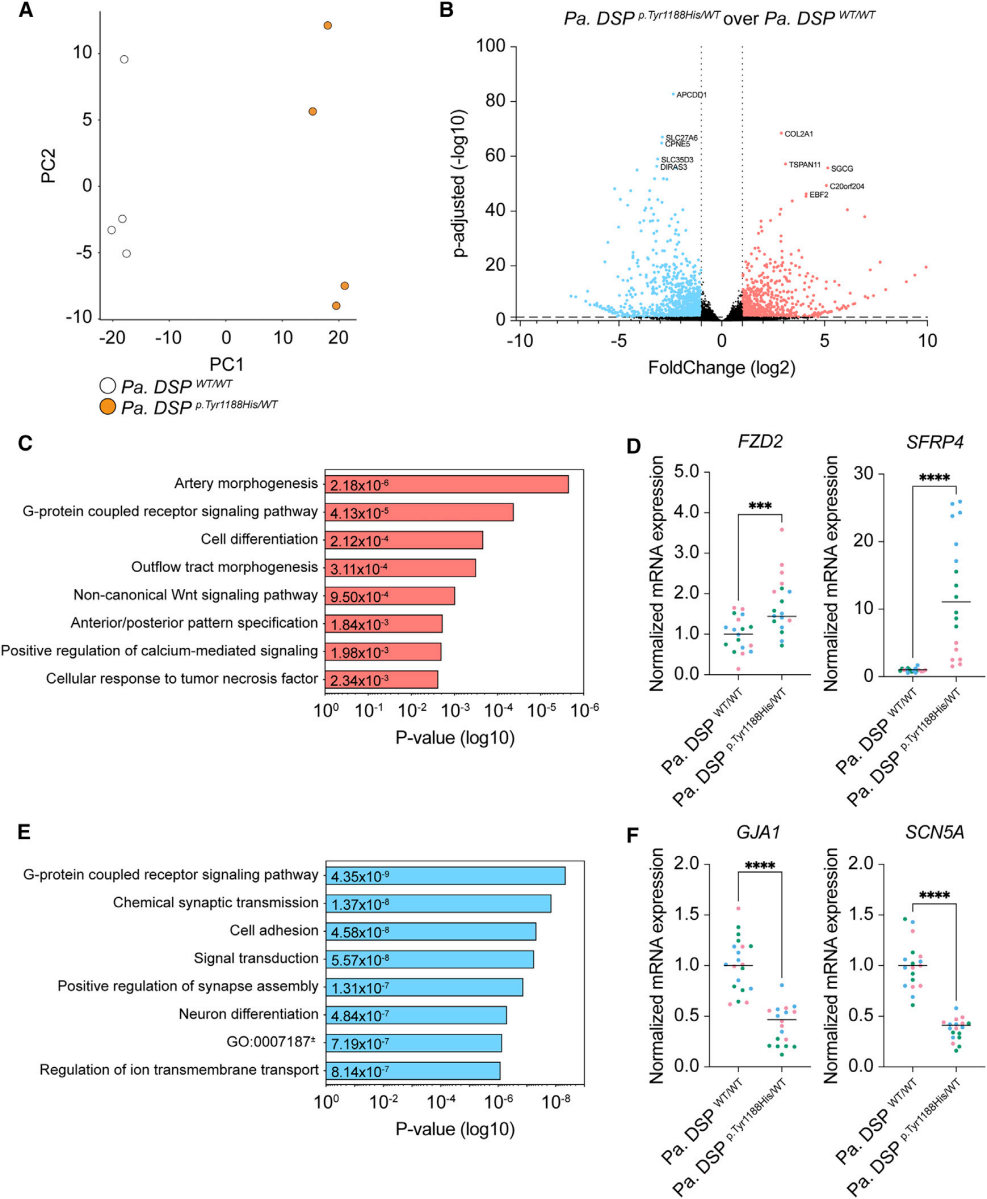
Gene networks related to Wnt signaling and ion handling are dysregulated in heterozygous *Pa. DSP*^*p.Tyr1188His/WT*^ hiPSC-derived cardiomyocytes (A–F) mRNA sequencing analyses on 1-month-old hiPSC-derived cardiomyocytes. (A) Principal-component analysis (PCA) for *Pa. DSP*^*WT/WT*^ and *Pa. DSP*^*p.Tyr1188His/WT*^ cardiomyocytes. (B) Volcano plot showing the up- and downregulated genes (fold change (log2) >1 and <−1; p-adj < 0.05) in *Pa. DSP*^*p.Tyr1188His/WT*^ and control cardiomyocytes. The top five up- and downregulated genes are indicated. (C) Gene Ontology analysis on the upregulated (fold change (log2) >1) genes. (D) Validation of *FZD2* and *SFRP4*, both belonging to the “non-canonical Wnt-signaling pathway” term. (E) Gene Ontology analysis on the downregulated (fold change (log2) <−1) genes. (F) Validation of *GJA1* and *SCN5A*, both belonging to the “regulation of ion transmembrane transport” term. mRNA sequencing was performed on four replicates obtained from one hiPSC differentiation. The dots in (D) and (F) represent technical replicates, whereas the color of each dot indicates the experimental origin (3 independent experiments; 4–8 technical replicates). Gene expression data are normalized to *GUS* and plotted as mean. Significance has been assessed by a two-tailed unpaired Student’s t test (^∗∗∗^p < 0.001, ^∗∗∗∗^p < 0.0001). GO: 0007187, G protein-coupled receptor signaling pathway coupled to cyclic nucleotide second messenger.

Next, we aimed to identify affected signaling cascades that could contribute to the observed phenotype. To this end, we performed mRNA sequencing on 1-month-old *Pa. DSP^WT/WT^* and *Pa. DSP*^*p.Tyr1188His/WT*^ cardiomyocytes. Mutant cardiomyocytes displayed a different expression profile compared with the isogenic control (Figures 3A and 3B), with 662 up- and 843 downregulated genes (log2 fold change >1 and <−1; adjusted p value [p-adj] < 0.05; Figure 3B). Gene Ontology (GO) analysis of upregulated genes identified non-canonical Wnt signaling (GO: 0035567) as enriched term (Figure 3C). Interestingly, Wnt signaling has previously been linked to ACM pathogenesis (Garcia-Gras et al., 2006). Among the gene hits for this term frizzled-2 (*FZD2*) and secreted frizzled-related protein 4 (*SFRP4*) were identified, for which the induced expression levels were validated in three independent differentiations (Figure 3D). Enriched GO terms for the downregulated genes included “cell adhesion” (GO: 0007155), “signal transduction” (GO: 0007165), and “regulation of ion transmembrane transport” (GO: 0034765; Figure 3E). Strikingly, gap junction alpha-1 protein (*GJA1*) and *SCN5A*, encoding for CX43 and NaV1.5, were among the significantly downregulated genes, which was validated in three additional batches of cardiomyocytes (Figure 3F). The aberrant expression of *GJA1* and *SCN5A* suggests that the *DSP*^*p.Tyr1188His/WT*^ mutation not only affects structural components but also cellular processes related to signal propagation and contraction.

### Knockin hiPSC-derived *DSP*^*p.Tyr1188His/WT*^ cardiomyocytes corroborate findings observed in patient-derived cardiomyocytes

**Figure 4:**
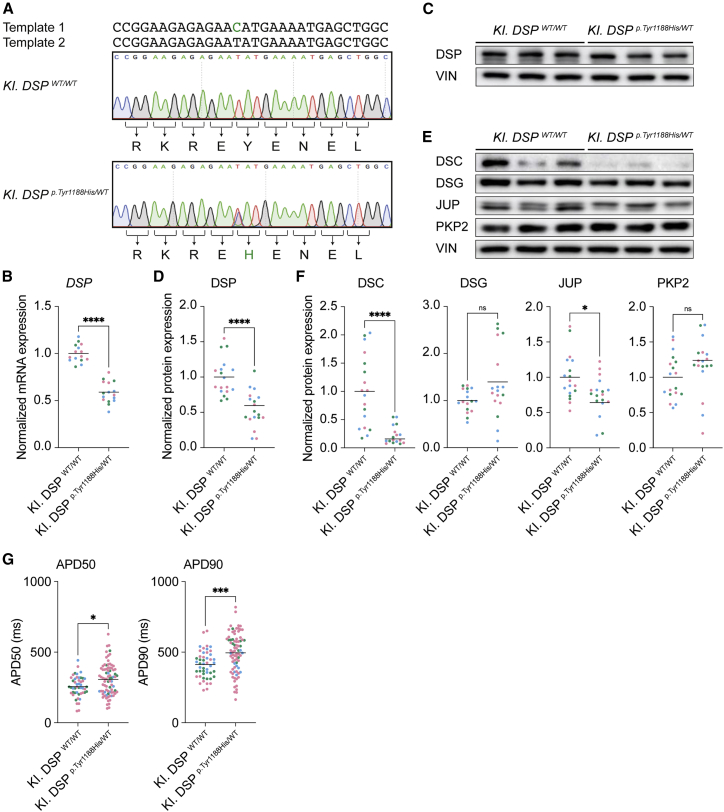
hiPSC-derived *KI. DSP*^*p.Tyr1188His/WT*^ cardiomyocytes corroborate findings observed in patient-derived cardiomyocytes (A) Sanger sequencing traces of the control and knockin hiPSC lines. The two different DNA templates used to introduce the intended mutation are depicted. Intended mutation is indicated in green. (B–G) Molecular and functional analyses on 1-month-old hiPSC-derived cardiomyocytes obtained from three independent experiments. (B) Gene expression levels of *DSP* normalized to *GUS*. (C) Representative immunoblots for DSP. (D) Quantification of DSP protein levels normalized to VIN. (E) Representative immunoblots for DSC, DSG, JUP, and PKP2. (F) Quantification of the desmosomal protein levels. Values normalized to VIN. (G) APD measured at 50% and 90% (*KI. DSP^WT/WT^*, n = 48 cell clusters; *KI. DSP*^*p.Tyr1188His/WT*^, n = 81 cell clusters) of cardiomyocyte repolarization. Data are plotted as mean. The dots in (B), (D), (F), and (G) represent technical replicates, whereas the color of each dot indicates the experimental origin (3 independent experiments; 4–60 technical replicates). Significance has been assessed by a two-tailed unpaired 2Student’s t test or two-tailed Mann-Whitney test when data were not normally distributed (^∗^p < 0.05, ^∗∗∗∗^p < 0.0001, ns, not significant).

**Figure 5:**
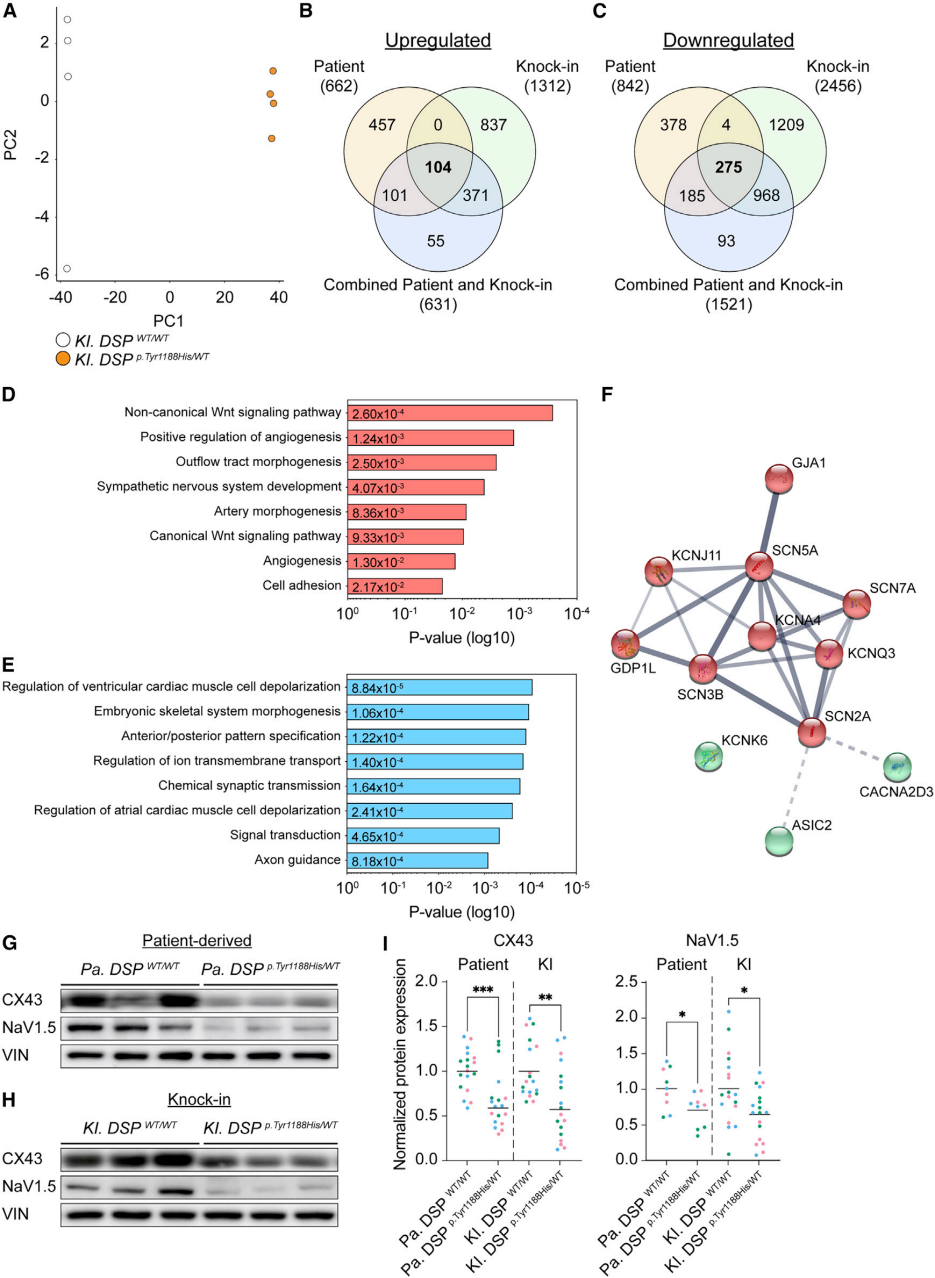
*Pa. DSP*^*p.Tyr1188His/WT*^ and *KI. DSP*^*p.Tyr1188His/WT*^ cardiomyocytes display impaired ion-handling (A) PCA reveals different gene expression profiles of *KI. DSP*^*WT/WT*^ and *KI. DSP*^*p.Tyr1188His/WT*^ cardiomyocytes. (B–F) Combined analyses of the *Pa. DSP*^*p.Tyr1188His/WT*^ (patient) and *KI. DSP*^*p.Tyr1188His/WT*^ (knockin) mRNA sequencing datasets. (B and C) Venn diagram showing the overlap of the up- (B; fold change (log2) >1) and downregulated (C; fold change (log2) <−1) genes. (D and E) Gene Ontology analyses on the up- (D) and downregulated (E) genes overlapping between the two datasets. (F) STRING: functional protein association network for the gene ontology terms “regulation of ventricular cardiac muscle cell depolarization” and “regulation of ion transmembrane transport.” Clustered based on kmeans. The thickness of each line indicates the level of confidence (data supported) and the colors to which cluster each component belongs. (G–H) Representative immunoblots for CX43 and NaV1.5 in *Pa. DSP*^*p.Tyr1188His/WT*^ (G) and *KI. DSP*^*p.Tyr1188His/WT*^ (H) and isogenic control cardiomyocytes. (I) Quantification of CX43 and NaV1.5 protein levels. Values normalized to VIN. Data are plotted as mean. The dots in (I) represent technical replicates, whereas the color of each dot indicates the experimental origin (3 independent experiments; 4–6 technical replicates). Significance has been assessed by a two-tailed unpaired Student’s t test (^∗^p < 0.05, ^∗∗^p < 0.01, ^∗∗∗^p < 0.001).

In an effort to exclude confounding effects, such as the presence of second genomic hits, we generated an independent knockin (KI) hiPSC line bearing the *DSP*^*p.Tyr1188His/WT*^ mutation. We used a healthy hiPSC line and followed the same targeting process as described above and added a second single-stranded DNA template containing only the blocking mutation (Figures S3A and 4A). Hereafter, we refer to these lines as *KI. DSP^WT/WT^* and *KI. DSP*^*p.Tyr1188His/WT*^. Amplification of potential off-target sites did not reveal any editing events (Figure S3B). Furthermore, chromosome integrity was unaffected, and the pluripotency markers NANOG, OCT3/4, and SOX2 were expressed in both lines (Figures S3C–S3E). Directed differentiation of control and *KI. DSP*^*p.Tyr1188His/WT*^ cells yielded comparable percentages (75%–82%) of cardiac troponin T-positive cells (Figure S4A). Molecular analyses revealed normal localization of DSP, JUP, and PKP2 in 1-month-old mutant cardiomyocytes (Figures S4B–S4D). Similar to *Pa. DSP*^*p.Tyr1188His/WT*^, a decline in DSP protein levels was observed in the *KI. DSP*^*p.Tyr1188His/WT*^ cardiomyocytes (Figures 4C and 4D). Interestingly, also the mRNA levels of *DSP* were reduced, which is in contrast to the patient-derived line (Figures 4B and S4E). Western blot analysis revealed a significant decline for DSC and JUP, whereas DSG and PKP2 were expressed at a similar level compared with the isogenic control (Figures 4E and 4F). Importantly, the APD at 50% and 90% of cardiomyocyte repolarization was also prolonged in *KI. DSP*^*p.Tyr1188His/WT*^ cardiomyocytes (Figure 4G). mRNA sequencing on 1-month-old *KI. DSP*^*p.Tyr1188His/WT*^ and control cardiomyocytes revealed a distinct expression profile for mutant cardiomyocytes with 1,378 and 2,499 genes up- and downregulated, respectively (log2 fold change >1 and <−1; p-adj < 0.05; Figures 5A and S5A). GO term analysis for the differentially expressed genes revealed “regulation of transcription” and “ion handling” as enriched terms (Figures S5B and S5D). Significant upregulation of the transcription factors forkhead box C2 (*FOXC2*) and lipopolysaccharide induced TNF factor (*LITAF*) was confirmed in three additional batches of differentiated cardiomyocytes (Figure S5C). In line with our observations in the patient-derived line, we noticed dysregulation of ion channels including potassium inwardly rectifying channel subfamily J member 2 (*KCNJ2*) and *SCN5A* (Figure S5E). Together, characterization of the *KI. DSP*^*p.Tyr1188His/WT*^ line corroborated the data collected from *Pa. DSP*^*p.Tyr1188His/WT*^ cardiomyocytes, demonstrating that this novel missense mutation in *DSP* is responsible for eliciting the observed molecular and functional changes.

### Combinatorial mRNA sequencing analysis of *Pa.* and *KI. DSP*^*p.Tyr1188His/WT*^ cardiomyocytes reveals impaired cardiac muscle cell depolarization

To identify the molecular changes specifically caused by the novel *DSP*^*p.Tyr1188His*^ mutation and not due to the genetic background of the lines, we performed a combinatorial analysis on the differentially expressed genes obtained from the patient (Figure 3) and KI (Figure S5) datasets. In addition to these gene lists, we also included a set of differentially expressed genes obtained from an mRNA sequencing analysis in which we combined all WT and mutant samples from both datasets (“combined patient and KI”). We identified a subset of genes that were consistently differentially expressed between the patient and KI datasets (Figures 5B and 5C). Next, we performed GO analyses on the 104 up- and 275 downregulated genes shared between all comparisons (Figures 5B–5E). The significantly upregulated genes were associated with the Wnt-signaling pathway, whereas the shared downregulated genes were enriched for “regulation of ion transmembrane transport” and “regulation of ventricular cardiac muscle cell depolarization” (Figures 5D and 5E). Next, we performed a string-db analysis using the genes within the GO terms “regulation of ion transmembrane transport” and “regulation of ventricular cardiac muscle cell depolarization,” accentuating the presence of genes fundamental for cardiomyocyte function such as *GJA1* and *SCN5A*. Reduced expression of *GJA1* and *SCN5A* was validated in three additional differentiations of 1-month-old *Pa. DSP*^*p.Tyr1188His/WT*^ and *KI. DSP*^*p.Tyr1188His/WT*^ cardiomyocytes (Figures 3F, S5E, and S5F). Immunoblot analysis confirmed that CX43 and NaV1.5 protein levels were reduced in *Pa. DSP*^*p.Tyr1188His/WT*^ and *KI. DSP*^*p.Tyr1188His/WT*^ cardiomyocytes compared with control cells (Figures 5G–5I). Together, we robustly identified impaired expression of cardiac ion-handling-related components as a consequence of the novel *DSP*^*p.Tyr1188His*^ mutation.

### PITX2 levels are increased in *DSP*^*p.Tyr1188His/WT*^ cardiomyocytes and repress expression of structural and ion-handling genes

**Figure 6:**
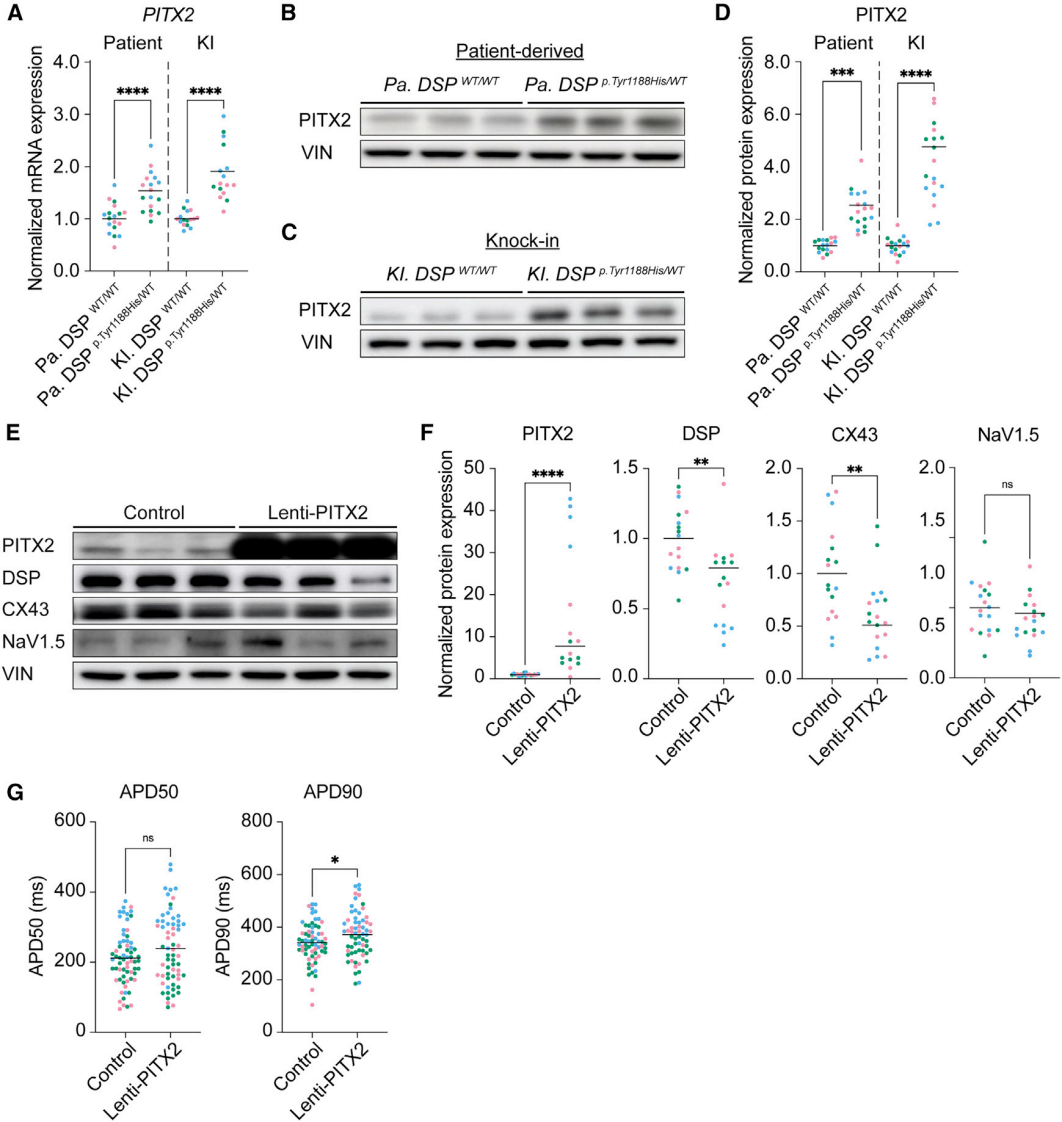
Paired-like homeodomain 2 levels are increased in mutant cardiomyocytes and repress expression of DSP, CX43, and NaV1.5 (A–D) Molecular analyses on 1-month-old *Pa. DSP*^*p.Tyr1188His/WT*^, *KI. DSP*^*p.Tyr1188His/WT*^, and isogenic control cardiomyocytes. (A) Gene expression levels for *PITX2* normalized to *GUS*. (B and C) Representative immunoblots for PITX2 in *Pa. DSP*^*p.Tyr1188His/WT*^ (B) and *KI. DSP*^*p.Tyr1188His/WT*^ (C) cardiomyocytes. (D) Quantification of PITX2 protein levels normalized to VIN. (E–G) Molecular and functional analyses on 1-month-old control hiPSC-derived cardiomyocytes treated with either empty viral particles or particles encoding for *PITX2* (lenti-PITX2). (E) Representative immunoblots for PITX2, DSP, CX43, and NaV1.5. (F) Protein levels of PITX2, DSP, CX43, and NaV1.5 normalized to VIN. (G) APD measured at 50% and 90% (control, n = 66 cell clusters; lenti-PITX2, n = 64 cell clusters) of cardiomyocyte repolarization. Data are plotted as mean. The dots in (A), (D), (F), and (G) represent technical replicates, whereas the color of each dot indicates the experimental origin (3 independent experiments; 4–29 technical replicates). Significance has been assessed by a two-tailed unpaired Student’s t test or two-tailed Mann-Whitney test when data were not normally distributed (^∗^p < 0.05, ^∗∗^p < 0.01, ^∗∗∗^p < 0.001, ^∗∗∗∗^p < 0.0001, ns, not significant). KI, knockin; Pa., patient.

The observation that many ion channels, indispensable for proper cardiomyocyte function, were dysregulated in *Pa.* and *KI. DSP*^*p.Tyr1188His/WT*^ cardiomyocytes prompted us to investigate potential upstream effectors. Martin and coworkers previously reported that the transcription factor PITX2 dictates a gene network in mouse postnatal atrial cardiomyocytes encompassing channel and calcium-handling genes as well as genes involved in stabilizing cell-cell junctions (Tao et al., 2014). The authors combined chromatin immunoprecipitation sequencing and transcriptomics on conditional *Pitx2* knockout mice, demonstrating that a loss of *Pitx2* in cardiomyocytes results in upregulation of *Dsp*, *Gja1*, and *Scn5a*, indicating that PITX2 acts as a repressor for these genes. In humans, mutations in genomic loci adjacent to or within *PITX2*, thereby affecting its expression, predisposes the heart to atrial arrhythmias (Chinchilla et al., 2011; Gudbjartsson et al., 2007; Herraiz-Martínez et al., 2021; Kaab et al., 2008; Ouwerkerk et al., 2020). Based on these findings, we hypothesized that dysregulation of PITX2 in *DSP*^*p.Tyr1188His/WT*^ cardiomyocytes might contribute to the observed reduction of DSP, CX43, and NaV1.5. mRNA sequencing analyses and subsequent validation experiments revealed an induction of *PITX2* in *Pa.* and *KI. DSP*^*p.Tyr1188His/WT*^ cardiomyocytes (Figure 6A), which was confirmed at the protein level (Figures 6B–6D). To see whether *PITX2* could evoke a similar response in an independent model, we used lentivirus to overexpress *PITX2* (lenti-PITX2) for 7 days in healthy hiPSC-derived control cardiomyocytes. *PITX2* levels were induced approximately 20-fold compared with the baseline condition (Figures S6A and S6C). The PITX2 targets *DSP*, *GJA1*, and *SCN5A* were all repressed in lenti-PITX2-treated cardiomyocytes (Figures S6B and S6C). On the protein level, we could confirm the induction of PITX2 and reduced levels of DSP and CX43 (Figures 6E and 6F). Functionally, we observed a significant prolongation of the APD at 90% of cardiomyocyte repolarization (Figure 6G). On the contrary, knockdown of *PITX2* gene expression in control cardiomyocytes induced the expression of *DSP*, *GJA1*, and *SCN5A* (Figures S6D–S6F), which was confirmed by immunoblotting for PITX2, DSP, and CX43 (Figures S6G and S6H). The APD was shortened in siPITX2-treated cardiomyocytes at 50% and 90% of repolarization compared with control (Figure S6I). These results demonstrate that PITX2 is induced in *Pa.* and *KI. DSP*^*p.Tyr1188His/WT*^ cardiomyocytes and that PITX2 represses genes important for cardiomyocyte function.

### Suppression of *PITX2* in *Pa. DSP*^*p.Tyr1188His/WT*^ cardiomyocytes restores expression of structural and ion-handling genes

**Figure 7:**
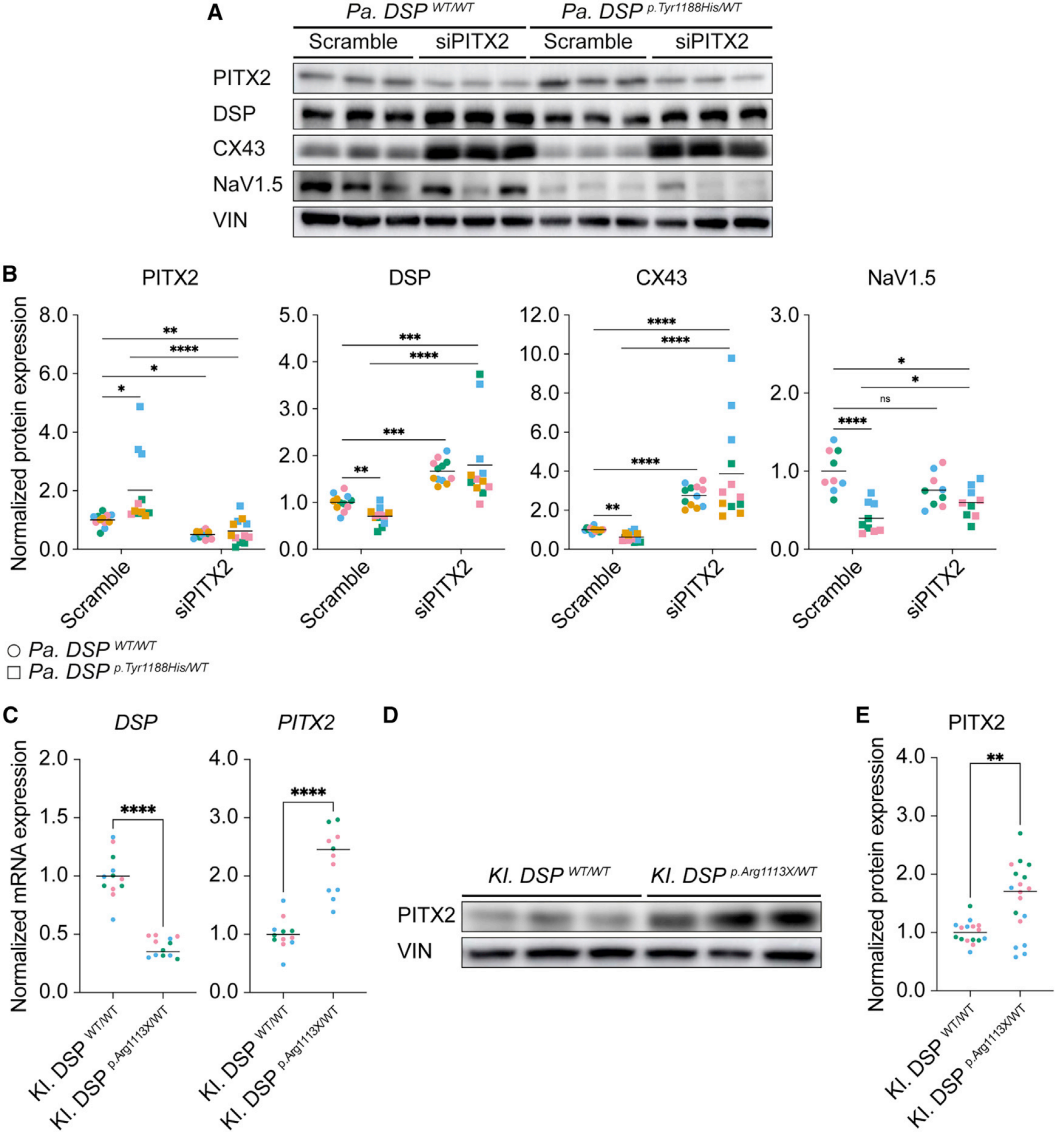
RNAi-mediated knockdown of paired-like homeodomain 2 in *Pa. DSP*^*p.Tyr1188His/WT*^ cardiomyocytes rescues the expression of structural and ion-related units (A and B) Molecular analyses on 1-month-old *Pa. DSP^WT/WT^* and *Pa. DSP*^*p.Tyr1188His/WT*^ cardiomyocytes treated with scramble siRNA or siRNA targeting *PITX2* for 72 h. Data are obtained from 3–4 independent experiments. (A) Representative immunoblots for PITX2, DSP, CX43, NaV1.5, and VIN. (B) Quantification for PITX2, DSP, CX43, and NaV1.5 normalized to VIN. (C–E) Molecular analyses on 1-month-old *KI. DSP^WT/WT^* and *KI. DSP*^*p.Arg1113X/WT*^ cardiomyocytes. Data are obtained from three independent experiments. (C) Gene expression levels for *DSP* and *PITX2* normalized to *GUS*. (D) Representative immunoblots for PITX2 and VIN. (E) Quantification of PITX2 protein levels normalized to VIN. Data are plotted as mean. The dots in (B), (C), and (E) represent technical replicates, whereas the color of each dot indicates the experimental origin (3–4 independent experiments; 3–6 technical replicates). For (B), significance has been assessed on log-transformed data using an ordinary two-way ANOVA followed by a Tukey’s multiple comparisons test (single pooled variance; alpha = 0.05). For (C) and (E), a two-tailed unpaired Student’s t test was applied (^∗^p < 0.05, ^∗∗^p < 0.01, ^∗∗∗^p < 0.001, ^∗∗∗∗^p < 0.0001, ns, not significant).

After identifying PITX2 as potential upstream effector of *DSP*, GJA1, and *SCN5A*, we speculated that knockdown of *PITX2* in *Pa. DSP*^*p.Tyr1188His/WT*^ cardiomyocytes could restore the observed phenotype. To investigate this, we treated 1-month-old mutant and isogenic control cardiomyocytes with scramble small interfering RNA (siRNA) or siRNA targeting *PITX2* for 72 h. *PITX2* levels were reduced by approximately 90% in both control and mutant cardiomyocytes, which led to a significant increase in *GJA1* expression in *Pa. DSP*^*p.Tyr1188His/WT*^ cardiomyocytes (Figures S7A and S7B). On the protein level, we observed restoration of DSP, CX43, and NaV1.5 upon silencing of *PITX2* in mutant cardiomyocytes (Figures 7A and 7B). To explore if *PITX2* induction is unique to the novel *DSP*^*p.Tyr1188His*^ mutation or whether it is a more general phenomenon for mutations in *DSP*, we assessed the levels of *PITX2* in hiPSC-derived cardiomyocytes bearing a known disease-causing mutation in *DSP* (*KI. DSP*^*p.Arg1113X/WT*^). Compared with control cells, mutant cardiomyocytes display reduced levels of *DSP* (Figures 7C and S7C). Interestingly, this reduction is accompanied by elevated levels of *PITX2*, which was confirmed at the protein level (Figures 7C–7E). Altogether, knockdown of the repressor *PITX2* in *Pa. DSP*^*p.Tyr1188His/WT*^ cardiomyocytes restores expression of genes important for cardiomyocyte function. Additionally, *KI. DSP*^*p.Arg1113X/WT*^ cardiomyocytes show induced levels of PITX2, suggesting a more general mechanism.

## Discussion

Through the use of matching patient-derived (*Pa. DSP*^*p.Tyr1188His/WT*^) and KI (*KI. DSP*^*p.Tyr1188His/WT*^) hiPSC-derived cardiomyocytes, we demonstrated that this novel *DSP* mutation evokes molecular and functional changes that can be linked to ACM pathogenesis. This implies that this mutation is causal for the observed phenotype in the patient clinically diagnosed with ACM.

Molecular analyses of *DSP*^*p.Tyr1188His/WT*^ hiPSC-derived cardiomyocytes revealed a reduction in desmosomal proteins, an observation previously reported in ventricular tissue obtained from patients with ACM (Asimaki et al., 2009). Interestingly, this pathomolecular characteristic seems to be a hallmark of ACM, as it is not evident in other forms of cardiac disease (Asimaki et al., 2009; Bueno-Beti and Asimaki, 2021). Besides reduced protein levels, we also observed lower mRNA levels for several components of the intercalated disc. This is in contrast to a recent study from our lab in which we only observed an effect on the protein level in an *in vitro* and *in vivo* KI model of an ACM-driving *PKP2* mutation, indicating that the *DSP*^*p.Tyr1188His/WT*^ mutation evokes a different molecular response (H. Tsui, S.J.K., S.J.H., V. Meraviglia, W.B.H., S. Casini, P. van der Kraak, A.V., X. Yin, M. Mayr, A. Bossu, G. Marchal, J.M.-K., J. Eding, D. Versteeg, K. Bezstarosti, J.A.G., S. Klaasen, L. van Laake, J. Demmers, G. Kops, C. Mummery, T.A.B.V., C. Remme, M. Bellin, E.R., unpublished data). In support of this, a change in the transcriptomic landscape was demonstrated through dysregulation of genes associated with ion handling, Wnt signaling, and transcriptional activity. Of note, compared with control cardiomyocytes, *KI. DSP*^*p.Tyr1188His/WT*^ cardiomyocytes showed reduced *DSP* mRNA levels, whereas *Pa. DSP*^*p.Tyr1188His/WT*^ cardiomyocytes displayed similar expression levels. Moreover, we consistently observed higher protein levels for PITX2 in the KI line compared with the patient-derived line. Since *DSP* is one of the potential downstream targets of the repressor PITX2, it is conceivable that the repressive effect on *DSP* expression is augmented in *KI. DSP*^*p.Tyr1188His/WT*^ cardiomyocytes compared with patient-derived cardiomyocytes (Tao et al., 2014). More experiments are required to pinpoint the exact underlying molecular mechanism.

We further showed that mutant *DSP*^*p.Tyr1188His/WT*^ cardiomyocytes exhibit lower levels of the gap junction protein CX43 and NaV1.5, which in turn may be causal for the observed prolongation of the action potential. These observations are in line with previous studies that demonstrated diminished CX43 and NaV1.5 levels in explanted cardiac material isolated from patients diagnosed with ACM (Asimaki et al., 2009; Bueno-Beti and Asimaki, 2021; Jansen et al., 2012). In addition, studies revealed redistribution of CX43 to the long axis of cardiomyocytes upon desmosomal protein deficiency, which in turn may act as an arrhythmogenic substrate (Bueno-Beti and Asimaki, 2021; Oxford et al., 2007; Zhang et al., 2013). The observed reduction and redistribution of proteins crucial for electrical signal propagation in cardiomyocytes may be explained at two levels. Firstly, as the desmosomes are interconnected with adherens junctions, gap junctions, and ion channels, it is conceivable that desmosomal mutations do not only affect the integrity of desmosomes but also of the linked protein complexes (Vermij et al., 2017). Specifically, proper localization of CX43 and NaV1.5 to cell-cell junctions seem to be highly dependent on a functional desmosome (Cerrone et al., 2014; Gusev et al., 2020; Jansen et al., 2012; Lyon et al., 2014; Sato et al., 2009; Zhang et al., 2013). Secondly, influential transcriptional programs such as Hippo and Wnt signaling are partly regulated by proteins residing at the intercalated discs in cardiomyocytes (Chen et al., 2014; Conti et al., 2004; Garcia-Gras et al., 2006; Guo et al., 2020). Desmosomal instability may therefore affect these signaling cascades and in turn affect the expression of structural and ion-handling-related genes. For instance, CX43 (*GJA1*) is a target of canonical Wnt signaling (Ai et al., 2000).

Here, we uncovered that aberrant expression of PITX2 in mutant *DSP*^*p.Tyr1188His/WT*^ cardiomyocytes contribute to the observed phenotype. Interestingly, mutations within *PITX2*, which physically interacts with FOXC1 and FOXC2, have previously been linked to the ocular conditions Axenfeld-Rieger syndrome and glaucoma (Acharya et al., 2011). In the heart, *PITX2* is predominantly expressed in the left atria and well known for its role in atrial fibrillation; however, it has also been detected in ventricular tissue (Chinchilla et al., 2018; Furtado et al., 2011; Ouwerkerk et al., 2020; Tao et al., 2016; Torrado et al., 2014). Moreover, it has been proposed that atrial disease can be a subentity of heart failure induced by ventricular abnormalities (Coats et al., 2021). Mikhailov and coworkers demonstrated that *PITX2* expression is reactivated in the ventricular failing myocardium of patients experiencing systolic heart failure (Torrado et al., 2014). Likewise, *PITX2* is induced after myocardial infarction in Hippo-deficient mouse ventricles, subsequently activating expression of genes associated with the electron transport chain and reactive oxygen species scavengers (Tao et al., 2016). Through modulation of *PITX2* expression levels in mutant *Pa. DSP*^*p.Tyr1188His/WT*^ cardiomyocytes, we demonstrate reactivation of genes important for ion handling and signal propagation, including *SCN5A* and *GJA1*. Previously, PITX2 has been linked to Hippo and Wnt signaling, cascades reported to be frequently dysregulated in the setting of ACM (Basu and Roy, 2013; Briata et al., 2003; Garcia-Gras et al., 2006; Tao et al., 2016). We did notice enrichment for terms related to (non)-canonical Wnt signaling in our transcriptome datasets, which might provide a link between mutations in *DSP* and *PITX2* induction. Together, these findings imply that *PITX2* may play a role in the malfunctioning ventricular myocardium. Further research is required to unravel the exact mechanisms leading to *PITX2* induction in cardiomyocytes bearing mutations in *DSP*.

In conclusion, our results reveal that the novel *DSP*^*p.Tyr1188His*^ mutation evokes a pathological response in cardiomyocytes. We observed reduced desmosomal protein levels, which were accompanied by a reduction in CX43 and NaV1.5. Functionally, mutant cardiomyocytes displayed a prolonged APD that could be linked to induced levels of the repressor PITX2. Indeed, knockdown of *PITX2* in patient-derived cardiomyocytes could alleviate the observed repressive effects. Even though the mutation-induced molecular changes are well defined in this study, the electrophysiological properties are not because of technical limitations inherent of the followed methodology. Follow-up studies should implement patch-clamp experiments to further facilitate our understanding of the electrical alterations caused by the mutation. Together, our data underscore the advantages of combining patient and KI hiPSC-derived cardiomyocytes, bearing mutations in the endogenous locus, to identify a potential novel therapeutic target for the treatment of ACM.

## Experimental procedures

### Resource availability

#### Corresponding author

For further information, please contact Eva van Rooij (e.vanrooij@hubrecht.eu).

#### Materials availability

Materials and additional details can be made available from the corresponding author upon reasonable request.

### Co-immunoprecipitation

HEK293T cells grown on a 145 cm^2^ dish until 60%–70% confluency were transfected with the indicated plasmids using polyethylenimine. After 48 h, protein was isolated using a mild lysis buffer. Magnetic beads coated with monoclonal anti-FLAG M2 were used to pull down protein complexes containing DSP-WT:FLAG molecules. Samples were analyzed on a 7% SDS-PAGE gel using the antibodies listed in Table S4.

### Cardiomyocyte cultures

The genomic integrity and pluripotency status of genetically modified hiPSCs was assessed by means of targeted sequencing, immunofluorescence, and karyo sequencing. To initiate directed differentiation toward cardiomyocytes, hiPSCs were cultured on Geltrex-coated plates in Essential 8 Medium until 80%–90% confluency, Next, medium was refreshed with RPMI-1640-Medium-GlutaMAX-Supplement-HEPES supplemented with human recombinant albumin, L-Ascorbic Acid 2-Phosphate, and CHIR99021 (cardio differentiation medium with CHIR). After 48 h, medium was refreshed with cardio differentiation medium with IWP2. After 48 and 96 h, cells were refreshed with plain cardio differentiation medium. From day 8 onward, cells were kept in RPMI-1640-Medium-GlutaMAX-Supplement-HEPES supplemented with B-27-Supplement-serum free. hiPSC-derived cardiomyocyte cultures were subsequently analyzed by fluorescence-activated cell sorting (FACS; BD Biosciences, FACSCalibur) for the percentage of cardiomyocytes (positive for cardiac Troponin T).

### Molecular assays

hiPSC-derived cardiomyocytes were seeded at a density of 100,000 cells/cm^2^ for downstream applications. We analyzed the RNA, protein, and functional properties of 1-month-old cardiomyocytes either kept under baseline conditions, transfected with control siRNA or an siRNA targeting *PITX2*, or infected with control virus or lenti-PITX2. For the functional experiments, cells were grown in clusters and treated with FluoVolt and Powerload for 15 min at 37°C. During measurements, cells were immersed in a solution containing, in mM, NaCl (130), KCl (4), CaCl2 (1.8), MgCl2 (1.2), NaHCO3 (18), HEPES (10), and glucose (10) (pH 7.4). A custom-build microscope with a 10× objective was used for the recordings. APDs were corrected for the beating rate using an adjustment of the Fredericia formula: APDcorrected = APD/(∛(60/BPM)).

### mRNA sequencing

mRNA-sequencing was performed on 1-month-old hiPSC-derived cardiomyocytes. RNA libraries were prepared with the TruSeq Stranded mRNA polyA kit (Illumina) according to the manufacturer’s protocol. Strand-specific single-end 75 bp reads were generated on an Illumina NextSeq 500 system. Reads were checked for their quality using FastQC and aligned against the human genome (assembly GRCh37) using STAR (STAR_2.4.2a). Differential expression was calculated using DESeq2 v.1.2 with pooled dispersion estimates. Differentially expressed genes were further analyzed for functional enrichment using the STRING v.11.5 database. No changes were made to the default settings. *Homo sapiens* served as background. Only the molecular function (GO-MF), biological process (GO-BP), cellular component (GO-CC), and KEGG data sources were considered for enrichment analysis.

### Human material

The study fulfilled the Dutch criteria of the code of proper use of human tissue. Written informed consent was obtained for the generation and use of the patient-derived hiPSCs.

### Statistical analysis

The number of samples (n) used in each experiment is indicated in the legend of each figure. Data are presented as mean. Statistical analyses were performed using PRISM (GraphPad Software v. 9). Outliers were identified using the ROUT method (Q = 5%) and were removed if present. For comparison of two groups, data were tested for normality using the Kolmogorov-Smirnov test with Dallal-Wilkinson-Lillie for p value method (alpha = 0.05). Significance has been assessed by a two-tailed unpaired Student’s t test or two-tailed Mann-Whitney test when data were not normally distributed. For comparisons of four groups with two variables (genotype and treatment), data were tested for homoscedasticity (Spearman’s rank correlation test; alpha = 0.05) and Gaussian distribution (Kolmogorov-Smirnov test; alpha = 0.05). In case these requirements were not met, a log transformation was applied. Data were then tested for significance using an ordinary two-way ANOVA, followed by a Tukey’s multiple comparisons test (single pooled variance; alpha = 0.05). Correlation and significance between proteins have been assessed by non-parametric Spearman correlation (two-tailed; alpha = 0.05). Differences were considered statistically significant when p <0.05. Asterisks indicate statistical significance (^∗^p < 0.05, ^∗∗^p < 0.01, ^∗∗∗^p < 0.001, ^∗∗∗∗^p < 0.0001).

## Author contributions

S.J.v.K. designed and performed all experiments related to hiPSC targeting using CRISPR-Cas9. S.J.v.K., C.J.B., and E.v.R. designed all molecular experiments related to phenotyping of the hiPSC-derived cardiomyocytes. S.J.v.K., S.J.H., E.K., E.W.S., and J.M.-K. maintained the hiPSC lines and performed molecular experiments on hiPSC-derived cardiomyocytes. S.J.v.K. and C.J.B. performed mRNA sequencing analyses. W.B.v.H., B.G., T.P.d.B., and T.A.B.v.V. designed and performed the electrophysiology experiments. S.J.v.K., I.P., and H.d.R. designed and performed the experiments regarding co-immunoprecipitation and cloning. L.W.v.L. and J.A.G. provided the human skin biopsies and clinical data. S.J.v.K. and E.v.R. planned all experiments, performed data analyses, and wrote the manuscript.

## Data Availability

The mRNA sequencing datasets have been deposited with the Gene Expression Omnibus repository under accession numbers GEO: GSE208213 and GSE208212 for the patient and KI lines, respectively.

## Supplemental information

Document S1. Supplemental experimental procedures, Figures S1–S7, and Tables S1–S5

Document S2. Article plus supplemental information
