# Supplementary material for: PITX2 induction leads to impaired cardiomyocyte function in arrhythmogenic cardiomyopathy
Source: Stem Cell Reports. 2023 Mar 2;18(3):749–64. doi: 10.1016/j.stemcr.2023.01.015 (PMC10031305; doi:10.1016/j.stemcr.2023.01.015)
Supplement: Document S1. Supplemental experimental procedures, Figures S1–S7, and Tables S1–S5 [file mmc1.pdf]

Stem Cell Reports, Volume *18*

Supplemental Information

PITX2 induction leads to impaired cardiomyocyte function in arrhythmogenic cardiomyopathy

Sebastiaan J. van Kampen, Su Ji Han, Willem B. van Ham, Eirini Kyriakopoulou, Elizabeth W. Stouthart, Birgit Goversen, Jantine Monshouwer-Kloots, Ilaria Perini, Hesther de Ruiter, Petra van der Kraak, Aryan Vink, Linda W. van Laake, Judith A. Groeneweg, Teun P. de Boer, Hoyee Tsui, Cornelis J. Boogerd, Toon A.B. van Veen, and Eva van Rooij

Supplemental Figures

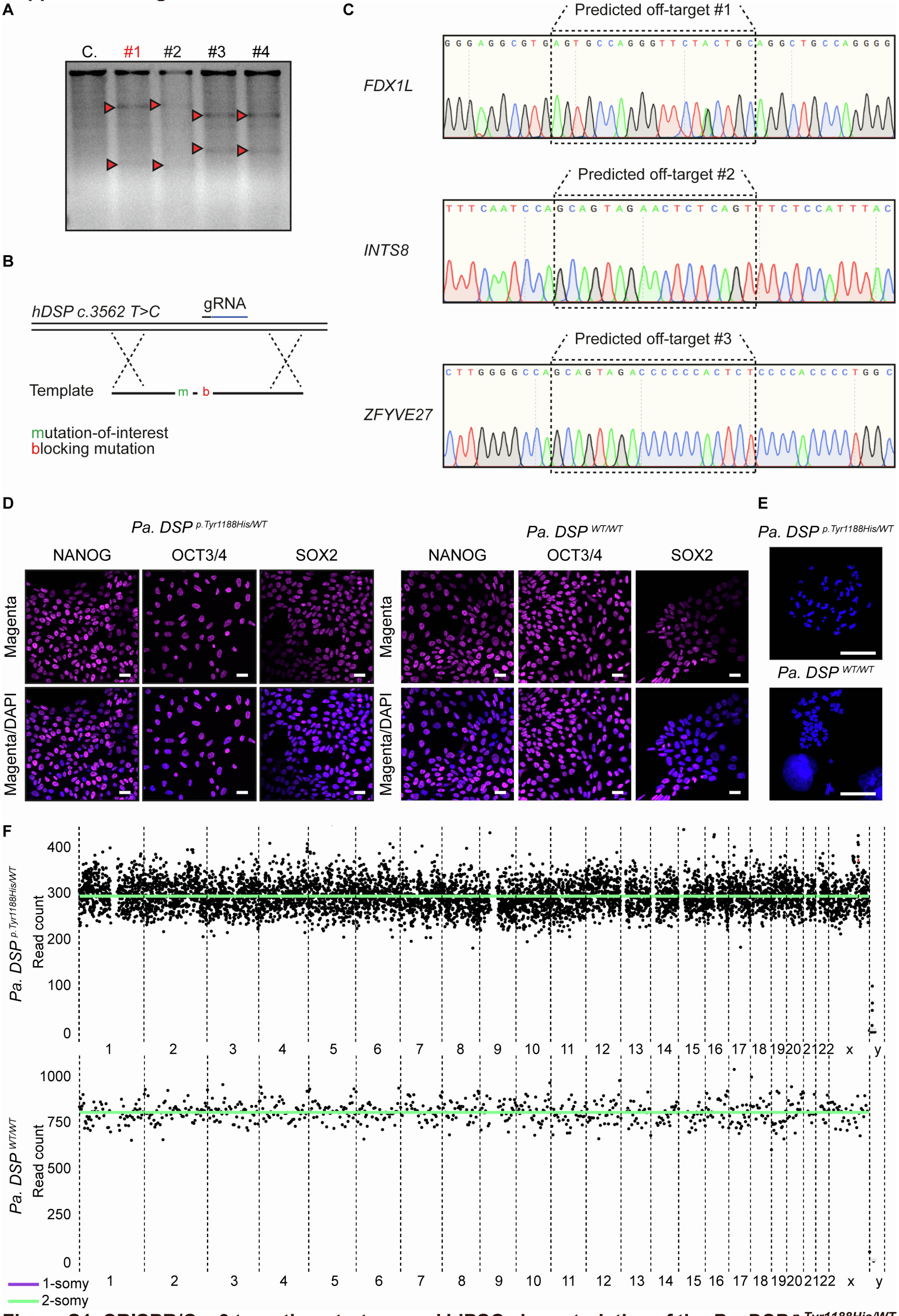

Figure S1. CRISPR/Cas9 targeting strategy and hiPSC characteristics of the *Pa. DSP* $^{p.Tyr1188His/WT}$ and isogenic control lines. Related to Figure 2.

(A) T7-endonuclease assay. gRNA #1, indicated in red, was selected for subsequent targeting. (B) Scheme depicting the CRISPR/Cas9 strategy used to correct *Pa. DSP* $^{p.Tyr1188His/WT}$ hiPSCs. Template length: 127 bp. (C) Representative Sanger sequencing traces for the top three off-target sites for gRNA #1. Dashed box indicates the potential binding site of gRNA #1 within *FDX1L*, *INTS8* and *ZFYVE27*. (D-F) Molecular analyses performed on the *Pa. DSP* $^{p.Tyr1188His/WT}$ and *Pa. DSP* $^{WT/WT}$ hiPSC lines. (D) Representative immunofluorescence images for NANOG, OCT3/4, SOX2 (all in magenta) and DAPI (blue). Scalebar: 20 μm. (E) Representative immunofluorescence images showing the correct number of chromosomes (DAPI in blue). Scalebar: 10 μm. (F) Karyo-sequencing profiles.

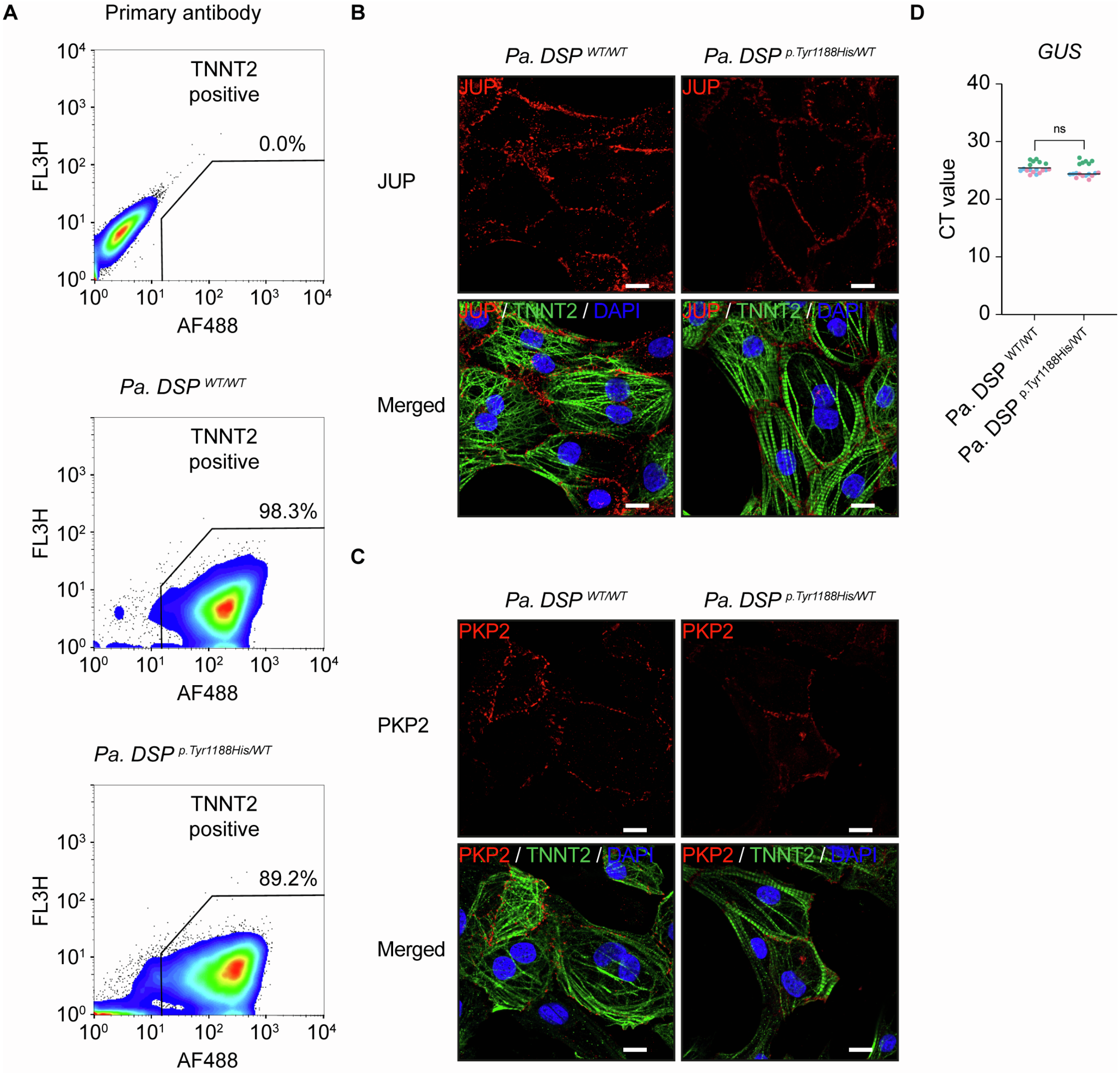

Figure S2. Differentiation and molecular characteristics of *Pa. DSP* $^{p.Tyr1188His/WT}$ and isogenic control cardiomyocytes. Related to Figure 2.

(A) Representative FACS plots showing the percentage of cardiomyocyte purity. Cells were stained with cardiac Troponin T (TNNT2, in green; AF488). (B-C) Representative immunofluorescence images for JUP (B) and PKP2 (C) in one-month-old wildtype and mutant cardiomyocytes. JUP and PKP2 in red; cardiac troponin T (TNNT2) in green; DAPI in blue. Scalebar: 10 µm. (D) Cycle threshold (CT) levels for *GUS* in one-month-old *Pa. DSP* $^{WT/WT}$ and *Pa. DSP* $^{p.Tyr1188His/WT}$ cardiomyocytes. Data plotted as mean. The dots in (D) represent technical replicates, whereas the color of each dot indicates the experimental origin (3 independent experiments; 6 technical replicates). Significance has been assessed by a two-tailed unpaired Student's t-test or two-tailed Mann-Whitney test when data were not normally distributed (ns: not significant).

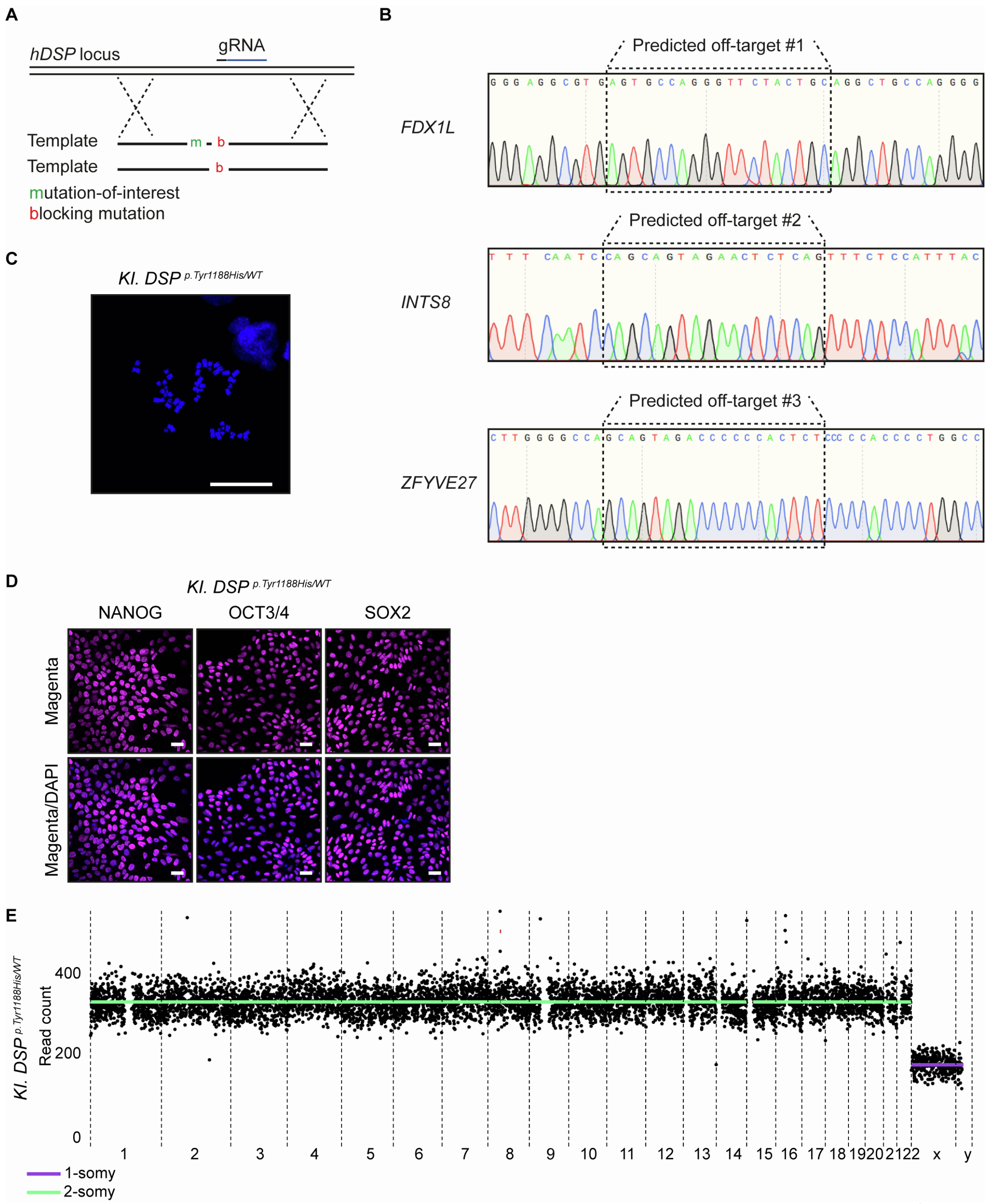

Figure S3. CRISPR/Cas9 targeting strategy and hiPSC characteristics of the *KI. DSP* $^{WT/WT}$ and *KI. DSP* $^{p.Tyr1188His/WT}$ lines. Related to Figure 4.

(A) Cartoon depicting the modified CRISPR/Cas9 strategy used to introduce the *DSP* $^{p.Tyr1188His}$ mutation in control hiPSCs. One single-stranded DNA template contains a blocking and the intended mutation, whereas the second template only contains the blocking mutation (Template length: 127 bp). (B-E) Analyses performed on the *KI. DSP* $^{p.Tyr1188His/WT}$ clone. (B) Representative Sanger sequencing traces for the top three off-target sites. Dashed box indicates the potential binding site of the gRNA within *FDX1L*, *INTS8* and *ZFYVE27*. (C) Representative immunofluorescence image showing the number of chromosomes (DAPI in blue). Scalebar: 10 μm. (D) Representative immunofluorescence images for NANOG, OCT3/4, SOX2 (all in magenta) and DAPI (blue). Scalebar: 20 μm. (E) Karyo-sequencing profile of the *KI. DSP* $^{p.Tyr1188His/WT}$ clone.

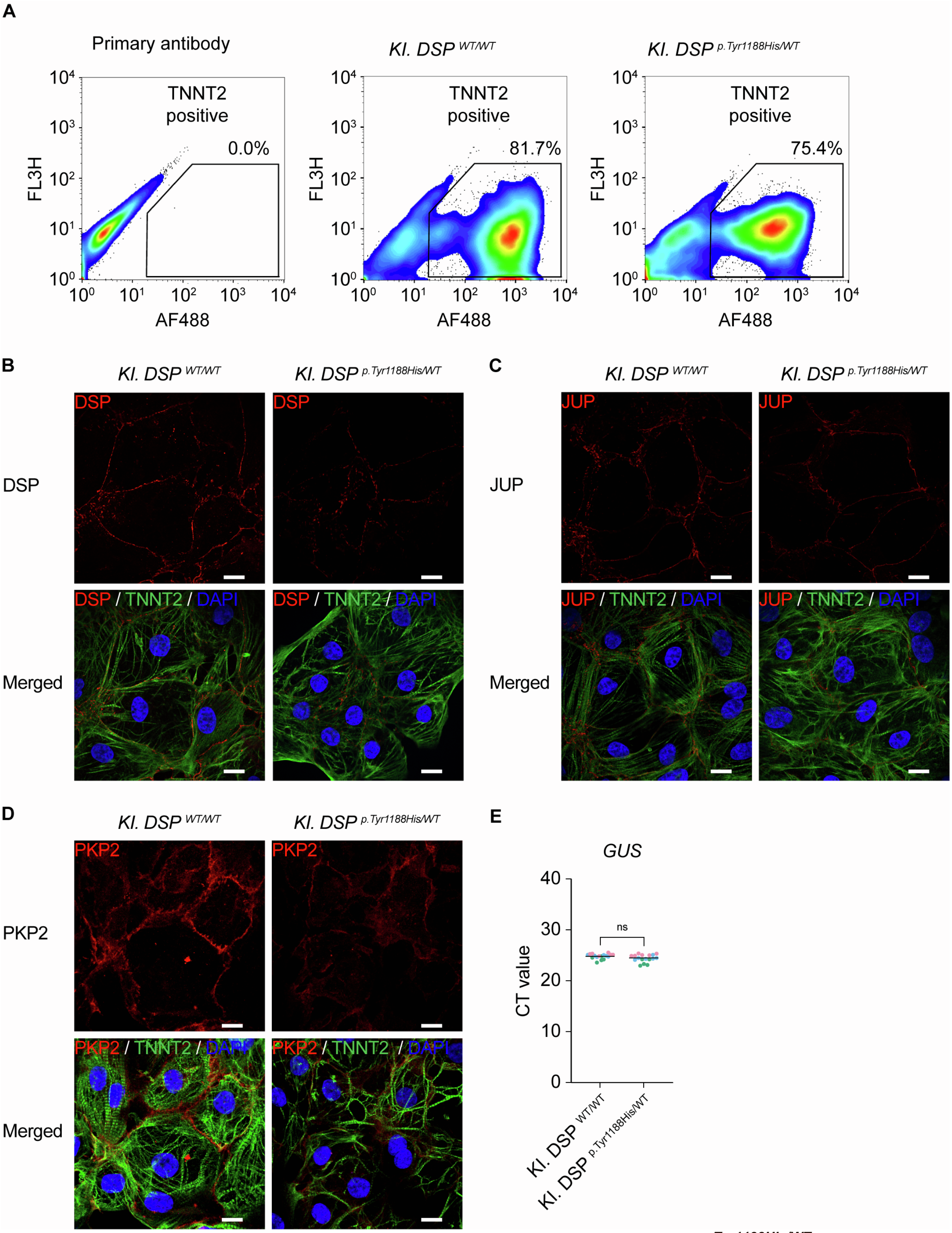

Figure S4. Differentiation and molecular characteristics of *KI. DSP* $^{p.Tyr1188His/WT}$ and isogenic control cardiomyocytes. Related to Figure 4.

(A) Representative FACS plots showing the percentage of cardiomyocytes present after differentiation. Cells were stained with cardiac Troponin T (TNNT2, in green; AF488). (B-D) Representative immunofluorescence images for DSP (B), JUP (C) and PKP2 (D) in one-month-old *KI. DSP* $^{WT/WT}$ and *KI. DSP* $^{p.Tyr1188His/WT}$ cardiomyocytes. DSP, JUP and PKP2 in red; cardiac troponin T (TNNT2) in green; DAPI in blue. Scalebar: 10 μm. (E) Cycle threshold (CT) levels for *GUS* in one-month-old wildtype and mutant hiPSC-derived cardiomyocytes. Data plotted as mean. The dots in (E) represent technical replicates, whereas the color of each dot indicates the experimental origin (3 independent experiments;

5-6 technical replicates). Significance has been assessed by a two-tailed unpaired Student's t-test or two-tailed Mann-Whitney test when data were not normally distributed (ns: not significant).

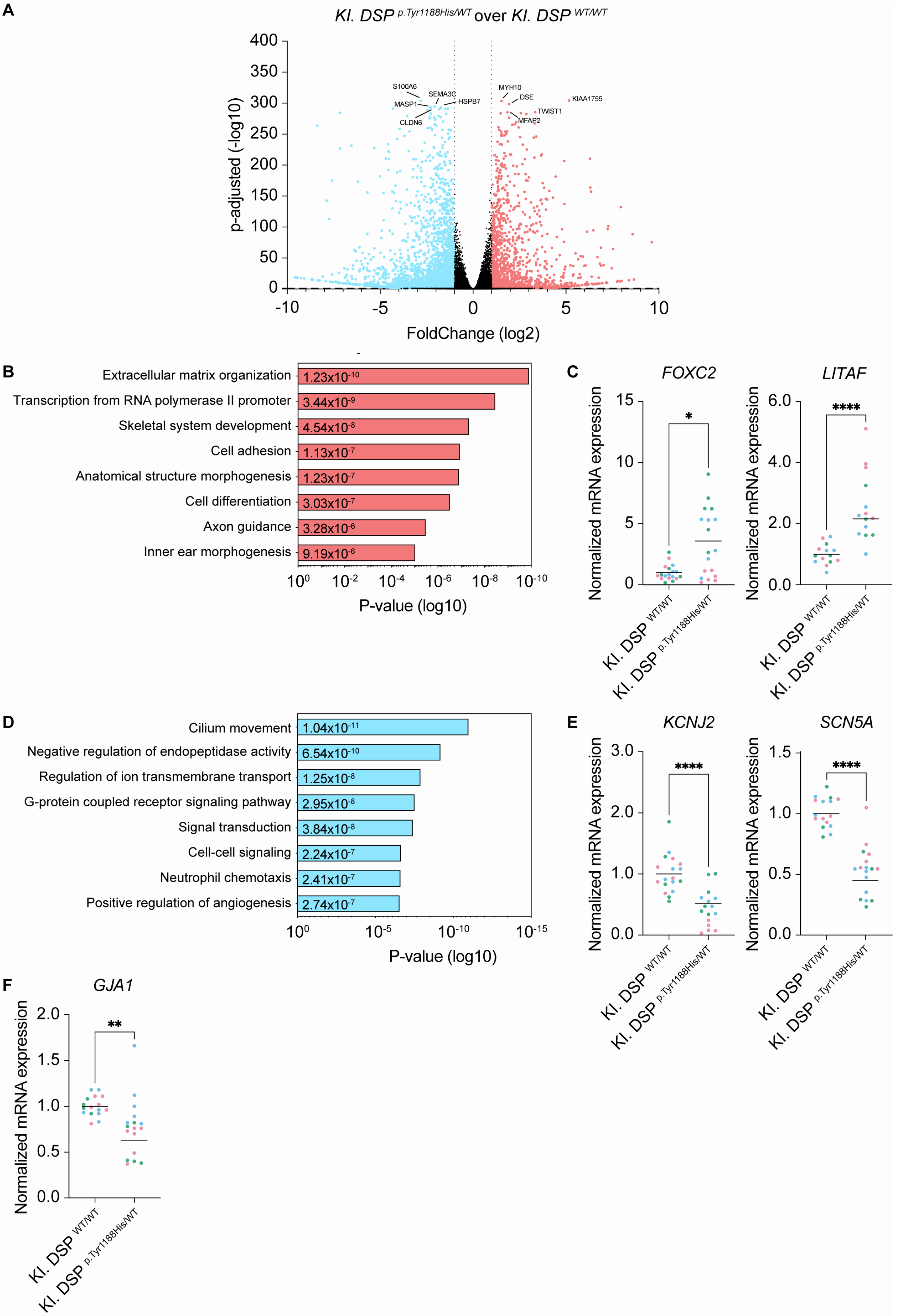

Figure S5. mRNA-sequencing analysis on *KI. DSP* $^{p.Tyr1188His/WT}$ cardiomyocytes reveals ion-handling as enriched term for repressed genes. Related to Figure 4.

(A-E) mRNA-sequencing analyses on one-month-old *KI. DSP* $^{WT/WT}$ and *KI. DSP* $^{p.Tyr1188His/WT}$ cardiomyocytes. (A) Volcano plot showing the up- and downregulated genes (fold change (log2) >1 and <-1; P-adj < 0.05) in mutant cardiomyocytes compared to the isogenic control. The top five up- and downregulated genes are indicated. (B) Gene ontology analysis on the upregulated (fold change (log2) >1) genes. (C) Validation of *FOXC2* and *LITAF*, both belonging to the "Transcription from RNA polymerase II promoter" term. (D) Gene ontology analysis on the downregulated (fold change (log2) <-1) genes. (E) Validation of *KCNJ2* and *SCN5A*, both belonging to the "Regulation of ion transmembrane transport" term. mRNA-sequencing was performed on four replicates obtained from one hiPSC differentiation. (F) Gene expression of *GJA1* in *KI. DSP* $^{WT/WT}$ and *KI. DSP* $^{p.Tyr1188His/WT}$ cardiomyocytes. Data plotted as mean. The dots in (C and E-F) represent technical replicates, whereas the color of each dot indicates the experimental origin (3 independent experiments; 4-6 technical replicates). Significance has been assessed by a two-tailed unpaired Student's t-test or two-tailed Mann-Whitney test when data were not normally distributed (* P-value < 0.05, ** P-value < 0.01, **** P-value < 0.0001).

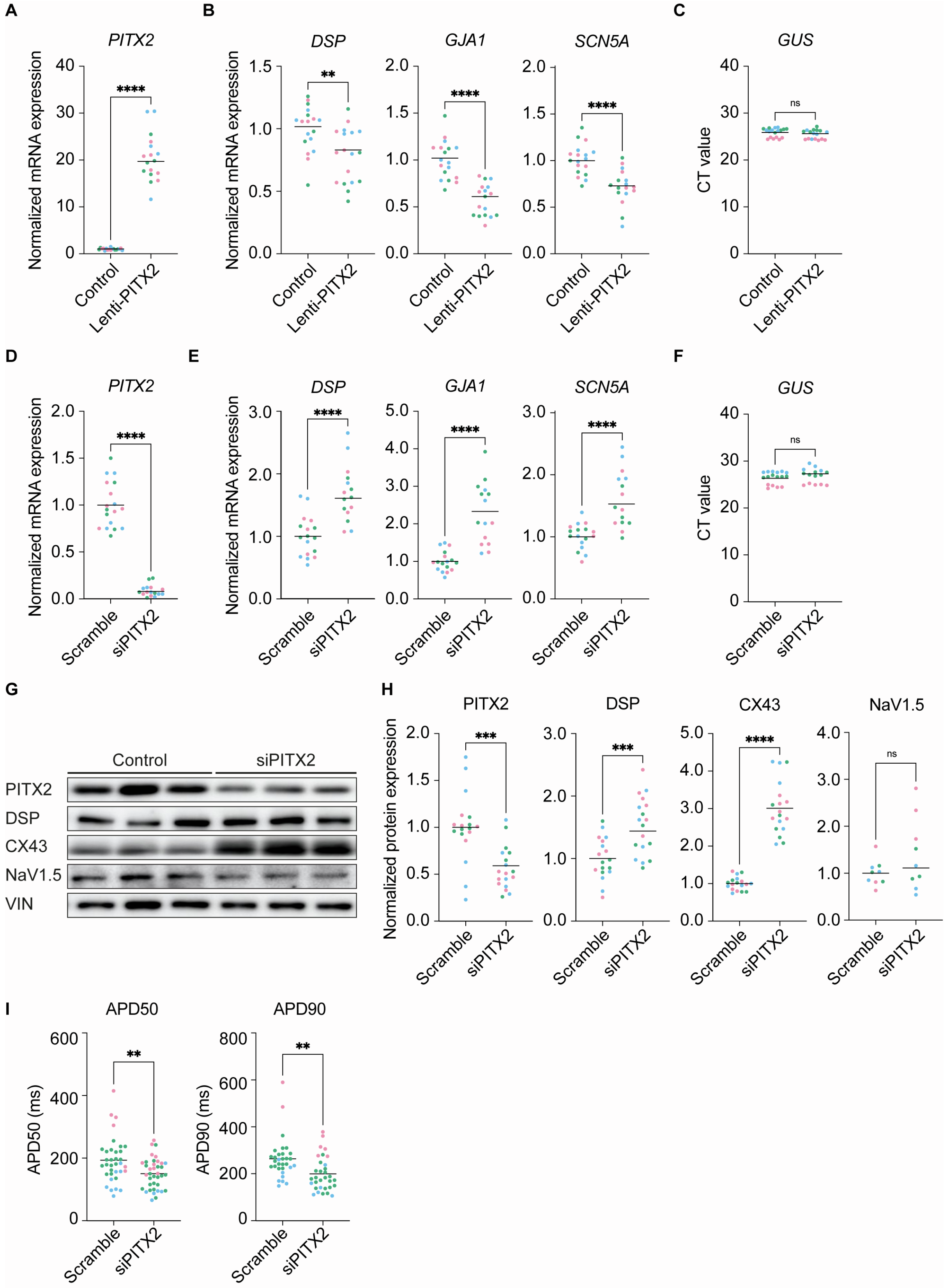

Figure S6. Overexpression and knockdown of paired-like homeodomain 2 in control cardiomyocytes represses and induces expression of cardiac ion- and structural-related genes, respectively. Related to Figure 6.

(A-C) Molecular analyses on one-month-old control hiPSC-derived cardiomyocytes treated with either control lentiviral particles or particles encoding for PITX2 (Lenti-PITX2). Data obtained from three independent experiments. (A) Gene expression levels for *PITX2*. (B) Gene expression levels for *DSP*,

GJA1 and *SCN5A*. (C) Cycle threshold (CT) levels for *GUS* in the corresponding experiments. (D-I) Molecular and functional analyses on one-month-old control hiPSC-derived cardiomyocytes treated with scramble or siRNA against *PITX2*. Data obtained from three independent experiments. (D) Gene expression levels for *PITX2*. (E) Gene expression levels for *DSP*, *GJA1* and *SCN5A* normalized to *GUS*. (F) Cycle threshold (CT) levels for *GUS* in the corresponding differentiations. (G) Representative immunoblots for PITX2, DSP, CX43 and NaV1.5. (H) Quantification of PITX2, DSP, CX43 and NaV1.5 protein levels. Values normalized to VIN. (I) Action potential duration (APD) measured at 50% and 90% of repolarization (control, n= 31 cell clusters; siPITX2, n= 32 cell clusters). Data plotted as mean. The dots in (A-F and H-I) represent technical replicates, whereas the color of each dot indicates the experimental origin (3 independent experiments; 4-20 technical replicates). Significance has been assessed by a two-tailed unpaired Student's t-test or two-tailed Mann-Whitney test when data were not normally distributed (* P-value < 0.05, ** P-value < 0.01, *** P-value < 0.001, **** P-value < 0.0001, ns: not significant).

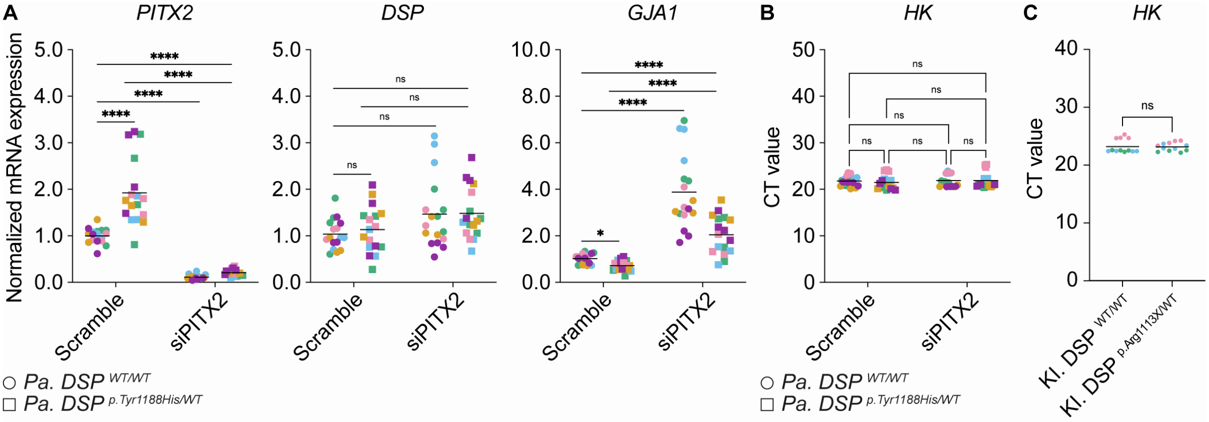

Figure S7. Knockdown of paired-like homeodomain 2 in *Pa. DSP* $^{p.Tyr1188His/WT}$ cardiomyocytes rescues the expression of structural and ion-related genes. Related to Figure 7.

(A) Gene expression levels for *PITX2*, *DSP* and *GJA1*. Values normalized to the average of *GUS*, *HARP* and *RPL32*. (B) Average cycle threshold (CT) levels for *GUS*, *HARP* and *RLP32* in one-month-old *Pa. DSP* $^{p.Tyr1188His/WT}$ cardiomyocytes treated with scramble siRNA or siRNA targeting *PITX2* for 72 h. (C) Average CT levels for *GUS* in one-month-old *KI. DSP* $^{p.Arg1113X/WT}$ cardiomyocytes (Related to Figures 7C-E). Data plotted as mean. The dots in (A-C) represent technical replicates, whereas the color of each dot indicates the experimental origin (3-5 independent experiments; 3-4 technical replicates). For (A-B), significance has been assessed on log-transformed data using an ordinary two-way ANOVA followed by a Tukey's multiple comparisons test (single pooled variance; alpha = 0.05). For (C), significance has been assessed by a two-tailed unpaired Student's t-test (* P-value < 0.05, **** P-value < 0.0001, ns: not significant). HK, housekeeping gene.

Supplemental Tables

Table S1. Clinical features of the patient.

Genetic mutation	*DSP c.3562 T>C/WT*
Protein change	DSP p.Tyr1188His/WT
Age at diagnosis	34 years
Sex	Female
Cardiac abnormalities	- Monomorphic ventricular tachycardia.<br>- Low QRS voltages.<br>- Abnormal repolarization.<br>- Akinesia in the RV apex.<br>- Late enhancement in the lateral wall of the LV.
Implant	1-chamber ICD

ACM, arrhythmogenic cardiomyopathy; DSP, desmoplakin; LV, left ventricle; RV, right ventricle; ICD, implantable cardioverter-defibrillator.

Table S2. Overview of human induced pluripotent stem cell lines used in this study.

Line	Origin	Age at biopsy	Sex	Ethnicity	Reprogramming method	Mutation - Gene	Protein change	Catalog number
Pa. DSP $^{p.Tyr1188His/WT}$	Patient - skin fibroblasts	34 years	F	Caucasian	Sendai virus	DSP c.3562T>C	p.Tyr1188His/WT	N.A.
Pa. DSP $^{WT/WT}$	Patient - skin fibroblasts	34 years	F	Caucasian	Sendai virus	N.A.	WT/WT	N.A.
KI. DSP $^{WT/WT}$	Healthy individual - bone marrow CD34+ cells	31 years	M	Caucasian	Sendai virus	N.A.	WT/WT	ATCC, ACS-1026™
KI. DSP $^{p.Tyr1188His/WT}$	Healthy individual - bone marrow CD34+ cells	31 years	M	Caucasian	N.A.	DSP c.3562T>C	p.Tyr1188His/WT	N.A.
KI. DSP $^{p.Arg1113X/WT}$	Healthy individual - bone marrow CD34+ cells	31 years	M	Caucasian	N.A.	DSP c.3337C>T	p.Arg1113X/WT	N.A.

c., chromosome; DSP, desmoplakin; F, Female; KI, knock-in; M, Male; N.A. not applicable; p., protein; Pa, patient; WT, wildtype.

Table S3. Primers and DNA templates

Name	Sequence (5’->3’)	Purpose
DSP_patient-KI_gRNA1_FW	CACCGAGGTTGAGGGTTCTACTGC	CRISPR/Cas9
DSP_patient-KI_gRNA1_RV	AAACGCAGTAGAACCCTCAACCTC	CRISPR/Cas9
DSP_patient-KI_gRNA2_FW	CACCGTACGAGATTGAAAGGTTGA	CRISPR/Cas9
DSP_patient-KI_gRNA2_RV	AAACTCAACCTTTCAATCTCGTAC	CRISPR/Cas9
DSP_patient-KI_gRNA3_FW	CACCGTAAGAAACCACTATAATG	CRISPR/Cas9
DSP_patient-KI_gRNA3_RV	AAACCATTATAGTGGTTTCTTAC	CRISPR/Cas9
DSP_patient-KI_gRNA4_FW	CACCGTACTCATCTCCTCATTATAG	CRISPR/Cas9
DSP_patient-KI_gRNA4_RV	AAACCTATAATGAGGAGATGAGTAC	CRISPR/Cas9
DSP-universal_FW	ATATGAGCAGCTGGTGCAAG	Genotyping
DSP-universal_RV	CTTGGCCTCCTCCTGAAAC	Genotyping
DSP_patient_ssODN	TGTGAAAAGGAGAACCTTGGTTGGCAGAAATTAGAGTCTGAGAAAGCCATCAAGGAGAAGGAGTACGAGATTGAAAGGTTGAGGGTTCTACTACAAGAAGAAGGTACCCGGAAGAGAGAATATGAAAATGAGCTGGCAAAGGTAAGAAACCACTATAATGAGGAGATGAGTAATTTAAGGAACAAGTATGAAACAGAGAT	DNA template – CRISPR/Cas9
DSP_KI_ssODN1	AGAAGGAGTACGAGATTGAAAGGTTGAGGGTTCTACTGCAAGAAGAAGGTACCCGGAAGAGAGAACATGAAAATGAGCTGGCAAAGGTAAGAAACCACTATAATGAGGAGATGAGTAATTTAAGGAA	DNA template – CRISPR/Cas9
DSP_KI_ssODN2	AGAAGGAGTACGAGATTGAAAGGTTGAGGGTTCTACTGCAAGAAGAAGGTACCCGGAAGAGAGAATATGAAAATGAGCTGGCAAAGGTAAGAAACCACTATAATGAGGAGATGAGTAATTTAAGGAA	DNA template – CRISPR/Cas9
Off-target 1 - DSP_patient-KI_FW	AGCTTCCCTCCCTTGACTCT	Amplification of potential off-target site
Off-target 1 - DSP_patient-KI_RV	GAAGCTACAGGGCAAGATGG	Amplification of potential off-target site
Off-target 2 - DSP_patient-KI_FW	GCTCAAAGAACAAGCTGCTGA	Amplification of potential off-target site
Off-target 2 - DSP_patient-KI_RV	GGATGATCTCATATTAAGATTTTCCAA	Amplification of potential off-target site
Off-target 3 - DSP_patient-KI_FW	GCCTCTTGGGTTCAAACAAT	Amplification of potential off-target site
Off-target 3 - DSP_patient-KI_RV	ACCCTGCAGGAGAAAAACCT	Amplification of potential off-target site

DSP; desmoplakin; FW, forward; gRNA, single guide RNA; KI, knock-in; RV, reverse; ssODN, single-stranded oligodeoxynucleotide.

Table S4. Antibodies and dilutions.

Target	Host species	Dilution / Application	Product ID	Company
Plakophilin (PKP2)	Goat	1:1000 - IB	Ab189323	Abcam
Plakophilin-2 (PKP2)	Mouse	1:100 – IF	651167	Santa Cruz
Plakoglobin (PKG)	Mouse	1:1000 - IB	sc398183	Santa Cruz
		1:500 - IF		
Desmoplakin (DSP)	Rabbit	1:1000 - IB	ab71690	Abcam
Desmoplakin (DSP)	Mouse	1:50 - IF	65146	Progen
Desmocollin (DSC)	Mouse	1:250 - IB	32-6200	ThermoFisher Scientific
Desmoglein (DSG)	Mouse	1:100 - IB	61002	Progen
Paired-like homeodomain 2 (PITX2)	Rabbit	1:1000 - IB	PA-1020-100	Capra Sciences
Connexin 43 (CX43)	Rabbit	1:2000 - IB	C6219	Sigma-Aldrich
Sodium channel protein type 5 subunit alpha (NaV1.5)	Rabbit	1:500 - IB	ASC-005	Alomone
Vinculin (VIN)	Mouse	1:1000 - IB	sc25336	Santa Cruz
Cardiac Troponin T (TNNT2)	Rabbit	1:400 - IF	ab45932	Abcam
Alpha-actinin-2 (ACTN2)	Mouse	1:400 - IF	A7811	Sigma-Aldrich
FLAG	Mouse	1:2500 - IP	F3165	Sigma-Aldrich
GFP	Mouse	1:2000 - IP	11814460001	Roche

IB, immunoblotting; IF, immunofluorescence; IP, immunoprecipitation.

Table S5. Quantitative PCR primers.

Name	Forward - sequence (5’->3’)	Reverse - sequence (5’->3’)
Desmocollin (*DSC*)	TGGTAGAGTTAACCTGAAAGAGTG	TGGTTCTCAGTGTTGGAAAGT
Desmoglein (*DSG*)	GGAACACAGCAGCTACACTT	ACCATCCCTTCAAGCACTTTAT
Desmoplakin (*DSP*)	GCACCAGCAGGATGTACTATT	TCAATTCAGGCTGCACGAT
Forkhead Box C2 (*FOXC2*)	CCTCCTGGTATCTCAACCACA	GAGGGTCGAGTTCTCAATCCC
Frizzled Class Receptor 2 (*FZD2*)	GTGCCATCCTATCTCAGCTACA	CTGCATGTCTACCAAGTACGTG
Gap junction alpha-1 protein (*GJA1*)	TTAAGGATCGGGTTAAGGGAAAG	TGTACCCAGGAGGAGACATAG
Glucuronidase Beta (*GUS*)	CCACCTAGAATCTGCTGGCTAC	GTGCCCGTAGTCGTGATACCAA
Ribosomal Protein Lateral Stalk Subunit P0 (*HARP)*	CACCATTGAAATCCTGAGTGATGT	TGACCAGCCCAAAGGAGAAG
Plakoglobin (*JUP*)	ACCAGGAGAGCAAGCTGAT	CTCCACAATGGCAGGCTTATT
Potassium Inwardly Rectifying Channel Subfamily J Member 2 (*KCNJ2*)	ACCGCTACAGCATCGTCTCT	TCCACACACGTGGTGAAGAT
Lipopolysaccharide Induced TNF Factor (*LITAF*)	ATGTCGGTTCCAGGACCTTAC	TACGAAGGAGGATTCATGCCC
Paired-like homeodomain 2 (*PITX2*)	ACTTTCCAGAGGAACCGCTAC	GTTGCGCTCCCTCTTTCTC
Plakophilin-2 (*PKP2*)	TGCTAAAGGCTGGCACAA	TAATCGCTGTGCGTGTAGTG
Ribosomal Protein L32 (*RPL32*)	CAACGTCAAGGAGCTGGAAG	TGGGGTTGGTGACTCTGATG
Sodium Voltage-Gated Channel Alpha Subunit 5 (*SCN5A*)	TCTCTATGGCAATCCACCCCA	GAGGACATACAAGGCGTTGGT
Secreted Frizzled Related Protein 4 (*SFRP4*)	CGAGCTGCCTGTCTATGACC	ATCCACTTAACATCCTCCGGG

Supplemental Experimental Procedures

Co-immunoprecipitation

HEK293T cells were grown in Dulbecc's Modified Eagl's Medium (ThermoFisher Scientific, 11965084) supplemented with 10% fetal bovine serum (Merck) and 100 U/mL penicillin-streptomycin (ThermoFisher Scientific, 15140122) on a 145 cm^2 dish until 60-70% confluency. Cells were then transfected with 25 μg of each plasmid using 1 mg/mL polyethylenimine (PEI; Polysciences, 23966). Medium was refreshed 18 h after transfection. Cells were harvested after 48 h using 7 mL cold PBS with 1 mM EDTA, followed by centrifugation at 300 RCF for 5 minutes. Supernatant was aspirated and cell pellet was resuspended in 1 mL NP-40 lysis buffer (150 mM NaCl, 50 mM Tris-HCl pH 7.6, 0.2% NP-40, 1 mM EDTA, 10% Glycerol) and incubated for 30 minutes at 4°C. After incubation, 25 μL of lysis product was collected for the input. Dynabeads™ Protein G (Invitrogen, 10004D) were coated with 2 μg/reaction of Monoclonal anti-FLAG® M2 (Sigma-Aldrich, F3165) according to the manufacturer's instructions. All washing steps were performed using PBS pH 7.4 with 0.02% Tween™ 20 (Sigma-Aldrich, P1379-1L) and the beads were coated at room temperature for 20 minutes. Coated beads were subsequently added to 1 mL of lysis product and incubated overnight at 4°C while rotating. Beads were processed according to the manufacturer's instructions. For the elution step, beads were placed on a magnetic stand followed by removal of all supernatant. Next, each reaction was incubated for 30 minutes at 4°Cwith 75 μL TBS (150 mM NaCl, 50 mM Tris-HCL pH 7.6 in water) supplemented with 25 μg/reaction 3x FLAG peptide (Sigma-Aldrich, F4799). Supernatant was transferred to a new 1.5 mL tube followed by addition of 4x loading dye (250 mM Tris, 8% SDS, 40% Glycerol, 20% 2-mercapto-ethanol). Samples were incubated for 3 minutes at 95°Cand stored at -20°C. Immunoprecipitation reactions (25 μL) together with 2 μL of input were then analyzed on a 7% SDS-PAGE gel using the antibodies listed in Table S4.

Cell culture

hiPSCs were cultured on Geltrex™ lactose dehydrogenase elevating virus-free, human embryonic stem cell-Qualified, Reduced Growth Factor Basement Membrane Matrix-coated wells (Gibco, A1413302). Cells were refreshed with Essential 8™ Medium (Gibco, A1517001) on a daily basis. hiPSCs were passaged at 80-100% confluency. Briefly, medium was aspirated and TrypLE Express Enzyme (Gibco, 12605010) was added for 5 minutes at 37°C. After incubation, 4 mL of Essential 8™ Medium, supplemented with 2 μM thiazovivin (Sigma-Aldrich, 420220), was added to the dissociated cells and transferred to a 15 mL Falcon tube. Cells were centrifuged for 3 minutes at 300 RCF. Subsequently, cells were seeded at a density of 15,000 cells/cm^2 in Essential 8™ Medium, supplemented with 2 μM thiazovivin. Medium was refreshed the next day with plain Essential 8™.

Genome targeting with CRISPR/Cas9

gRNA sequences were selected with the CCTop- CRISPR/Cas9 target online predictor tool (Labuhn et al., 2018; Stemmer et al., 2015). No changes were made to the default parameters. Four gRNAs were selected (Table S3). gRNAs were cloned into the pSpCas9(BB)-2A-GFP vector (Addgene, #48138). A T7-endonuclease assay was performed to estimate the cutting efficiency of each gRNA. Briefly, HEK293T cells were grown in a 6-wells well until a confluency of 70%. Cells were then transfected with 1 μg of each gRNA using 6 μg PEI. After 48h, genomic DNA was isolated using the DNeasy Blood and Tissue kit (Qiagen, 69506) according to the instructions of the manufacturer. Modified DNA regions were amplified by polymerase chain reaction (PCR) using GoTaq® Green Master Mix (Promega, M712) and the primers shown in Table S3. NEBuffer 2.1 (BioLabs$^{®}_{Inc}$ New England, B7202S) was added to each reaction, followed by denaturation at 95ºC for 5 minutes and ramping down the temperature to 25ºC (-5ºC/minutes). Next, T7 endonuclease I (BioLabs$^{®}_{Inc}$ New England, M0302S) enzyme was added to each reaction and incubated for 30 minutes at 37ºC. Digested products were analyzed on a 2% weight/volume agarose gel. The most effective gRNA was selected based on the cutting efficiency and its proximity to the mutation-of-interest; for which gRNAs closer to this site are predicted to be better. Next, the mutation-of-interest was introduced into hiPSCs. Briefly, hiPSCs were pretreated with Essential 8™ Medium supplemented with 2 μM thiazovivin for at least 1 hour. hiPSCs were then dissociated with TrypLE Express Enzyme for 5 minutes at 37°C. Dissociated cells were collected in 4 mL of Essential 8™ Medium, supplemented with 2 μM thiazovivin and centrifuged for 3 minutes at 300 RCF. Supernatant was removed and the cell pellet was resuspended in 1 mL of Essential 8™ Medium, supplemented with 2 μM thiazovivin. Cell number was assessed with the Countess II FL (Invitrogen, AMQAF1000). Two million cells were collected in a 15 mL Falcon tube and centrifuged for 3 minutes at 300 RCF. Supernatant was aspirated and the cell pellet was resuspended in 100 μL prewarmed (37°C)

nucleofection mix (Lonza Human Stem Cell Nucleofector™ Kit 1, VPH-5012) supplemented with 5 μg pSpCas9(BB)-2A-GFP vector containing the gRNA sequence and 1 μg of asymmetrically designed single-stranded oligodeoxynucleotides containing the mutation-of-interest (IDT ultramer; Table S3)(Richardson et al., 2016). Cells were transferred to an electroporation cuvette and placed in the Nucleofector™ device (Lonza), and were then nucleofected using the A-023 program for human stem cells. 500 μL of warm Essential 8™ Medium supplemented with 2 μM thiazovivin was added to the cuvette. Cells were then plated into two Geltrex coated wells in a 6-well plate. After 24 h, cells were dissociated as described above and single-cell sorted (BD biosciences, FACSJazz™) based on GFP expression in 96-well plates coated with irradiated mouse embryonic fibroblasts (ThermoFisher Scientific, A34961). 96-well plates were centrifuged for 1 minute at 200RCF and then incubated at 37°C. After 10-14 days, clones were dissociated for 5-10 minutes using Versene Solution (Gibco, 15040066) and passaged into two wells of a 96-well plate. Genomic DNA was isolated 1-2 days later using a standard ethanol-based precipitation method. The genomic region-of-interest of each clone was amplified using GoTaq® Green Master Mix and the primers listed in Table S3. Amplified product was then sent for Sanger sequencing (Macrogen, Amsterdam, The Netherlands).

Karyotyping
Genomic DNA integrity of modified hiPSCs was assessed by karyotyping. Cells grown in the exponential phase were incubated with 0.05 μg/mL colcemid (Gibco, 152120-012) for 90 minutes at 37ºC. Medium was collected and the cells were rinsed once with dPBS (Gibco, 14190094). Cells were then dissociated for 5 minutes using TrypLE Express Enzyme and centrifuged for 5 minutes at 300 RCF. Supernatant was removed and the cell pellet was resuspended in 1 mL of prewarmed 75 mM KCl while shaking constantly. After 10 min, 50 μL of freshly prepared methanol:acetic acid (3:1 ratio) solution was added to the cells in a dropwise manner. Cells were centrifuged for 5 minutes at 300 RCF. Supernatant was removed and the cell pellet was resuspended in 1 mL of methanol:acetic acid (3:1 ratio) solution while shaking constantly. The mixture was left at room temperature for 20 min, after which the cells were centrifuged for 5 minutes at 300 RCF. The last two steps were repeated twice. After the last centrifugation step, the cell pellet was resuspended in 0.5 mL methanol:acetic acid (3:1 ratio) solution. Drops of the cell suspension were then dropped from approximately 50 cm high on a glass microscope slide. The slides were dried in an incubator. Finally, the slides were mounted with ProLong™ Gold Antifade Mountant with DAPI (ThermoFisher Scientific, P36935). Chromosome spreads were imaged using the Leica TCS SPE Confocal Microscope.

Karyo-sequencing
Approximately 1000 hiPSCs were collected as a pellet. Five μL of 2 μg Proteinase K (NEB, P8107S) in 1x CutSmart Buffer (NEB, B6004S) was added for 2 h at 55°C followed by 10 minutes at 80°C. DNA was digested using 10 μL of 10 U NLAIII (NEB, R0125S) in 1x CutSmart Buffer for 2 h at 37°C followed by 20 minutes at 80°C. DNA fragments were ligated to adapters by adding 20 μL of 800 U T4 DNA ligase (NEB, M0202S), 1 mM ATP (ThermoFisher Scientific, R0441) and 50 nM adapter in 1x T4 DNA ligase buffer and incubating at 16°C overnight. The library preparation, sequencing and analysis was performed as described previously (Bolhaqueiro et al., 2019).

Off-targets
Top three exonic or intronic off-target sites, as predicted by the CCTop tool, were PCR amplified using GoTaq® G2 Hot Start Master Mix and the primers lister in Table S3.

Cardiomyocyte differentiation
hiPSCs were maintained as described above and grown until 80-90% confluency (Day 0). Medium was aspirated and cells were washed once with dPBS. Next, cells were fed with RPMI-1640-Medium-GlutaMAX™Supplement-HEPES (Gibco, 72400-021) supplemented with 0.5 mg/mL human recombinant albumin (Sigma-Aldrich, A9731), 0.2 mg/mL L-Ascorbic Acid 2-Phosphate (Sigma-Aldrich, A8960), and 4 μM CHIR99021 (Sigma-Aldrich, 361559). After 48 h (Day 2), medium was aspirated and cells were washed once with RPMI-1640-Medium-GlutaMAX™Supplement-HEPES, followed by addition of RPMI-1640-Medium-GlutaMAX™Supplement-HEPES supplemented with 0.5 mg/mL human recombinant albumin, 0.2 mg/mL L-Ascorbic Acid 2-Phosphate, and 5 μM IWP2 (Sigma-Aldrich, 681671). On day 4 and day 6, cells were refreshed with RPMI-1640-Medium-GlutaMAX™Supplement-HEPES supplemented with 0.5 mg/mL human recombinant albumin and 0.2 mg/mL L-Ascorbic Acid 2-Phosphate. From day 8 onwards, cells were refreshed every 3-4 days with RPMI-1640-Medium-GlutaMAX™-Supplement-HEPES supplemented with B-27™Supplement (50x)-serum free (Gibco, 17504001). hiPSC-derived cardiomyocytes were subsequently dissociated with TrypLE™ Select

Enzyme (10x) without phenol red (Gibco, A1217703) for a maximum of 45 minutes at 37°C. Cardiomyocytes were seeded at a density of 100,000 cells/cm^2 in Geltrex-coated wells for downstream molecular applications.

Cardiomyocyte purity
hiPSC-derived cardiomyocytes were dissociated with TrypLE™ Select Enzyme (10x) without phenol red. One million cardiomyocytes were centrifuged for 5 minutes at 300 RCF. Medium was removed and cells were washed once with dPBS. Cells were then resuspended in 1 mL of ice-cold 70% ethanol. After fixation, cells were centrifuged for 4 minutes at 300 RCF, followed by aspiration of the fixative. Cells were resuspended in PBS (pH7.2-7.4) supplemented with 5% fetal bovine serum, 1% BSA, and 0.5% Triton X-100 (blocking buffer) and permeabilized for 10 minutes on ice. Permeabilized cells were centrifuged for 4 minutes at 300 RCF and the supernatant was aspirated. The cell pellet was resuspended in 100 μL blocking buffer supplemented with anti-Cardiac Troponin T antibody and incubated for 1 hour at 4°C. After 1 hour, 500 μL of blocking solution was added and the cells were centrifuged for 4 minutes at 300 RCF. Supernatant was aspirated and cells were resuspended once more in 500 μL blocking buffer, followed by centrifugation for 4 minutes at 300 RCF and resuspension of the cells in 100 μL blocking buffer supplemented with Alexa 488-anti-rabbit antibody. After 30 minutes incubation at room temperature, 500 μL of blocking buffer was added to the cells, followed by a centrifugation step of 4 minutes at 300 RCF. The supernatant was removed and cells were resuspended again with 500 μL of blocking buffer, followed by another centrifugation for 4 minutes at 300 RCF. Lastly, the supernatant was aspirated and the cells were resuspended in 1 mL of dPBS and analyzed by FACS (BD biosciences, FACSCalibur™). Antibodies and the dilutions used can be found in Table S4.

Electrophysiology
hiPSC-derived cardiomyocytes were seeded per 100,000 cells on Geltrex-coated glass coverslips, to allow cluster formation. Coverslips were incubated for 15 minutes at 37°Cwith 1:1000 voltage sensitive dye FluoVolt and 1:1000 Powerload (Thermo Fischer Scientific, F10488) in RPMI-1640-Medium-GlutaMAX™-Supplement-HEPES supplemented with B-27™Supplement (50x)-serum free. During measurements, cells were immersed in a solution containing in mM: NaCl (130), KCl (4), CaCl2 (1.8), MgCl2 (1.2), NaHCO3 (18), HEPES (10) and glucose (10), pH 7.4. A custom-build microscope (Cairn Research, Kent, UK) with a 10x objective was used for the recordings. Excitation of the dye was done by a blue light, using a 482/35 excitation filter (Semrock FF01-482/35-25), and captured, using a 514 long-pass emission filter (Semrock LP02-514RU-25), with a high-speed camera (Andor Zyla 5.5.CL3, Oxford Instruments). A custom MATLAB script was used for analysis of the action potentials (Boer). Action potential durations were corrected for the beating rate using an adjustment of the Fredericia formula used to correct the QT interval for heart rate: APDcorrected=APD/($\sqrt[3]{}$(60/BPM))(Blinova et al., 2017).

Immunoblotting
hiPSC-derived cardiomyocytes were dissociated with TrypLE™ Select Enzyme (10x) and collected in a 1.5 mL Eppendorf tube. Cells were centrifuged for 5 minutes at 300 RCF. Supernatant was removed and cells were resuspended in 1 mL dPBS followed by centrifugation at 300 RCF for 5 min. Cells were lysed in RIPA buffer (50 mM Tris-pH7.5, 150 mM NaCl, 0.1% SDS, 0.5% sodium deoxycholate, 1% Triton X-100) supplemented with one tablet of cOmplete™ EDTA-free Protease Inhibitor Cocktail (Roche, 11836170001) and one tablet of PhosSTOP™ (Roche, 4906837001) per 10 mL of RIPA buffer. Immunoblotting was performed using 10-15 μg of protein extract. For detection, the corresponding secondary antibody coupled to horseradish peroxidase was used in combination with the Clarity™ Western ECL Substrate kit (Bio Rad, 1705061). Immunoblots were imaged with an Amersham Imager 680RGB device (GE Healthcare, 29270772) and quantified with ImageJ. Antibodies and the dilutions used can be found in Table S4.

Immunocytochemistry
hiPSC-derived cardiomyocytes were seeded on Geltrex-coated glass coverslips with a diameter of 12 mm. Seven days later, cells were washed once with dPBS and then fixed with 2% paraformaldehyde for 30 minutes at room temperature. Cardiomyocytes were washed 3x with PBS and permeabilized with 0,1% Triton X-100 in PBS for 8 minutes at room temperature. Permeabilized cells were blocked with 4% goat serum in PBS for 1 hour at room temperature. Cells were then incubated with primary antibody diluted in 4% goat serum solution and incubated overnight at 4°C. Next, cells were washed three times with PBS and incubated with the corresponding secondary antibody dissolved in 4% goat serum solution for 1 hour at room temperature. Nuclei were stained with DAPI for 5 minutes at room

temperature. Cells were mounted with Mowiol (24% (w/v) Glycerol (Baker, 7044), 9.6% (w/v) Mowiol 4-88 (Calbiochem, 475904), 0.1 M Tris-HCl pH 8.5) and imaged using a Leica TCS SPE Confocal Microscope. Antibodies and the dilutions used can be found in Table S4.

Quantitative PCR

Total RNA was isolated from hiPSC-derived cardiomyocytes using the RNeasy Mini Kit (Qiagen, 74104) following the protocol supplied by the manufacturer. Total RNA was reverse transcribed using the iScript™ cDNA Synthesis Kit (Bio Rad, 1708891). Quantitative PCR (qPCR) reactions were performed on a Bio Rad CFX96 Real-Time PCR Detection System using the iQ SYBR Green Supermix kit (Bio Rad, 170-8885). Primers used for amplification can be found in Table S5. The ΔΔCt-method was used to analyze the data. Control groups were set at 1. Gene expression data presented in Figure 7 and Figure S8 were corrected for session-to-session variation prior to statistical analyses using a previously published method (Ruijter et al., 2006).

mRNA-sequencing

mRNA-sequencing was performed on one-month-old hiPSC-derived *Pa. DSP* $^{WT/WT}$, *Pa. DSP* $^{p.Tyr1188His/WT}$, *KI. DSP* $^{WT/WT}$ and *KI. DSP* $^{p.Tyr1188His/WT}$ cardiomyocytes (four technical replicates per line). Total RNA isolation was performed as described above. RNA libraries were prepared with the TruSeq Stranded mRNA polyA kit (Illumina) according to the manufacturer's protocol. Strand-specific single-end 75 bp reads were generated on an Illumina NextSeq 500 system. On average, we obtained 33.000.000 reads per sample, which were used for subsequent analysis. Reads were checked for their quality using FastQC, and aligned against the human genome (assembly GRCh37) using STAR (STAR_2.4.2a). Counting aligned sequencing reads per gene was done with the HTSeq-count package using the union mode. Finally, differential expression was calculated using DESeq2 v1.2 with pooled dispersion estimates (Love et al., 2014). Genes were considered significantly different when FoldChange > 2.0 or < -2.0 and p adjusted < 0.05.

Enrichment analysis

Differentially expressed genes, obtained with the DESeq2 pipeline, were further analyzed for functional enrichment using the STRINGv11.5 database (Szklarczyk et al., 2021). No changes were made to the default settings. Homo sapiens served as background. Only the molecular function (GO-MF), biological process (GO-BP), cellular component (GO-CC), and KEGG data sources were considered for enrichment analysis.

Knockdown experiments

Three-week-old hiPSC-derived cardiomyocytes were dissociated using TrypLE™ Select Enzyme (10x), no phenol red and seeded at a cell density of 100,000 cells/cm^2 in Geltrex-coated wells with RPMI-1640-Medium-GlutaMAX™Supplement-HEPES supplemented with B-27™ Supplement (50x)-serum free and 2 µM thiazovivin. After 24 h, medium was refreshed with RPMI-1640-Medium-GlutaMAX™Supplement-HEPES supplemented with B-27™ Supplement (50x)-serum free. Seven days after reseeding, cells were transfected with either 10 nM scramble or siRNA oligo duplexes targeting *PITX2* (OriGene, SR321325) utilizing Lipofectamine™ RNAiMAX (ThermoFisher, 13778150) according to the manufacturers' instructions. RNA and protein samples were collected as described above after 48 h and 72 h, respectively.

Overexpression experiments

Two-week-old hiPSC-derived cardiomyocytes were dissociated using TrypLE™ Select Enzyme (10x), no phenol red and seeded at a cell density of 100,000 cells/cm^2 in Geltrex-coated wells with RPMI-1640-Medium-GlutaMAX™Supplement-HEPES supplemented with B-27™ Supplement (50x)-serum free and 2 µM thiazovivin. After 24 h, medium was refreshed with RPMI-1640-Medium-GlutaMAX™Supplement-HEPES supplemented with B-27™ Supplement (50x)-serum free. Seven days after reseeding, cells were infected with either PITX2C (ENST00000644743.1) cloned into the pLVX-IRES-Hygro (Clontech, 632185) vector backbone or pLVX-EGFP vector. After seven days, RNA and protein samples were collected (as described above). The viral particles used were harvested 48 h after transfection of 7x10^6 HEK293X cells with 7 µg pLVX-IRES-Hygro or pLVX-EGFP together with 8 µg psPAX2 (Addgene, #12260) and 3 µg pMD2G (Addgene, #12259).

Human material

The study fulfilled the Dutch criteria of the code of proper use of human tissue. Written informed consent was obtained for the generation and use of the patient-derived hiPSCs.

Supplemental References

Blinova, K., Stohlman, J., Vicente, J., Chan, D., Johannesen, L., Hortigon-Vinagre, M.P., Zamora, V., Smith, G., Crumb, W.J., Pang, L., et al. (2017). Comprehensive translational assessment of human-induced pluripotent stem cell derived cardiomyocytes for evaluating drug-induced arrhythmias. Toxicol. Sci. *155*, 234–247.

Boer, T. de Peaks, software for analysing cardiac APs and calcium transients.

Bolhaqueiro, A.C.F., Ponsioen, B., Bakker, B., Klaasen, S.J., Kucukkose, E., van Jaarsveld, R.H., Vivié, J., Verlaan-Klink, I., Hami, N., Spierings, D.C.J., et al. (2019). Ongoing chromosomal instability and karyotype evolution in human colorectal cancer organoids. Nat. Genet. *51*, 824–834.

Labuhn, M., Adams, F.F., Ng, M., Knoess, S., Schambach, A., Charpentier, E.M., Schwarzer, A., Mateo, J.L., Klusmann, J.H., and Heckl, D. (2018). Refined sgRNA efficacy prediction improves largeand small-scale CRISPR-Cas9 applications. Nucleic Acids Res. *46*, 1375–1385.

Love, M.I., Huber, W., and Anders, S. (2014). Moderated estimation of fold change and dispersion for RNA-seq data with DESeq2. Genome Biol. *15*, 550.

Richardson, C.D., Ray, G.J., DeWitt, M.A., Curie, G.L., and Corn, J.E. (2016). Enhancing homology-directed genome editing by catalytically active and inactive CRISPR-Cas9 using asymmetric donor DNA. Nat. Biotechnol. *34*, 339–344.

Stemmer, M., Thumberger, T., del Sol Keyer, M., Wittbrodt, J., and Mateo, J.L. (2015). CCTop: An Intuitive, Flexible and Reliable CRISPR/Cas9 Target Prediction Tool. PLoS One *10*, e0124633.

Szklarczyk, D., Gable, A.L., Nastou, K.C., Lyon, D., Kirsch, R., Pyysalo, S., Doncheva, N.T., Legeay, M., Fang, T., Bork, P., et al. (2021). The STRING database in 2021: Customizable protein-protein networks, and functional characterization of user-uploaded gene/measurement sets. Nucleic Acids Res. *49*, D605–D612.
