# Supplementary material for: PITX2 induction leads to impaired cardiomyocyte function in arrhythmogenic cardiomyopathy
Source: Stem Cell Reports. 2023 Mar 2;18(3):749–64. doi: 10.1016/j.stemcr.2023.01.015 (PMC10031305; doi:10.1016/j.stemcr.2023.01.015)
Supplement: Document S2. Article plus supplemental information [file mmc2.pdf]

Stem Cell Reports
Article
ISSCR
OPEN ACCESS

PITX2 induction leads to impaired cardiomyocyte function in arrhythmogenic cardiomyopathy

Sebastiaan J. van Kampen,[1] Su Ji Han,[1] Willem B. van Ham,[2] Eirini Kyriakopoulou,[1] Elizabeth W. Stouthart,[1] Birgit Goversen,[2,3] Jantine Monshouwer-Kloots,[1] Ilaria Perini,[1] Hesther de Ruiter,[1] Petra van der Kraak,[4] Aryan Vink,[4] Linda W. van Laake,[5] Judith A. Groeneweg,[5] Teun P. de Boer,[2] Hoyee Tsui,[1] Cornelis J. Boogerd,[1] Toon A.B. van Veen,[2] and Eva van Rooij[1,5,*]

[1]Hubrecht Institute, Royal Netherlands Academy of Arts and Sciences (KNAW) and University Medical Center Utrecht, Uppsalalaan 8, 3584 CT Utrecht, the Netherlands
[2]Department of Medical Physiology, University Medical Center Utrecht, Utrecht, the Netherlands
[3]Department of Physiology, Amsterdam University Medical Centers, Amsterdam Cardiovascular Sciences, Location VU Medical Center, the Netherlands
[4]Department of Pathology, University Medical Centre Utrecht, Utrecht, the Netherlands
[5]Department of Cardiology, University Medical Center Utrecht, Utrecht, the Netherlands
*Correspondence: e.vanrooij@hubrecht.eu
https://doi.org/10.1016/j.stemcr.2023.01.015

SUMMARY

Arrhythmogenic cardiomyopathy (ACM) is an inherited progressive disease characterized by electrophysiological and structural remodeling of the ventricles. However, the disease-causing molecular pathways, as a consequence of desmosomal mutations, are poorly understood. Here, we identified a novel missense mutation within desmoplakin in a patient clinically diagnosed with ACM. Using CRISPR-Cas9, we corrected this mutation in patient-derived human induced pluripotent stem cells (hiPSCs) and generated an independent knockin hiPSC line carrying the same mutation. Mutant cardiomyocytes displayed a decline in connexin 43, NaV1.5, and desmosomal proteins, which was accompanied by a prolonged action potential duration. Interestingly, paired-like homeodomain 2 (PITX2), a transcription factor that acts a repressor of connexin 43, NaV1.5, and desmoplakin, was induced in mutant cardiomyocytes. We validated these results in control cardiomyocytes in which *PITX2* was either depleted or overexpressed. Importantly, knockdown of *PITX2* in patient-derived cardiomyocytes is sufficient to restore the levels of desmoplakin, connexin 43, and NaV1.5.

INTRODUCTION

Desmosomes are essential multiprotein complexes localized at the intercalated disc, physically connecting the intermediate filament (IF) networks of neighboring cardiomyocytes (Vermij et al., 2017). Besides providing resilience against mechanical forces during cardiac contraction, the desmosomes also play a crucial role in signal transduction (Chen et al., 2014; Garcia-Gras et al., 2006). The desmosomes encompass five essential components, the cadherins desmoglein (DSG) and desmocollin (DSC), the armadillo proteins plakoglobin (JUP) and plakophillin-2 (PKP2), and desmoplakin (DSP). The classical view of desmosomes is that they operate as a single functioning structure. However, recent studies demonstrated a close connection between desmosomes, adherens junctions, gap junctions, and ion channels, together forming the area composita (Jansen et al., 2012; Veeraraghavan and Gourdie, 2016; Vermij et al., 2017).

Mutations in desmosomal genes are frequently identified as an underlying cause of cardiovascular disease, notably in patients diagnosed with arrhythmogenic cardiomyopathy (Basso et al., 2018; Marcus et al., 1982). This progressive condition is characterized by loss of cardiomyocytes, gradual replacement of the myocardium by fibro-fatty deposits, and life-threatening ventricular arrhythmias (Hoorntje et al., 2017). Patients bearing mutations in *DSP* frequently present a complex clinical phenotype characterized by a wide range of cardiac abnormalities (Gao et al., 2020; Wang et al., 2022). To date, a number of pivotal studies focused on the role of DSP in arrhythmogenic cardiomyopathy (ACM) pathogenesis. In one study, cardiomyocyte-specific overexpression of the *DSP p.Arg2834His* mutation resulted in increased levels of apoptotic cardiomyocytes, lipid accumulation, cardiac fibrosis, and dysfunction (Yang et al., 2006). Interestingly, and in concordance with the clinical phenotype, these mice displayed accelerated ACM pathogenesis when exposed to endurance exercise, which could be linked to aberrant Wnt/β-catenin signaling (Martherus et al., 2016). Similarly, cardiomyocyte-restricted deletion of one *Dsp* allele in mice led to accumulation of JUP in the nucleus, where it competes with β-catenin, subsequently affecting Wnt-signaling pathways (Garcia-Gras et al., 2006). In addition to an impaired Wnt/β-catenin signaling axis, diminished DSP levels lead to cardiac conductance abnormalities due to mislocalization and reduced levels of connexin 43 (CX43) and sodium voltage-gated channel, alpha subunit 5 (NaV1.5) at the intercalated disc, indicating that DSP plays a role in maintaining CX43 and NaV1.5 stability in cardiomyocytes (Gomes et al., 2012; Gusev et al., 2020; Lyon et al., 2014; Zhang et al., 2013). Even though these studies

This is an open access article under the CC BY license (http://creativecommons.org/licenses/by/4.0/).

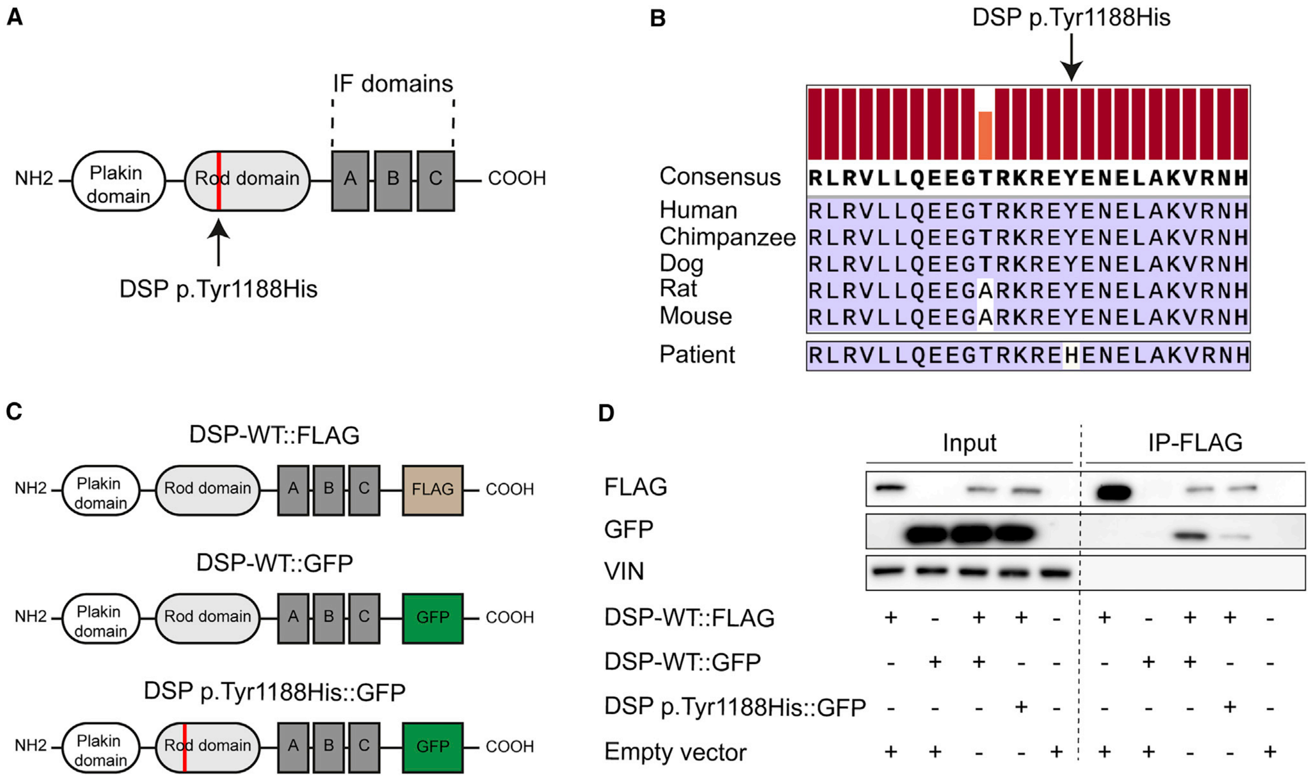

Figure 1. The novel missense mutation p.Tyr1188His in desmoplakin affects its homodimerization properties
(A) Schematic of DSP displaying the functional domains.
(B) Alignment of the protein sequence-of-interest for the indicated species. The height of the orange and red bars above each amino acid indicates the degree of conservation.
(C) Overview of the constructs used to assess the binding properties between wild-type (WT) and mutant DSP molecules.
(D) Representative immunoblots for FLAG, GFP, and vinculin (VIN) after co-immunoprecipitation of DSP-WT::FLAG in HEK293 cells transfected with the indicated conditions.
(A–C) The novel mutation is either indicated in red or with an arrow. IF, intermediate filament; IP, immunoprecipitation.

significantly increased our understanding of DSP-driven ACM, current treatments focus on relieving the symptoms rather than curing the disease due to a lack of therapeutic targets.

To contribute to a better understanding of the pathomolecular mechanisms at play, we used human induced pluripotent stem cell (hiPSC)-derived cardiomyocytes to extensively study a novel heterozygous missense mutation in *DSP* (gene: c.3562T>C; protein: p.Tyr1188His), identified in a patient with the clinical diagnosis of ACM. Compared with wild-type cardiomyocytes, we observed a prolonged action potential duration (APD) and reduced expression of desmosomal components, which was paralleled by abnormal levels of CX43 and NAV1.5. Strikingly, the transcription factor paired-like homeodomain 2 (PITX2), which acts as a repressor of structural and ion channel-related genes, was induced in these cells. Knockdown of *PITX2* in mutant cardiomyocytes led to restoration of DSP, CX43, and NaV1.5. Together, we identified *DSP c.3562T>C* as a novel pathogenic mutation and demonstrated that aberrant levels of PITX2 in response to mutant DSP might contribute to the electrophysiological abnormalities often seen in patients with ACM.

RESULTS

A novel genetic missense mutation in *DSP* alters its homodimerization properties

In a patient diagnosed with ACM and experiencing monomorphic ventricular tachycardia, abnormal repolarization, and akinesia of the right ventricular apex (Table S1), we identified a novel missense mutation within the *DSP* gene (*c.3562T>C*) that translates to an amino acid substitution of a conserved tyrosine to a histidine (p.Tyr1188His; Figures 1A and 1B). No other mutations in cardiac genes were detected. As this mutation resides within the homodimerization domain (ROD domain) of DSP, we

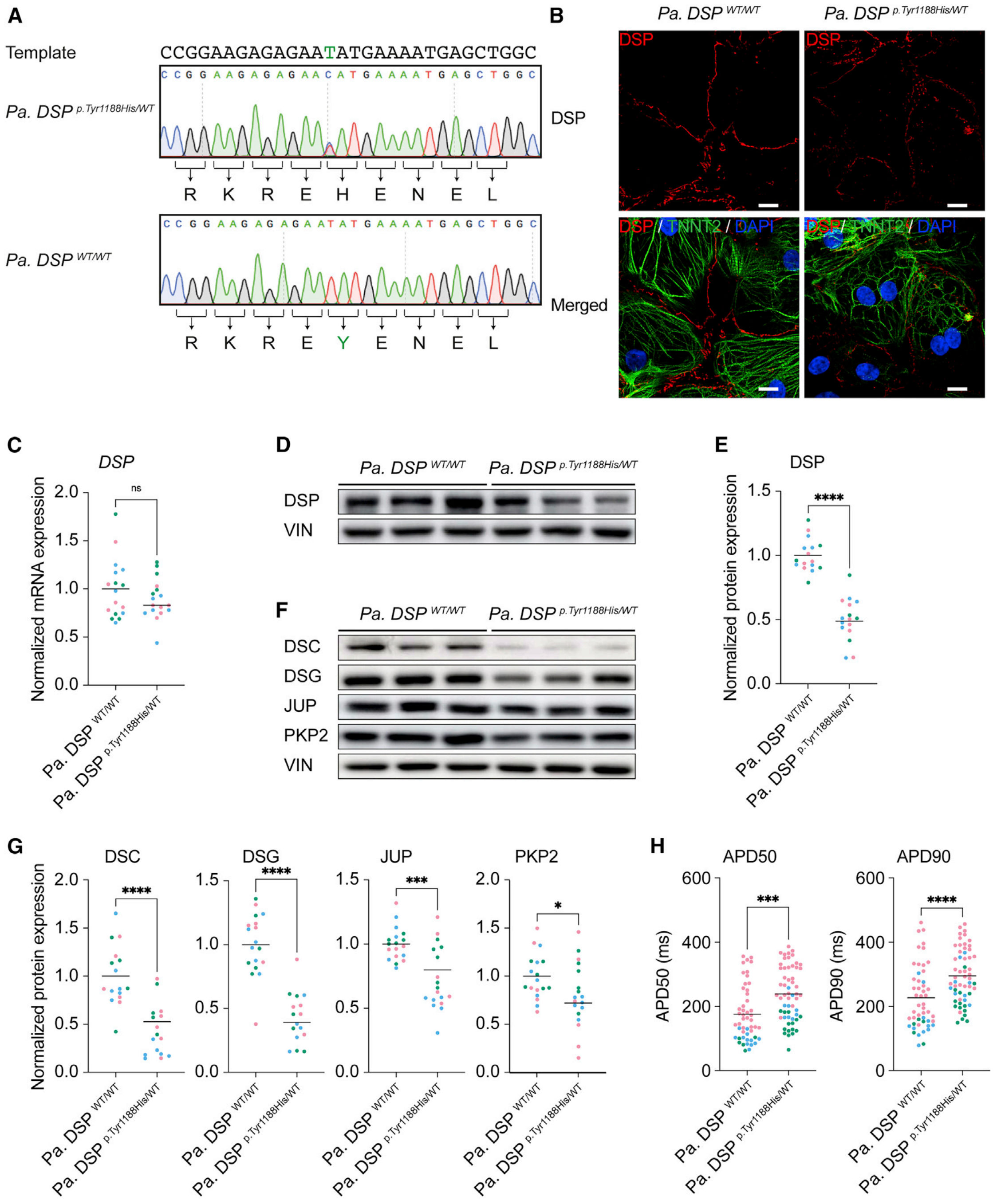

Figure 2. Heterozygous *Pa. DSP*$^{p.Tyr1188His/WT}$ hiPSC-derived cardiomyocytes display reduced desmosomal protein levels and a prolonged action potential duration

(A) Sanger sequencing traces of the patient-derived and corrected isogenic control hiPSC lines. The DNA template used for CRISPR-Cas9-targeting, correcting the *DSP*$^{p.Tyr1188His}$ mutation (C > T), is shown. Intended mutation is indicated in green.

(B–H) Molecular and functional analyses on 1-month-old hiPSC-derived cardiomyocytes obtained from three independent experiments.

(B) Representative immunostainings for DSP. DSP in red; cardiac troponin T (TNNT2) in green; DAPI in blue. Scale bar: 10 μm.

(C) Gene expression levels of *DSP* normalized to *GUS*.

(D) Representative immunoblots for DSP.

(E) Quantification of DSP protein levels normalized to VIN.

(F) Representative immunoblots for DSC, DSG, JUP, and PKP2.

(legend continued on next page)

hypothesized that this would affect its homodimerization properties (Green et al., 1990; O'Keefe et al., 1989). To study the consequences of this *DSP p.Tyr1188His* mutation on homodimerization, we generated three constructs; two in which wild-type *DSP* is fused to either FLAG (DSP-WT:FLAG) or GFP (DSP-WT::GFP) and one encoding mutant *DSP* fused to GFP (DSP p.Tyr1188His::GFP; Figure 1C). Next, we transfected these constructs into HEK293 cells and performed FLAG pull-down assays after 48 h. Immunoblotting against FLAG and DSP revealed that mutant DSP protein can still interact with WT DSP, albeit with a lower binding affinity when compared with DSP-WT::FLAG co-transfected with DSP-WT::GFP (Figure 1D). These data indicate that the novel missense mutation in *DSP* affects the homodimerization properties of DSP molecules.

Heterozygous *DSP p.Tyr1188His* hiPSC-derived cardiomyocytes display reduced desmosomal protein levels and impaired function

To further understand the molecular consequences of this novel mutation, we reprogrammed patient skin fibroblasts to obtain hiPSCs bearing the heterozygous *DSP c.3562T>C* mutation (Table S2). Next, we corrected the mutant allele utilizing CRISPR-Cas9 in combination with a single-stranded DNA template, yielding an isogenic control line (Figures 2A and S1A). Hereinafter, we refer to these lines as *Pa. DSP*$^{WT/WT}$ and *Pa. DSP*$^{p.Tyr1188His/WT}$. An additional synonymous mutation (blocking mutation) was included in the template to prevent recutting by Cas9 (Figure S1B). No genomic changes were observed for the top three predicted off-targets; ferredoxin 2 (*FDX1L*), integrator complex subunit 8 (*INTS8*), and zinc finger FYVE-type containing 27 (*ZFYVE27*; Figure S1C). The pluripotency markers Nanog homeobox (NANOG), POU class 5 homeobox 1 (OCT3/4), and SRY-box transcription factor 2 (SOX2) were expressed in *Pa. DSP*$^{WT/WT}$ and *Pa. DSP*$^{p.Tyr1188His/WT}$ hiPSCs (Figure S1D), and no changes in the karyotype were identified (Figures S1E and S1F). Directed differentiation of hiPSCs to cardiomyocytes yielded comparable percentages (90%–98%) of cardiac troponin T positive cells for both lines (Figure S2A). DSP, JUP, and PKP2 correctly localized to the cell periphery in 1-month-old mutant cardiomyocytes (Figures 2B, S2B, and S2C). However, molecular analyses revealed a significant reduction in DSP protein levels in mutant cells, whereas the mRNA levels were unaffected (Figures 2C–2E and S2D). Immunoblot analysis for desmosomal components DSC, DSG, JUP, and PKP2 showed a significant decline for all proteins in *Pa. DSP*$^{p.Tyr1188His/WT}$ hiPSC-derived cardiomyocytes compared with the isogenic control (Figures 2F and 2G). Since the patient displayed abnormalities in the cardiac conduction system including repolarization irregularities and arrhythmias, we performed electrophysiology assays on mutant hiPSC-derived cardiomyocytes. We observed a prolonged APD at 50% and 90% of repolarization in mutant cardiomyocytes compared with control (Figure 2H). Together, cardiomyocytes bearing the novel *DSP*$^{p.Tyr1188His}$ mutation show reduced desmosomal protein levels and a prolonged APD.

Wnt-signaling- and ion-handling-related processes are affected in *Pa. DSP*$^{p.Tyr1188His/WT}$ cardiomyocytes

Next, we aimed to identify affected signaling cascades that could contribute to the observed phenotype. To this end, we performed mRNA sequencing on 1-month-old *Pa. DSP*$^{WT/WT}$ and *Pa. DSP*$^{p.Tyr1188His/WT}$ cardiomyocytes. Mutant cardiomyocytes displayed a different expression profile compared with the isogenic control (Figures 3A and 3B), with 662 up- and 843 downregulated genes (log2 fold change >1 and <−1; adjusted p value [p-adj] < 0.05; Figure 3B). Gene Ontology (GO) analysis of upregulated genes identified non-canonical Wnt signaling (GO: 0035567) as enriched term (Figure 3C). Interestingly, Wnt signaling has previously been linked to ACM pathogenesis (Garcia-Gras et al., 2006). Among the gene hits for this term frizzled-2 (*FZD2*) and secreted frizzled-related protein 4 (*SFRP4*) were identified, for which the induced expression levels were validated in three independent differentiations (Figure 3D). Enriched GO terms for the downregulated genes included "cell adhesion" (GO: 0007155), "signal transduction" (GO: 0007165), and "regulation of ion transmembrane transport" (GO: 0034765; Figure 3E). Strikingly, gap junction alpha-1 protein (*GJA1*) and *SCN5A*, encoding for CX43 and NaV1.5, were among the significantly downregulated genes, which was validated in three additional batches of cardiomyocytes (Figure 3F). The aberrant expression of *GJA1* and *SCN5A* suggests that the *DSP*$^{p.Tyr1188His/WT}$ mutation not only affects structural

(G) Quantification of the desmosomal protein levels. Values normalized to VIN.

(H) Action potential duration (APD) measured at 50% and 90% of cardiomyocyte repolarization (*Pa. DSP*$^{WT/WT}$, n = 50 cell clusters; *Pa. DSP*$^{p.Tyr1188His/WT}$, n = 58 cell clusters).

Data are plotted as mean. The dots in (C), (E), (G), and (H) represent technical replicates, whereas the color of each dot indicates the experimental origin (3 independent experiments; 4–34 technical replicates). Significance has been assessed by a two-tailed unpaired Student's t test or two-tailed Mann-Whitney test when data were not normally distributed (*p < 0.05, ***p < 0.001, ****p < 0.0001, ns, not significant).

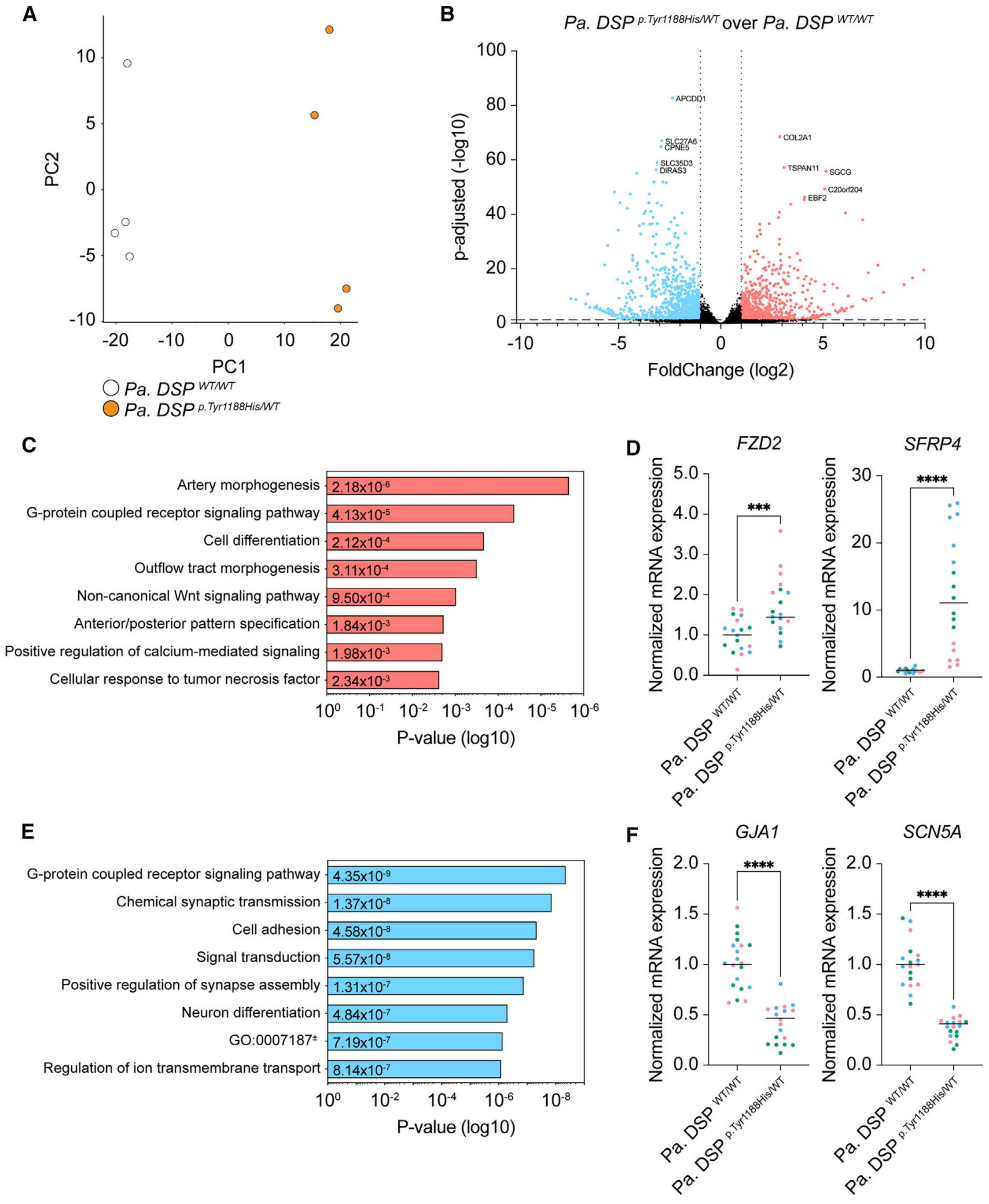

Figure 3. Gene networks related to Wnt signaling and ion handling are dysregulated in heterozygous *Pa. DSP*$^{p.Tyr1188His/WT}$ hiPSC-derived cardiomyocytes

(A–F) mRNA sequencing analyses on 1-month-old hiPSC-derived cardiomyocytes.

(A) Principal-component analysis (PCA) for *Pa. DSP*$^{WT/WT}$ and *Pa. DSP*$^{p.Tyr1188His/WT}$ cardiomyocytes.

(B) Volcano plot showing the up- and downregulated genes (fold change (log2) >1 and <−1; p-adj < 0.05) in *Pa. DSP*$^{p.Tyr1188His/WT}$ and control cardiomyocytes. The top five up- and downregulated genes are indicated.

(C) Gene Ontology analysis on the upregulated (fold change (log2) >1) genes.

(legend continued on next page)

components but also cellular processes related to signal propagation and contraction.

Knockin hiPSC-derived *DSP*$^{p.Tyr1188His/WT}$ cardiomyocytes corroborate findings observed in patient-derived cardiomyocytes

In an effort to exclude confounding effects, such as the presence of second genomic hits, we generated an independent knockin (KI) hiPSC line bearing the *DSP*$^{p.Tyr1188His/WT}$ mutation. We used a healthy hiPSC line and followed the same targeting process as described above and added a second single-stranded DNA template containing only the blocking mutation (Figures S3A and 4A). Hereafter, we refer to these lines as *KI. DSP*$^{WT/WT}$ and *KI. DSP*$^{p.Tyr1188His/WT}$. Amplification of potential off-target sites did not reveal any editing events (Figure S3B). Furthermore, chromosome integrity was unaffected, and the pluripotency markers NANOG, OCT3/4, and SOX2 were expressed in both lines (Figures S3C–S3E). Directed differentiation of control and *KI. DSP*$^{p.Tyr1188His/WT}$ cells yielded comparable percentages (75%–82%) of cardiac troponin T-positive cells (Figure S4A). Molecular analyses revealed normal localization of DSP, JUP, and PKP2 in 1-month-old mutant cardiomyocytes (Figures S4B–S4D). Similar to *Pa. DSP*$^{p.Tyr1188His/WT}$, a decline in DSP protein levels was observed in the *KI. DSP*$^{p.Tyr1188His/WT}$ cardiomyocytes (Figures 4C and 4D). Interestingly, also the mRNA levels of *DSP* were reduced, which is in contrast to the patient-derived line (Figures 4B and S4E). Western blot analysis revealed a significant decline for DSC and JUP, whereas DSG and PKP2 were expressed at a similar level compared with the isogenic control (Figures 4E and 4F). Importantly, the APD at 50% and 90% of cardiomyocyte repolarization was also prolonged in *KI. DSP*$^{p.Tyr1188His/WT}$ cardiomyocytes (Figure 4G). mRNA sequencing on 1-month-old *KI. DSP*$^{p.Tyr1188His/WT}$ and control cardiomyocytes revealed a distinct expression profile for mutant cardiomyocytes with 1,378 and 2,499 genes up- and downregulated, respectively (log2 fold change >1 and <−1; p-adj < 0.05; Figures 5A and S5A). GO term analysis for the differentially expressed genes revealed "regulation of transcription" and "ion handling" as enriched terms (Figures S5B and S5D). Significant upregulation of the transcription factors forkhead box C2 (*FOXC2*) and lipopolysaccharide induced TNF factor (*LITAF*) was confirmed in three additional batches of differentiated cardiomyocytes (Figure S5C). In line with our observations in the patient-derived line, we noticed dysregulation of ion channels including potassium inwardly rectifying channel subfamily J member 2 (*KCNJ2*) and *SCN5A* (Figure S5E). Together, characterization of the *KI. DSP*$^{p.Tyr1188His/WT}$ line corroborated the data collected from *Pa. DSP*$^{p.Tyr1188His/WT}$ cardiomyocytes, demonstrating that this novel missense mutation in *DSP* is responsible for eliciting the observed molecular and functional changes.

Combinatorial mRNA sequencing analysis of *Pa.* and *KI. DSP*$^{p.Tyr1188His/WT}$ cardiomyocytes reveals impaired cardiac muscle cell depolarization

To identify the molecular changes specifically caused by the novel *DSP*$^{p.Tyr1188His}$ mutation and not due to the genetic background of the lines, we performed a combinatorial analysis on the differentially expressed genes obtained from the patient (Figure 3) and KI (Figure S5) datasets. In addition to these gene lists, we also included a set of differentially expressed genes obtained from an mRNA sequencing analysis in which we combined all WT and mutant samples from both datasets ("combined patient and KI"). We identified a subset of genes that were consistently differentially expressed between the patient and KI datasets (Figures 5B and 5C). Next, we performed GO analyses on the 104 up- and 275 downregulated genes shared between all comparisons (Figures 5B–5E). The significantly upregulated genes were associated with the Wnt-signaling pathway, whereas the shared downregulated genes were enriched for "regulation of ion transmembrane transport" and "regulation of ventricular cardiac muscle cell depolarization" (Figures 5D and 5E). Next, we performed a string-db analysis using the genes within the GO terms "regulation of ion transmembrane transport" and "regulation of ventricular cardiac muscle cell depolarization," accentuating the presence of genes fundamental for cardiomyocyte function such as *GJA1* and *SCN5A*. Reduced expression of *GJA1* and *SCN5A* was validated in three additional differentiations of 1-month-old *Pa. DSP*$^{p.Tyr1188His/WT}$ and *KI. DSP*$^{p.Tyr1188His/WT}$ cardiomyocytes (Figures 3F, S5E, and S5F). Immunoblot analysis confirmed that CX43 and NaV1.5 protein levels were reduced in *Pa. DSP*$^{p.Tyr1188His/WT}$ and *KI. DSP*$^{p.Tyr1188His/WT}$ cardiomyocytes compared with control cells (Figures 5G–5I).

(D) Validation of *FZD2* and *SFRP4*, both belonging to the "non-canonical Wnt-signaling pathway" term.
(E) Gene Ontology analysis on the downregulated (fold change (log2) <−1) genes.
(F) Validation of *GJA1* and *SCN5A*, both belonging to the "regulation of ion transmembrane transport" term. mRNA sequencing was performed on four replicates obtained from one hiPSC differentiation.
The dots in (D) and (F) represent technical replicates, whereas the color of each dot indicates the experimental origin (3 independent experiments; 4–8 technical replicates). Gene expression data are normalized to *GUS* and plotted as mean. Significance has been assessed by a two-tailed unpaired Student's t test (***p < 0.001, ****p < 0.0001). GO: 0007187, G protein-coupled receptor signaling pathway coupled to cyclic nucleotide second messenger.

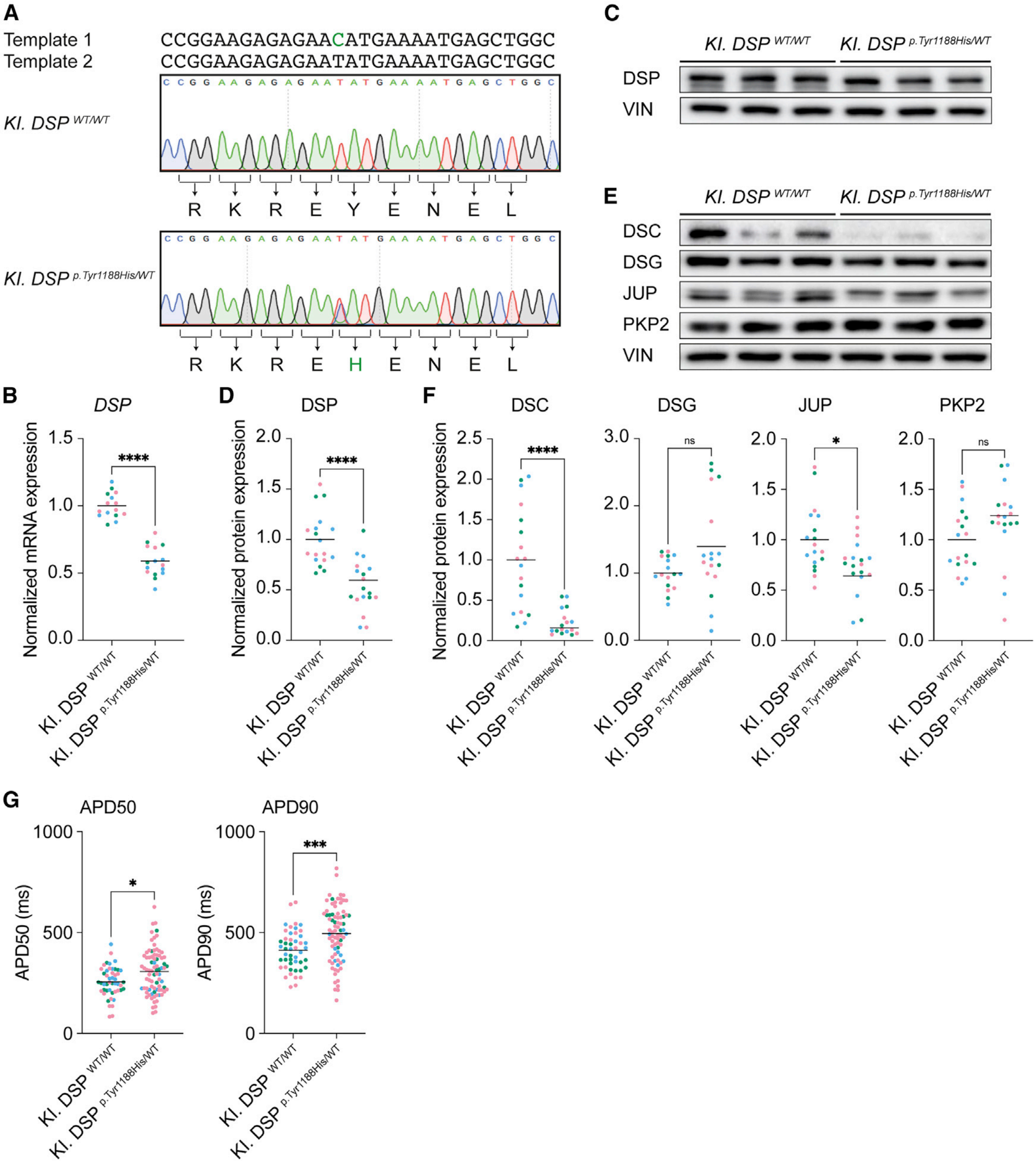

Figure 4. hiPSC-derived *KI. DSP*$^{p.Tyr1188His/WT}$ cardiomyocytes corroborate findings observed in patient-derived cardiomyocytes

(A) Sanger sequencing traces of the control and knockin hiPSC lines. The two different DNA templates used to introduce the intended mutation are depicted. Intended mutation is indicated in green.

(B–G) Molecular and functional analyses on 1-month-old hiPSC-derived cardiomyocytes obtained from three independent experiments.

(B) Gene expression levels of *DSP* normalized to *GUS*.

(C) Representative immunoblots for DSP.

(D) Quantification of DSP protein levels normalized to VIN.

(E) Representative immunoblots for DSC, DSG, JUP, and PKP2.

(F) Quantification of the desmosomal protein levels. Values normalized to VIN.

(G) APD measured at 50% and 90% (*KI. DSP*$^{WT/WT}$, n = 48 cell clusters; *KI. DSP*$^{p.Tyr1188His/WT}$, n = 81 cell clusters) of cardiomyocyte repolarization.

(legend continued on next page)

Together, we robustly identified impaired expression of cardiac ion-handling-related components as a consequence of the novel *DSP*$^{p.Tyr1188His}$ mutation.

PITX2 levels are increased in *DSP*$^{p.Tyr1188His/WT}$ cardiomyocytes and repress expression of structural and ion-handling genes

The observation that many ion channels, indispensable for proper cardiomyocyte function, were dysregulated in *Pa.* and *KI. DSP*$^{p.Tyr1188His/WT}$ cardiomyocytes prompted us to investigate potential upstream effectors. Martin and coworkers previously reported that the transcription factor PITX2 dictates a gene network in mouse postnatal atrial cardiomyocytes encompassing channel and calcium-handling genes as well as genes involved in stabilizing cell-cell junctions (Tao et al., 2014). The authors combined chromatin immunoprecipitation sequencing and transcriptomics on conditional *Pitx2* knockout mice, demonstrating that a loss of *Pitx2* in cardiomyocytes results in upregulation of *Dsp*, *Gja1*, and *Scn5a*, indicating that PITX2 acts as a repressor for these genes. In humans, mutations in genomic loci adjacent to or within *PITX2*, thereby affecting its expression, predisposes the heart to atrial arrhythmias (Chinchilla et al., 2011; Gudbjartsson et al., 2007; Herraiz-Martínez et al., 2021; Kaab et al., 2008; Ouwerkerk et al., 2020). Based on these findings, we hypothesized that dysregulation of PITX2 in *DSP*$^{p.Tyr1188His/WT}$ cardiomyocytes might contribute to the observed reduction of DSP, CX43, and NaV1.5. mRNA sequencing analyses and subsequent validation experiments revealed an induction of *PITX2* in *Pa.* and *KI. DSP*$^{p.Tyr1188His/WT}$ cardiomyocytes (Figure 6A), which was confirmed at the protein level (Figures 6B–6D). To see whether *PITX2* could evoke a similar response in an independent model, we used lentivirus to overexpress *PITX2* (lenti-PITX2) for 7 days in healthy hiPSC-derived control cardiomyocytes. *PITX2* levels were induced approximately 20-fold compared with the baseline condition (Figures S6A and S6C). The PITX2 targets *DSP*, *GJA1*, and *SCN5A* were all repressed in lenti-PITX2-treated cardiomyocytes (Figures S6B and S6C). On the protein level, we could confirm the induction of PITX2 and reduced levels of DSP and CX43 (Figures 6E and 6F). Functionally, we observed a significant prolongation of the APD at 90% of cardiomyocyte repolarization (Figure 6G). On the contrary, knockdown of *PITX2* gene expression in control cardiomyocytes induced the expression of *DSP*, *GJA1*, and *SCN5A* (Figures S6D–S6F), which was confirmed by immunoblotting for PITX2, DSP, and CX43 (Figures S6G and S6H). The APD was shortened in si-PITX2-treated cardiomyocytes at 50% and 90% of repolarization compared with control (Figure S6I). These results demonstrate that PITX2 is induced in *Pa.* and *KI. DSP*$^{p.Tyr1188His/WT}$ cardiomyocytes and that PITX2 represses genes important for cardiomyocyte function.

Suppression of *PITX2* in *Pa. DSP*$^{p.Tyr1188His/WT}$ cardiomyocytes restores expression of structural and ion-handling genes

After identifying PITX2 as potential upstream effector of *DSP*, GJA1, and *SCN5A*, we speculated that knockdown of *PITX2* in *Pa. DSP*$^{p.Tyr1188His/WT}$ cardiomyocytes could restore the observed phenotype. To investigate this, we treated 1-month-old mutant and isogenic control cardiomyocytes with scramble small interfering RNA (siRNA) or siRNA targeting *PITX2* for 72 h. *PITX2* levels were reduced by approximately 90% in both control and mutant cardiomyocytes, which led to a significant increase in *GJA1* expression in *Pa. DSP*$^{p.Tyr1188His/WT}$ cardiomyocytes (Figures S7A and S7B). On the protein level, we observed restoration of DSP, CX43, and NaV1.5 upon silencing of *PITX2* in mutant cardiomyocytes (Figures 7A and 7B). To explore if *PITX2* induction is unique to the novel *DSP*$^{p.Tyr1188His}$ mutation or whether it is a more general phenomenon for mutations in *DSP*, we assessed the levels of *PITX2* in hiPSC-derived cardiomyocytes bearing a known disease-causing mutation in *DSP* (*KI. DSP*$^{p.Arg1113X/WT}$). Compared with control cells, mutant cardiomyocytes display reduced levels of *DSP* (Figures 7C and S7C). Interestingly, this reduction is accompanied by elevated levels of *PITX2*, which was confirmed at the protein level (Figures 7C–7E). Altogether, knockdown of the repressor *PITX2* in *Pa. DSP*$^{p.Tyr1188His/WT}$ cardiomyocytes restores expression of genes important for cardiomyocyte function. Additionally, *KI. DSP*$^{p.Arg1113X/WT}$ cardiomyocytes show induced levels of PITX2, suggesting a more general mechanism.

DISCUSSION

Through the use of matching patient-derived (*Pa. DSP*$^{p.Tyr1188His/WT}$) and KI (*KI. DSP*$^{p.Tyr1188His/WT}$) hiPSC-derived cardiomyocytes, we demonstrated that this novel *DSP* mutation evokes molecular and functional changes that can be linked to ACM pathogenesis. This implies

Data are plotted as mean. The dots in (B), (D), (F), and (G) represent technical replicates, whereas the color of each dot indicates the experimental origin (3 independent experiments; 4–60 technical replicates). Significance has been assessed by a two-tailed unpaired 2Student's t test or two-tailed Mann-Whitney test when data were not normally distributed (*p < 0.05, ****p < 0.0001, ns, not significant).

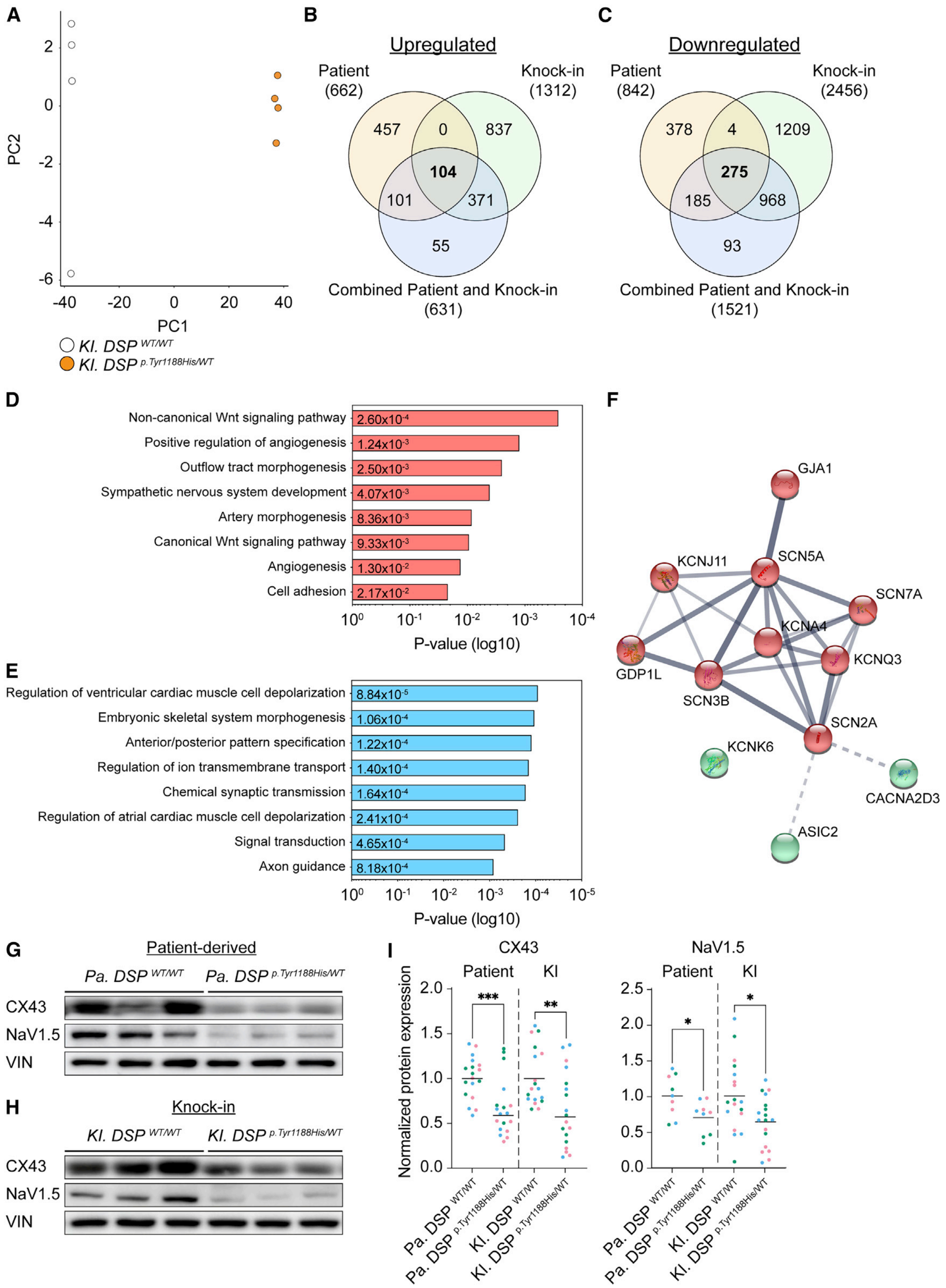

Figure 5. *Pa. DSP*$^{p.Tyr1188His/WT}$ and *KI. DSP*$^{p.Tyr1188His/WT}$ cardiomyocytes display impaired ion-handling

(A) PCA reveals different gene expression profiles of *KI. DSP*$^{WT/WT}$ and *KI. DSP*$^{p.Tyr1188His/WT}$ cardiomyocytes.

(B–F) Combined analyses of the *Pa. DSP*$^{p.Tyr1188His/WT}$ (patient) and *KI. DSP*$^{p.Tyr1188His/WT}$ (knockin) mRNA sequencing datasets.

(B and C) Venn diagram showing the overlap of the up- (B; fold change (log2) >1) and downregulated (C; fold change (log2) <−1) genes.

(legend continued on next page)

that this mutation is causal for the observed phenotype in the patient clinically diagnosed with ACM.

Molecular analyses of *DSP*$^{p.Tyr1188His/WT}$ hiPSC-derived cardiomyocytes revealed a reduction in desmosomal proteins, an observation previously reported in ventricular tissue obtained from patients with ACM (Asimaki et al., 2009). Interestingly, this pathomolecular characteristic seems to be a hallmark of ACM, as it is not evident in other forms of cardiac disease (Asimaki et al., 2009; Bueno-Beti and Asimaki, 2021). Besides reduced protein levels, we also observed lower mRNA levels for several components of the intercalated disc. This is in contrast to a recent study from our lab in which we only observed an effect on the protein level in an *in vitro* and *in vivo* KI model of an ACM-driving *PKP2* mutation, indicating that the *DSP*$^{p.Tyr1188His/WT}$ mutation evokes a different molecular response (H. Tsui, S.J.K., S.J.H., V. Meraviglia, W.B.H., S. Casini, P. van der Kraak, A.V., X. Yin, M. Mayr, A. Bossu, G. Marchal, J.M.-K., J. Eding, D. Versteeg, K. Bezstarosti, J.A.G., S. Klaasen, L. van Laake, J. Demmers, G. Kops, C. Mummery, T.A.B.V., C. Remme, M. Bellin, E.R., unpublished data). In support of this, a change in the transcriptomic landscape was demonstrated through dysregulation of genes associated with ion handling, Wnt signaling, and transcriptional activity. Of note, compared with control cardiomyocytes, *KI. DSP*$^{p.Tyr1188His/WT}$ cardiomyocytes showed reduced *DSP* mRNA levels, whereas *Pa. DSP*$^{p.Tyr1188His/WT}$ cardiomyocytes displayed similar expression levels. Moreover, we consistently observed higher protein levels for PITX2 in the KI line compared with the patient-derived line. Since *DSP* is one of the potential downstream targets of the repressor PITX2, it is conceivable that the repressive effect on *DSP* expression is augmented in *KI. DSP*$^{p.Tyr1188His/WT}$ cardiomyocytes compared with patient-derived cardiomyocytes (Tao et al., 2014). More experiments are required to pinpoint the exact underlying molecular mechanism.

We further showed that mutant *DSP*$^{p.Tyr1188His/WT}$ cardiomyocytes exhibit lower levels of the gap junction protein CX43 and NaV1.5, which in turn may be causal for the observed prolongation of the action potential. These observations are in line with previous studies that demonstrated diminished CX43 and NaV1.5 levels in explanted cardiac material isolated from patients diagnosed with ACM (Asimaki et al., 2009; Bueno-Beti and Asimaki, 2021; Jansen et al., 2012). In addition, studies revealed redistribution of CX43 to the long axis of cardiomyocytes upon desmosomal protein deficiency, which in turn may act as an arrhythmogenic substrate (Bueno-Beti and Asimaki, 2021; Oxford et al., 2007; Zhang et al., 2013). The observed reduction and redistribution of proteins crucial for electrical signal propagation in cardiomyocytes may be explained at two levels. Firstly, as the desmosomes are interconnected with adherens junctions, gap junctions, and ion channels, it is conceivable that desmosomal mutations do not only affect the integrity of desmosomes but also of the linked protein complexes (Vermij et al., 2017). Specifically, proper localization of CX43 and NaV1.5 to cell-cell junctions seem to be highly dependent on a functional desmosome (Cerrone et al., 2014; Gusev et al., 2020; Jansen et al., 2012; Lyon et al., 2014; Sato et al., 2009; Zhang et al., 2013). Secondly, influential transcriptional programs such as Hippo and Wnt signaling are partly regulated by proteins residing at the intercalated discs in cardiomyocytes (Chen et al., 2014; Conti et al., 2004; Garcia-Gras et al., 2006; Guo et al., 2020). Desmosomal instability may therefore affect these signaling cascades and in turn affect the expression of structural and ion-handling-related genes. For instance, CX43 (*GJA1*) is a target of canonical Wnt signaling (Ai et al., 2000).

Here, we uncovered that aberrant expression of PITX2 in mutant *DSP*$^{p.Tyr1188His/WT}$ cardiomyocytes contribute to the observed phenotype. Interestingly, mutations within *PITX2*, which physically interacts with FOXC1 and FOXC2, have previously been linked to the ocular conditions Axenfeld-Rieger syndrome and glaucoma (Acharya et al., 2011). In the heart, *PITX2* is predominantly expressed in the left atria and well known for its role in atrial fibrillation; however, it has also been detected in ventricular tissue (Chinchilla et al., 2018; Furtado et al., 2011; Ouwerkerk et al., 2020; Tao et al., 2016; Torrado et al., 2014). Moreover, it has been proposed that atrial disease can be a subentity of heart failure induced by ventricular abnormalities (Coats et al., 2021). Mikhailov and coworkers demonstrated that *PITX2* expression is reactivated in the ventricular failing myocardium of patients experiencing systolic heart failure (Torrado et al., 2014). Likewise, *PITX2* is induced after

(D and E) Gene Ontology analyses on the up- (D) and downregulated (E) genes overlapping between the two datasets.
(F) STRING: functional protein association network for the gene ontology terms "regulation of ventricular cardiac muscle cell depolarization" and "regulation of ion transmembrane transport." Clustered based on kmeans. The thickness of each line indicates the level of confidence (data supported) and the colors to which cluster each component belongs.
(G–H) Representative immunoblots for CX43 and NaV1.5 in *Pa. DSP*$^{p.Tyr1188His/WT}$ (G) and *KI. DSP*$^{p.Tyr1188His/WT}$ (H) and isogenic control cardiomyocytes.
(I) Quantification of CX43 and NaV1.5 protein levels. Values normalized to VIN. Data are plotted as mean.
The dots in (I) represent technical replicates, whereas the color of each dot indicates the experimental origin (3 independent experiments; 4–6 technical replicates). Significance has been assessed by a two-tailed unpaired Student's t test (*$p < 0.05$, **$p < 0.01$, ***$p < 0.001$).

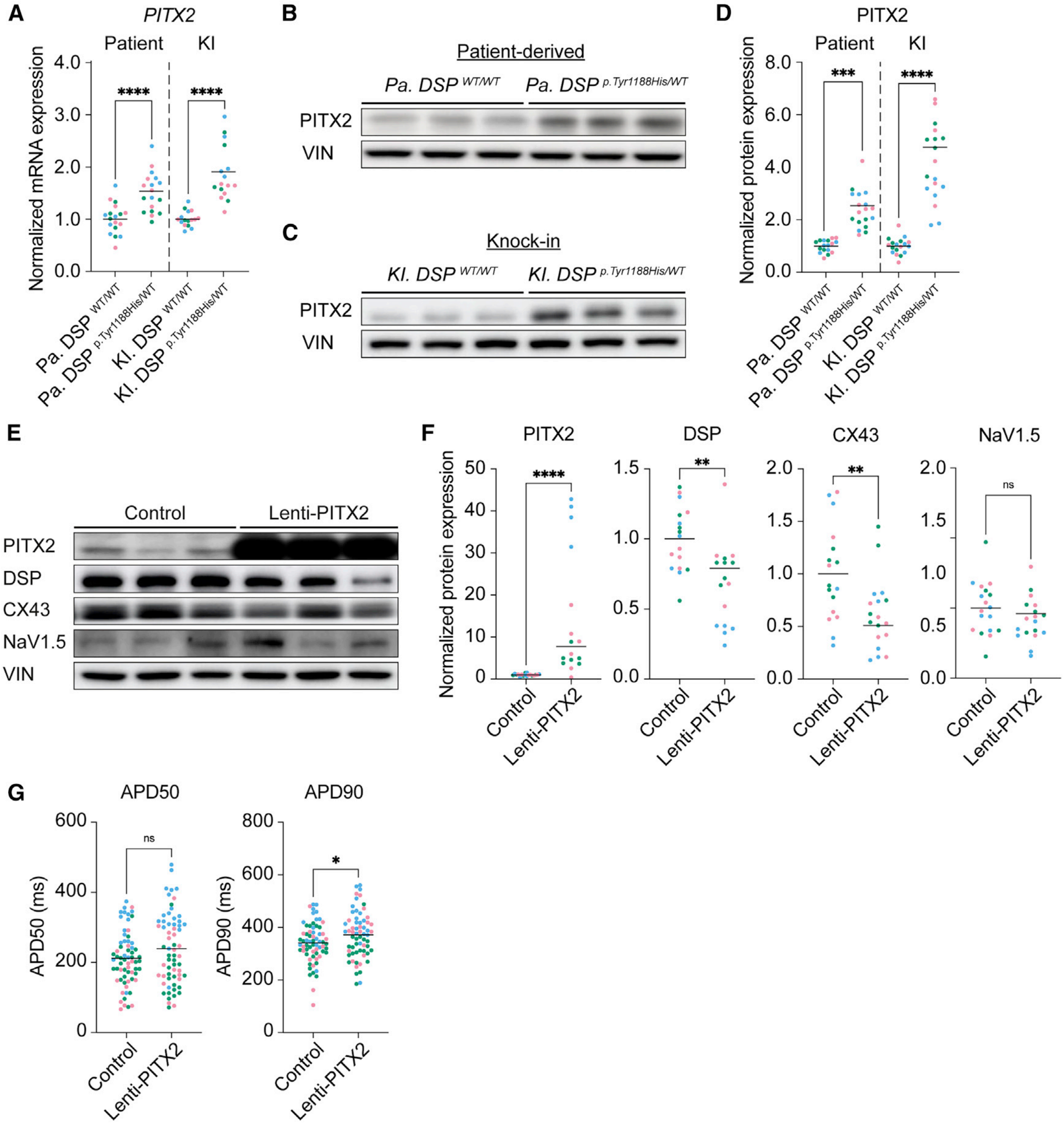

Figure 6. Paired-like homeodomain 2 levels are increased in mutant cardiomyocytes and repress expression of DSP, CX43, and NaV1.5

(A–D) Molecular analyses on 1-month-old *Pa. DSP*$^{p.Tyr1188His/WT}$, *KI. DSP*$^{p.Tyr1188His/WT}$, and isogenic control cardiomyocytes.
(A) Gene expression levels for *PITX2* normalized to *GUS*.
(B and C) Representative immunoblots for PITX2 in *Pa. DSP*$^{p.Tyr1188His/WT}$ (B) and *KI. DSP*$^{p.Tyr1188His/WT}$ (C) cardiomyocytes.
(D) Quantification of PITX2 protein levels normalized to VIN.
(E–G) Molecular and functional analyses on 1-month-old control hiPSC-derived cardiomyocytes treated with either empty viral particles or particles encoding for *PITX2* (lenti-PITX2).
(E) Representative immunoblots for PITX2, DSP, CX43, and NaV1.5. (F) Protein levels of PITX2, DSP, CX43, and NaV1.5 normalized to VIN.
(G) APD measured at 50% and 90% (control, n = 66 cell clusters; lenti-PITX2, n = 64 cell clusters) of cardiomyocyte repolarization.
Data are plotted as mean. The dots in (A), (D), (F), and (G) represent technical replicates, whereas the color of each dot indicates the experimental origin (3 independent experiments; 4–29 technical replicates). Significance has been assessed by a two-tailed unpaired Student's t test or two-tailed Mann-Whitney test when data were not normally distributed (*p < 0.05, **p < 0.01, ***p < 0.001, ****p < 0.0001, ns, not significant). KI, knockin; Pa., patient.

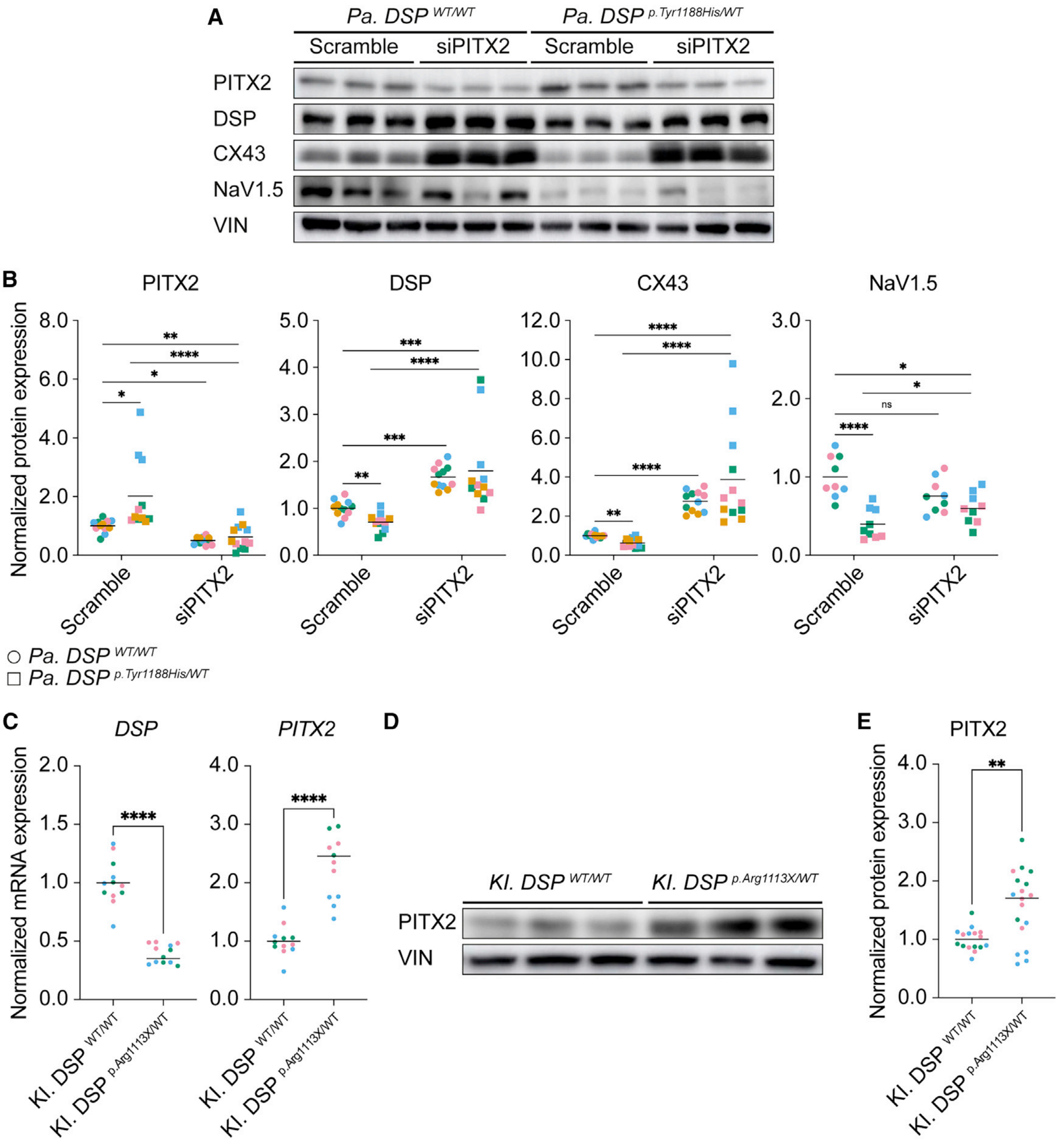

Figure 7. RNAi-mediated knockdown of paired-like homeodomain 2 in *Pa. DSP*$^{p.Tyr1188His/WT}$ cardiomyocytes rescues the expression of structural and ion-related units

(A and B) Molecular analyses on 1-month-old *Pa. DSP*$^{WT/WT}$ and *Pa. DSP*$^{p.Tyr1188His/WT}$ cardiomyocytes treated with scramble siRNA or siRNA targeting *PITX2* for 72 h. Data are obtained from 3–4 independent experiments.

(A) Representative immunoblots for PITX2, DSP, CX43, NaV1.5, and VIN.

(B) Quantification for PITX2, DSP, CX43, and NaV1.5 normalized to VIN.

(C–E) Molecular analyses on 1-month-old *KI. DSP*$^{WT/WT}$ and *KI. DSP*$^{p.Arg1113X/WT}$ cardiomyocytes. Data are obtained from three independent experiments.

(C) Gene expression levels for *DSP* and *PITX2* normalized to *GUS*.

(D) Representative immunoblots for PITX2 and VIN.

(E) Quantification of PITX2 protein levels normalized to VIN.

Data are plotted as mean. The dots in (B), (C), and (E) represent technical replicates, whereas the color of each dot indicates the experimental origin (3–4 independent experiments; 3–6 technical replicates). For (B), significance has been assessed on log-transformed data using an ordinary two-way ANOVA followed by a Tukey's multiple comparisons test (single pooled variance; alpha = 0.05). For (C) and (E), a two-tailed unpaired Student's t test was applied (*$p < 0.05$, **$p < 0.01$, ***$p < 0.001$, ****$p < 0.0001$, ns, not significant).

myocardial infarction in Hippo-deficient mouse ventricles, subsequently activating expression of genes associated with the electron transport chain and reactive oxygen species scavengers (Tao et al., 2016). Through modulation of *PITX2* expression levels in mutant *Pa. DSP*$^{p.Tyr1188His/WT}$ cardiomyocytes, we demonstrate reactivation of genes important for ion handling and signal propagation, including *SCN5A* and *GJA1*. Previously, PITX2 has been linked to Hippo and Wnt signaling, cascades reported to be frequently dysregulated in the setting of ACM (Basu and Roy, 2013; Briata et al., 2003; Garcia-Gras et al., 2006; Tao et al., 2016). We did notice enrichment for terms related to (non)-canonical Wnt signaling in our transcriptome datasets, which might provide a link between mutations in *DSP* and *PITX2* induction. Together, these findings imply that *PITX2* may play a role in the malfunctioning ventricular myocardium. Further research is required to unravel the exact mechanisms leading to *PITX2* induction in cardiomyocytes bearing mutations in *DSP*.

In conclusion, our results reveal that the novel *DSP*$^{p.Tyr1188His}$ mutation evokes a pathological response in cardiomyocytes. We observed reduced desmosomal protein levels, which were accompanied by a reduction in CX43 and NaV1.5. Functionally, mutant cardiomyocytes displayed a prolonged APD that could be linked to induced levels of the repressor PITX2. Indeed, knockdown of *PITX2* in patient-derived cardiomyocytes could alleviate the observed repressive effects. Even though the mutation-induced molecular changes are well defined in this study, the electrophysiological properties are not because of technical limitations inherent of the followed methodology. Follow-up studies should implement patch-clamp experiments to further facilitate our understanding of the electrical alterations caused by the mutation. Together, our data underscore the advantages of combining patient and KI hiPSC-derived cardiomyocytes, bearing mutations in the endogenous locus, to identify a potential novel therapeutic target for the treatment of ACM.

EXPERIMENTAL PROCEDURES

Resource availability

Corresponding author

For further information, please contact Eva van Rooij (e.vanrooij@hubrecht.eu).

Materials availability

Materials and additional details can be made available from the corresponding author upon reasonable request.

Data and code availability

The mRNA sequencing datasets have been deposited with the Gene Expression Omnibus repository under accession numbers GEO: GSE208213 and GSE208212 for the patient and KI lines, respectively.

Co-immunoprecipitation

HEK293T cells grown on a 145 cm^2 dish until 60%–70% confluency were transfected with the indicated plasmids using polyethylenimine. After 48 h, protein was isolated using a mild lysis buffer. Magnetic beads coated with monoclonal anti-FLAG M2 were used to pull down protein complexes containing DSP-WT:FLAG molecules. Samples were analyzed on a 7% SDS-PAGE gel using the antibodies listed in Table S4.

Cardiomyocyte cultures

The genomic integrity and pluripotency status of genetically modified hiPSCs was assessed by means of targeted sequencing, immunofluorescence, and karyo sequencing. To initiate directed differentiation toward cardiomyocytes, hiPSCs were cultured on Geltrex-coated plates in Essential 8 Medium until 80%–90% confluency, Next, medium was refreshed with RPMI-1640-Medium-GlutaMAX-Supplement-HEPES supplemented with human recombinant albumin, L-Ascorbic Acid 2-Phosphate, and CHIR99021 (cardio differentiation medium with CHIR). After 48 h, medium was refreshed with cardio differentiation medium with IWP2. After 48 and 96 h, cells were refreshed with plain cardio differentiation medium. From day 8 onward, cells were kept in RPMI-1640-Medium-GlutaMAX-Supplement-HEPES supplemented with B-27-Supplement-serum free. hiPSC-derived cardiomyocyte cultures were subsequently analyzed by fluorescence-activated cell sorting (FACS; BD Biosciences, FACSCalibur) for the percentage of cardiomyocytes (positive for cardiac Troponin T).

Molecular assays

hiPSC-derived cardiomyocytes were seeded at a density of 100,000 cells/cm^2 for downstream applications. We analyzed the RNA, protein, and functional properties of 1-month-old cardiomyocytes either kept under baseline conditions, transfected with control siRNA or an siRNA targeting *PITX2*, or infected with control virus or lenti-PITX2. For the functional experiments, cells were grown in clusters and treated with FluoVolt and Powerload for 15 min at 37°C. During measurements, cells were immersed in a solution containing, in mM, NaCl (130), KCl (4), CaCl2 (1.8), MgCl2 (1.2), NaHCO3 (18), HEPES (10), and glucose (10) (pH 7.4). A custom-build microscope with a 10× objective was used for the recordings. APDs were corrected for the beating rate using an adjustment of the Fredericia formula: APDcorrected = APD/($\sqrt[3]{}$(60/BPM)).

mRNA sequencing

mRNA-sequencing was performed on 1-month-old hiPSC-derived cardiomyocytes. RNA libraries were prepared with the TruSeq Stranded mRNA polyA kit (Illumina) according to the manufacturer's protocol. Strand-specific single-end 75 bp reads were generated on an Illumina NextSeq 500 system. Reads were checked for their quality using FastQC and aligned against the human genome (assembly GRCh37) using STAR (STAR_2.4.2a). Differential expression was calculated using DESeq2 v.1.2 with pooled dispersion estimates. Differentially expressed genes were further analyzed for functional enrichment using the STRING v.11.5 database. No changes were made to the default settings. *Homo sapiens* served as background. Only the molecular function (GO-MF), biological

process (GO-BP), cellular component (GO-CC), and KEGG data sources were considered for enrichment analysis.

Human material

The study fulfilled the Dutch criteria of the code of proper use of human tissue. Written informed consent was obtained for the generation and use of the patient-derived hiPSCs.

Statistical analysis

The number of samples (n) used in each experiment is indicated in the legend of each figure. Data are presented as mean. Statistical analyses were performed using PRISM (GraphPad Software v. 9). Outliers were identified using the ROUT method (Q = 5%) and were removed if present. For comparison of two groups, data were tested for normality using the Kolmogorov-Smirnov test with Dallal-Wilkinson-Lillie for p value method (alpha = 0.05). Significance has been assessed by a two-tailed unpaired Student's t test or two-tailed Mann-Whitney test when data were not normally distributed. For comparisons of four groups with two variables (genotype and treatment), data were tested for homoscedasticity (Spearman's rank correlation test; alpha = 0.05) and Gaussian distribution (Kolmogorov-Smirnov test; alpha = 0.05). In case these requirements were not met, a log transformation was applied. Data were then tested for significance using an ordinary two-way ANOVA, followed by a Tukey's multiple comparisons test (single pooled variance; alpha = 0.05). Correlation and significance between proteins have been assessed by non-parametric Spearman correlation (two-tailed; alpha = 0.05). Differences were considered statistically significant when $p < 0.05$. Asterisks indicate statistical significance (*$p < 0.05$, **$p < 0.01$, ***$p < 0.001$, ****$p < 0.0001$).

SUPPLEMENTAL INFORMATION

Supplemental information can be found online at https://doi.org/10.1016/j.stemcr.2023.01.015.

AUTHOR CONTRIBUTIONS

S.J.v.K. designed and performed all experiments related to hiPSC targeting using CRISPR-Cas9. S.J.v.K., C.J.B., and E.v.R. designed all molecular experiments related to phenotyping of the hiPSC-derived cardiomyocytes. S.J.v.K., S.J.H., E.K., E.W.S., and J.M.-K. maintained the hiPSC lines and performed molecular experiments on hiPSC-derived cardiomyocytes. S.J.v.K. and C.J.B. performed mRNA sequencing analyses. W.B.v.H., B.G., T.P.d.B., and T.A.B.v.V. designed and performed the electrophysiology experiments. S.J.v.K., I.P., and H.d.R. designed and performed the experiments regarding co-immunoprecipitation and cloning. L.W.v.L. and J.A.G. provided the human skin biopsies and clinical data. S.J.v.K. and E.v.R. planned all experiments, performed data analyses, and wrote the manuscript.

ACKNOWLEDGMENTS

We thank V. Sigurdsson and the department of dermatology, UMC Utrecht, for help with skin biopsies. We thank Farhad A. Moqadam and Ana Rita Leitoguinho for the design of the gRNAs. We also thank Sjoerd J. Klaasen and Geert J.P.L. Kops for their help with the karyo sequencing procedure. This work was supported by funds from the Dutch Cardiovascular Alliance with the support of the Dutch Heart Foundation, DCVA2017-18 ARENA-PRIME (E.v.R.) and CVON2018-30 PREDICT2 (W.B.v.H. and T.A.B.v.V.); the Vici grant from the Dutch Research Council (NWO), project 09150181910020 (E.v.R.); and by the Fondation Leducq Transatlantic Network of Excellence, 17CVD02 (E.v.R.).

CONFLICTS OF INTEREST

The authors declare no competing interests.

Received: August 29, 2022
Revised: January 30, 2023
Accepted: January 30, 2023
Published: March 2, 2023

REFERENCES

Acharya, M., Huang, L., Fleisch, V.C., Allison, W.T., and Walter, M.A. (2011). A complex regulatory network of transcription factors critical for ocular development and disease. Hum. Mol. Genet. *20*, 1610–1624.

Ai, Z., Fischer, A., Spray, D.C., Brown, A.M.C., and Fishman, G.I. (2000). Wnt-1 regulation of connexin43 in cardiac myocytes. J. Clin. Invest. *105*, 161–171.

Asimaki, A., Tandri, H., Huang, H., Halushka, M.K., Gautam, S., Basso, C., Thiene, G., Tsatsopoulou, A., Protonotarios, N., McKenna, W.J., et al. (2009). A new diagnostic test for arrhythmogenic right ventricular cardiomyopathy. N. Engl. J. Med. *360*, 1075–1084.

Basso, C., Pilichou, K., Bauce, B., Corrado, D., and Thiene, G. (2018). Diagnostic criteria, genetics, and molecular basis of arrhythmogenic cardiomyopathy. Heart Fail. Clin. *14*, 201–213.

Basu, M., and Roy, S.S. (2013). Wnt/β-catenin pathway is regulated by PITX2 homeodomain protein and thus contributes to the proliferation of human ovarian adenocarcinoma cell, SKOV-3. J. Biol. Chem. *288*, 4355–4367.

Briata, P., Ilengo, C., Corte, G., Moroni, C., Rosenfeld, M.G., Chen, C.Y., and Gherzi, R. (2003). The Wnt/β-catenin→Pitx2 pathway controls the turnover of Pitx2 and other unstable mRNAs. Mol. Cell *12*, 1201–1211.

Bueno-Beti, C., and Asimaki, A. (2021). Histopathological features and protein markers of arrhythmogenic cardiomyopathy. Front. Cardiovasc. Med. *8*, 746321.

Cerrone, M., Lin, X., Zhang, M., Agullo-Pascual, E., Pfenniger, A., Chkourko Gusky, H., Novelli, V., Kim, C., Tirasawadichai, T., Judge, D.P., et al. (2014). Missense mutations in plakophilin-2 cause sodium current deficit and associate with a brugada syndrome phenotype. Circulation *129*, 1092–1103.

Chen, S.N., Gurha, P., Lombardi, R., Ruggiero, A., Willerson, J.T., and Marian, A.J. (2014). The hippo pathway is activated and is a causal mechanism for adipogenesis in arrhythmogenic cardiomyopathy. Circ. Res. *114*, 454–468.

Chinchilla, A., Daimi, H., Lozano-Velasco, E., Dominguez, J.N., Caballero, R., Delpón, E., Tamargo, J., Cinca, J., Hove-Madsen, L., Aranega, A.E., and Franco, D. (2011). PITX2 insufficiency leads to

atrial electrical and structural remodeling linked to arrhythmogenesis. Circ. Cardiovasc. Genet. *4*, 269–279.

Chinchilla, A., Esteban, F.J., Lozano-Velasco, E., Hernandez-Torres, F., Dominguez, J.N., Aránega, A.E., and Franco, D. (2018). Ventricular chamber-specific pitx2 insufficency leads to cardiac hypertrophy and arrhythmias. Preprint at bioRxiv. https://doi.org/10.1101/253062.

Coats, A.J.S., Heymans, S., Farmakis, D., Anker, S.D., Backs, J., Bauersachs, J., Boer, R.A.D., Jelena, Č., Cleland, J.G.F., Dobrev, D., et al. (2021). Atrial disease and heart failure : the common soil hypothesis proposed by the Heart Failure Association of the European Society of Cardiology. Eur. Heart J. *43*, 1635.

Conti, M.A., Even-Ram, S., Liu, C., Yamada, K.M., and Adelstein, R.S. (2004). Defects in cell adhesion and the visceral endoderm following ablation of nonmuscle myosin heavy chain II-A in mice. J. Biol. Chem. *279*, 41263–41266.

Furtado, M.B., Biben, C., Shiratori, H., Hamada, H., and Harvey, R.P. (2011). Characterization of Pitx2c expression in the mouse heart using a reporter transgene. Dev. Dyn. *240*, 195–203.

Gao, S., Puthenvedu, D., Lombardi, R., and Chen, S.N. (2020). Established and emerging mechanisms in the pathogenesis of arrhythmogenic cardiomyopathy: a multifaceted disease. Int. J. Mol. Sci. *21*, 6320.

Garcia-Gras, E., Lombardi, R., Giocondo, M.J., Willerson, J.T., Schneider, M.D., Khoury, D.S., and Marian, A.J. (2006). Suppression of canonical Wnt/β-catenin signaling by nuclear plakoglobin recapitulates phenotype of arrhythmogenic right ventricular cardiomyopathy. J. Clin. Invest. *116*, 2012–2021.

Gomes, J., Finlay, M., Ahmed, A.K., Ciaccio, E.J., Asimaki, A., Saffitz, J.E., Quarta, G., Nobles, M., Syrris, P., Chaubey, S., et al. (2012). Electrophysiological abnormalities precede overt structural changes in arrhythmogenic right ventricular cardiomyopathy due to mutations in desmoplakin-A combined murine and human study. Eur. Heart J. *33*, 1942–1953.

Green, K.J., Parry, D.A., Steinert, P.M., Virata, M.L., Wagner, R.M., Angst, B.D., and Nilles, L.A. (1990). Structure of the human desmoplakins. Implications for function in the desmosomal plaque. J. Biol. Chem. *265*, 11406–11407.

Gudbjartsson, D.F., Arnar, D.O., Helgadottir, A., Gretarsdottir, S., Holm, H., Sigurdsson, A., Jonasdottir, A., Baker, A., Thorleifsson, G., Kristjansson, K., et al. (2007). Variants conferring risk of atrial fibrillation on chromosome 4q25. Nature *448*, 353–357.

Guo, H., Lu, Y.W., Lin, Z., Huang, Z.P., Liu, J., Wang, Y., Seok, H.Y., Hu, X., Ma, Q., Li, K., et al. (2020). Intercalated disc protein Xinβ is required for Hippo-YAP signaling in the heart. Nat. Commun. *11*, 4666.

Gusev, K., Khudiakov, A., Zaytseva, A., Perepelina, K., Makeenok, S., Kaznacheyeva, E., and Kostareva, A. (2020). Impact of the DSP-H1684R genetic variant on ion channels activity in iPSC-derived cardiomyocytes. Cell. Physiol. Biochem. *54*, 696–706.

Herraiz-Martínez, A., Tarifa, C., Lozano-Velasco, E., Jiménez-Sábado, V., Casabella, S., Hernández-Torres, F., Daimi, H., Vázquez Ruiz de Castroviejo, E., Delpón, E., Caballero, R., et al. (2021). Novel PITX2 homeodomain-contained mutations from ATRIAL fibrillation patients deteriorate calcium homeostasis. Hearts *2*, 251–271.

Hoorntje, E.T., Te Rijdt, W.P., James, C.A., Pilichou, K., Basso, C., Judge, D.P., Bezzina, C.R., and Van Tintelen, J.P. (2017). Arrhythmogenic cardiomyopathy: pathology, genetics, and concepts in pathogenesis. Cardiovasc. Res. *113*, 1521–1531.

Jansen, J.A., Noorman, M., Musa, H., Stein, M., De Jong, S., Van Der Nagel, R., Hund, T.J., Mohler, P.J., Vos, M.A., Van Veen, T.A., et al. (2012). Reduced heterogeneous expression of Cx43 results in decreased Nav1.5 expression and reduced sodium current that accounts for arrhythmia vulnerability in conditional Cx43 knockout mice. Heart Rhythm *9*, 600–607.

Kaab, S., Darbar, D., van Noord, C., Dupuis, J., Pfeufer, A., Newton-Cheh, C., Schnabel, R., Makino, S., Sinner, M.F., Kannankeril, P.J., et al. (2008). Large scale replication and meta-analysis of variants on chromosome 4q25 associated with atrial fibrillation. Eur. Heart J. *30*, 813–819.

Lyon, R.C., Mezzano, V., Wright, A.T., Pfeiffer, E., Chuang, J., Banares, K., Castaneda, A., Ouyang, K., Cui, L., Contu, R., et al. (2014). Connexin defects underlie arrhythmogenic right ventricular cardiomyopathy in a novel mouse model. Hum. Mol. Genet. *23*, 1134–1150.

Marcus, F.I., Fontaine, G.H., Guiraudon, G., Frank, R., Laurenceau, J.L., Malergue, C., and Grosgogeat, Y. (1982). Right ventricular dysplasia: a report of 24 adult cases. Circulation *65*, 384–398.

Martherus, R., Jain, R., Takagi, K., Mendsaikhan, U., Turdi, S., Osinska, H., James, J.F., Kramer, K., Purevjav, E., and Towbin, J.A. (2016). Accelerated cardiac remodeling in desmoplakin transgenic mice in response to endurance exercise is associated with perturbed wnt/β-catenin signaling. Am. J. Physiol. Heart Circ. Physiol. *310*, H174–H187.

O'Keefe, E.J., Erickson, H.P., and Bennett, V. (1989). Desmoplakin I and desmoplakin II. Purification and characterization. J. Biol. Chem. *264*, 8310–8318.

Ouwerkerk, A.F.V., Hall, A.W., Kadow, Z.A., Lazarevic, S., Reyat, J.S., Tucker, N.R., Nadadur, R.D., Bosada, F.M., Bianchi, V., Ellinor, P.T., et al. (2020). Epigenetic and transcriptional networks underlying atrial fibrillation. Circ. Res. *127*, 34–50.

Oxford, E.M., Musa, H., Maass, K., Coombs, W., Taffet, S.M., and Delmar, M. (2007). Connexin43 remodeling caused by inhibition of plakophilin-2 expression in cardiac cells. Circ. Res. *101*, 703–711.

Sato, P.Y., Musa, H., Coombs, W., Guerrero-Serna, G., Patiño, G.A., Taffet, S.M., Isom, L.L., and Delmar, M. (2009). Loss of plakophilin-2 expression leads to decreased sodium current and slower conduction velocity in cultured cardiac myocytes. Circ. Res. *105*, 523–526.

Tao, G., Kahr, P.C., Morikawa, Y., Zhang, M., Rahmani, M., Heallen, T.R., Li, L., Sun, Z., Olson, E.N., Amendt, B.A., et al. (2016). Pitx2 promotes heart repair by activating the antioxidant response after cardiac injury. Nature *534*, 119–123.

Tao, Y., Zhang, M., Li, L., Bai, Y., Zhou, Y., Moon, A.M., Kaminski, H.J., and Martin, J.F. (2014). Pitx2, an atrial fibrillation predisposition gene, directly regulates ion transport and intercalated disc genes. Circ. Cardiovasc. Genet. *7*, 23–32.

Torrado, M., Franco, D., Hernández-Torres, F., Crespo-Leiro, M.G., Iglesias-Gil, C., Castro-Beiras, A., and Mikhailov, A.T. (2014). Pitx2c is reactivated in the failing myocardium and stimulates Myf5 expression in cultured cardiomyocytes. PLoS One *9*, e90561.

Veeraraghavan, R., and Gourdie, R.G. (2016). STORM-based quantitative assessment of sodium channel localization relative to junctional proteins within the cardiac intercalated disk. Microsc. Microanal. *22*, 1032–1033.

Vermij, S.H., Abriel, H., and van Veen, T.A.B. (2017). Refining the molecular organization of the cardiac intercalated disc. Cardiovasc. Res. *113*, 259–275.

Wang, W., Murray, B., Tichnell, C., Gilotra, N.A., Zimmerman, S.L., Gasperetti, A., Scheel, P., Tandri, H., Calkins, H., and James, C.A. (2022). Clinical characteristics and risk stratification of desmoplakin cardiomyopathy. Europace *24*, 268–277.

Yang, Z., Bowles, N.E., Scherer, S.E., Taylor, M.D., Kearney, D.L., Ge, S., Nadvoretskiy, V.V., DeFreitas, G., Carabello, B., Brandon, L.I., et al. (2006). Desmosomal dysfunction due to mutations in desmoplakin causes arrhythmogenic right ventricular dysplasia/cardiomyopathy. Circ. Res. *99*, 646–655.

Zhang, Q., Deng, C., Rao, F., Modi, R.M., Zhu, J., Liu, X., Mai, L., Tan, H., Yu, X., Lin, Q., et al. (2013). Silencing of desmoplakin decreases connexin43/Nav1.5 expression and sodium current in HL-1 cardiomyocytes. Mol. Med. Rep. *8*, 780–786.

Stem Cell Reports, Volume *18*

Supplemental Information

PITX2 induction leads to impaired cardiomyocyte function in arrhythmogenic cardiomyopathy

Sebastiaan J. van Kampen, Su Ji Han, Willem B. van Ham, Eirini Kyriakopoulou, Elizabeth W. Stouthart, Birgit Goversen, Jantine Monshouwer-Kloots, Ilaria Perini, Hesther de Ruiter, Petra van der Kraak, Aryan Vink, Linda W. van Laake, Judith A. Groeneweg, Teun P. de Boer, Hoyee Tsui, Cornelis J. Boogerd, Toon A.B. van Veen, and Eva van Rooij

Supplemental Figures

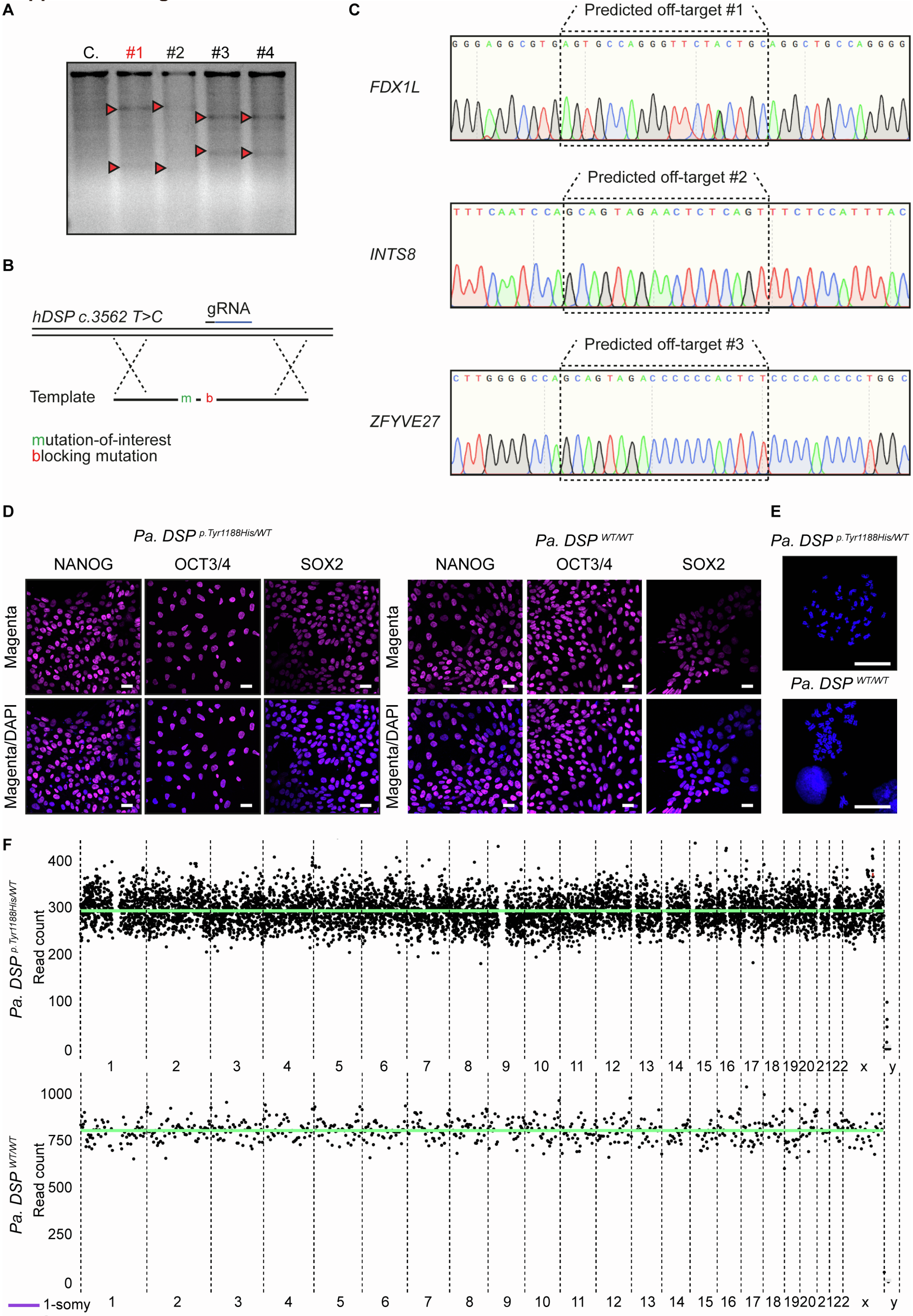

Figure S1. CRISPR/Cas9 targeting strategy and hiPSC characteristics of the *Pa. DSP* $^{p.Tyr1188His/WT}$ and isogenic control lines. Related to Figure 2.

(A) T7-endonuclease assay. gRNA #1, indicated in red, was selected for subsequent targeting. (B) Scheme depicting the CRISPR/Cas9 strategy used to correct *Pa. DSP* $^{p.Tyr1188His/WT}$ hiPSCs. Template length: 127 bp. (C) Representative Sanger sequencing traces for the top three off-target sites for gRNA #1. Dashed box indicates the potential binding site of gRNA #1 within *FDX1L*, *INTS8* and *ZFYVE27*. (D-F) Molecular analyses performed on the *Pa. DSP* $^{p.Tyr1188His/WT}$ and *Pa. DSP* $^{WT/WT}$ hiPSC lines. (D) Representative immunofluorescence images for NANOG, OCT3/4, SOX2 (all in magenta) and DAPI (blue). Scalebar: 20 μm. (E) Representative immunofluorescence images showing the correct number of chromosomes (DAPI in blue). Scalebar: 10 μm. (F) Karyo-sequencing profiles.

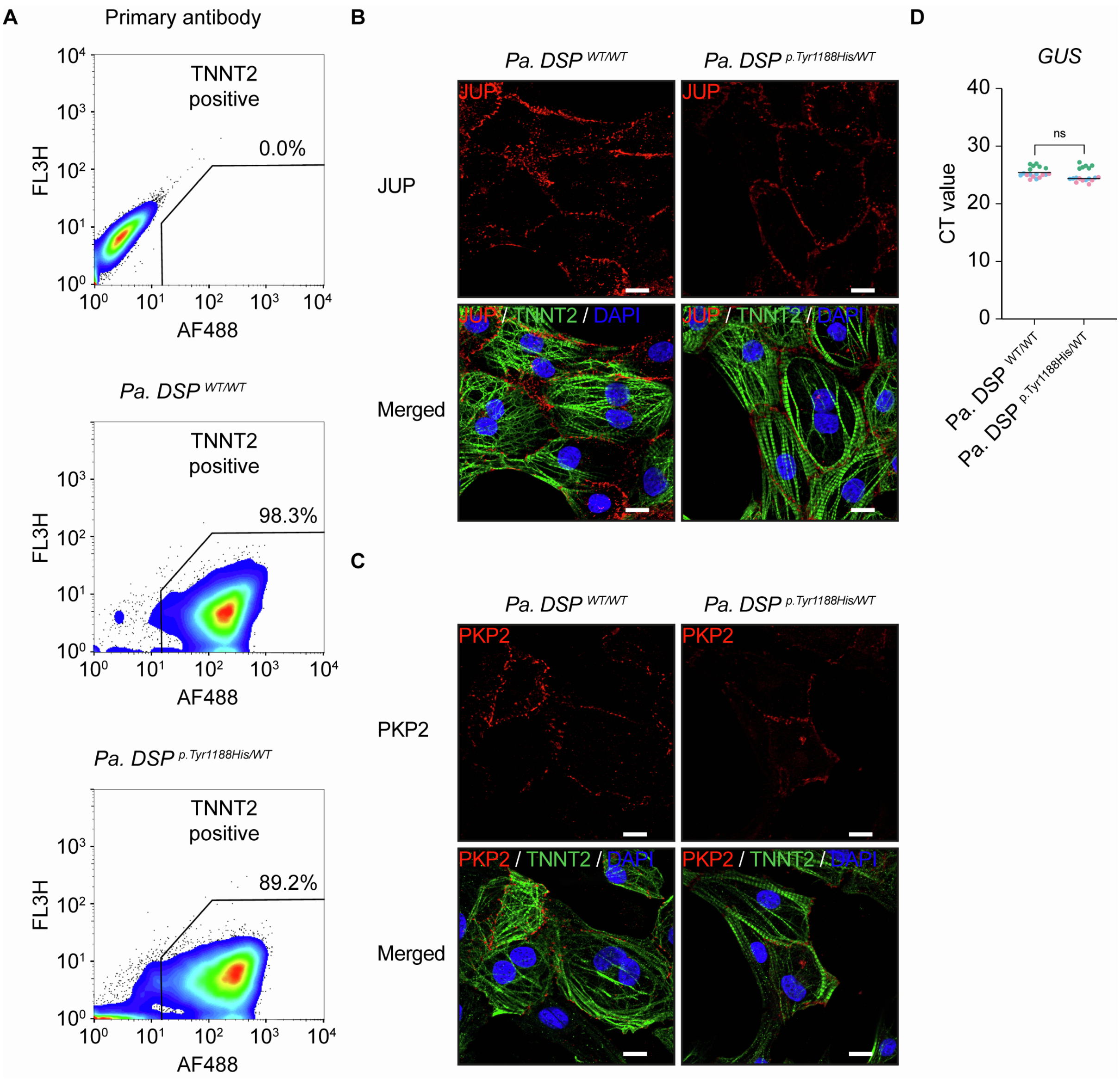

Figure S2. Differentiation and molecular characteristics of *Pa. DSP* $^{p.Tyr1188His/WT}$ and isogenic control cardiomyocytes. Related to Figure 2.

(A) Representative FACS plots showing the percentage of cardiomyocyte purity. Cells were stained with cardiac Troponin T (TNNT2, in green; AF488). (B-C) Representative immunofluorescence images for JUP (B) and PKP2 (C) in one-month-old wildtype and mutant cardiomyocytes. JUP and PKP2 in red; cardiac troponin T (TNNT2) in green; DAPI in blue. Scalebar: 10 µm. (D) Cycle threshold (CT) levels for *GUS* in one-month-old *Pa. DSP* $^{WT/WT}$ and *Pa. DSP* $^{p.Tyr1188His/WT}$ cardiomyocytes. Data plotted as mean. The dots in (D) represent technical replicates, whereas the color of each dot indicates the experimental origin (3 independent experiments; 6 technical replicates). Significance has been assessed by a two-tailed unpaired Student's t-test or two-tailed Mann-Whitney test when data were not normally distributed (ns: not significant).

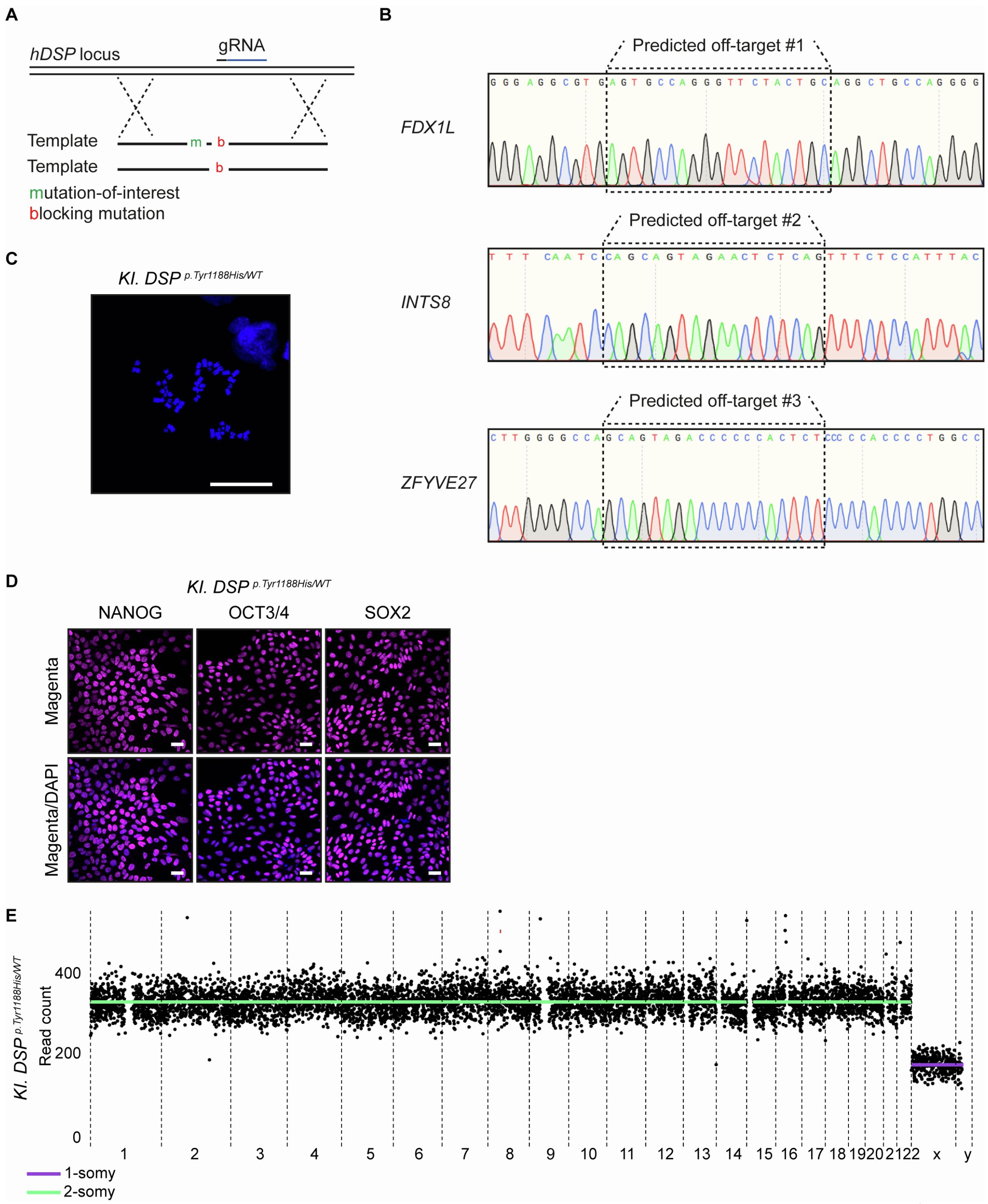

Figure S3. CRISPR/Cas9 targeting strategy and hiPSC characteristics of the *KI. DSP* $^{WT/WT}$ and *KI. DSP* $^{p.Tyr1188His/WT}$ lines. Related to Figure 4.

(A) Cartoon depicting the modified CRISPR/Cas9 strategy used to introduce the *DSP* $^{p.Tyr1188His}$ mutation in control hiPSCs. One single-stranded DNA template contains a blocking and the intended mutation, whereas the second template only contains the blocking mutation (Template length: 127 bp). (B-E) Analyses performed on the *KI. DSP* $^{p.Tyr1188His/WT}$ clone. (B) Representative Sanger sequencing traces for the top three off-target sites. Dashed box indicates the potential binding site of the gRNA within *FDX1L*, *INTS8* and *ZFYVE27*. (C) Representative immunofluorescence image showing the number of chromosomes (DAPI in blue). Scalebar: 10 μm. (D) Representative immunofluorescence images for NANOG, OCT3/4, SOX2 (all in magenta) and DAPI (blue). Scalebar: 20 μm. (E) Karyo-sequencing profile of the *KI. DSP* $^{p.Tyr1188His/WT}$ clone.

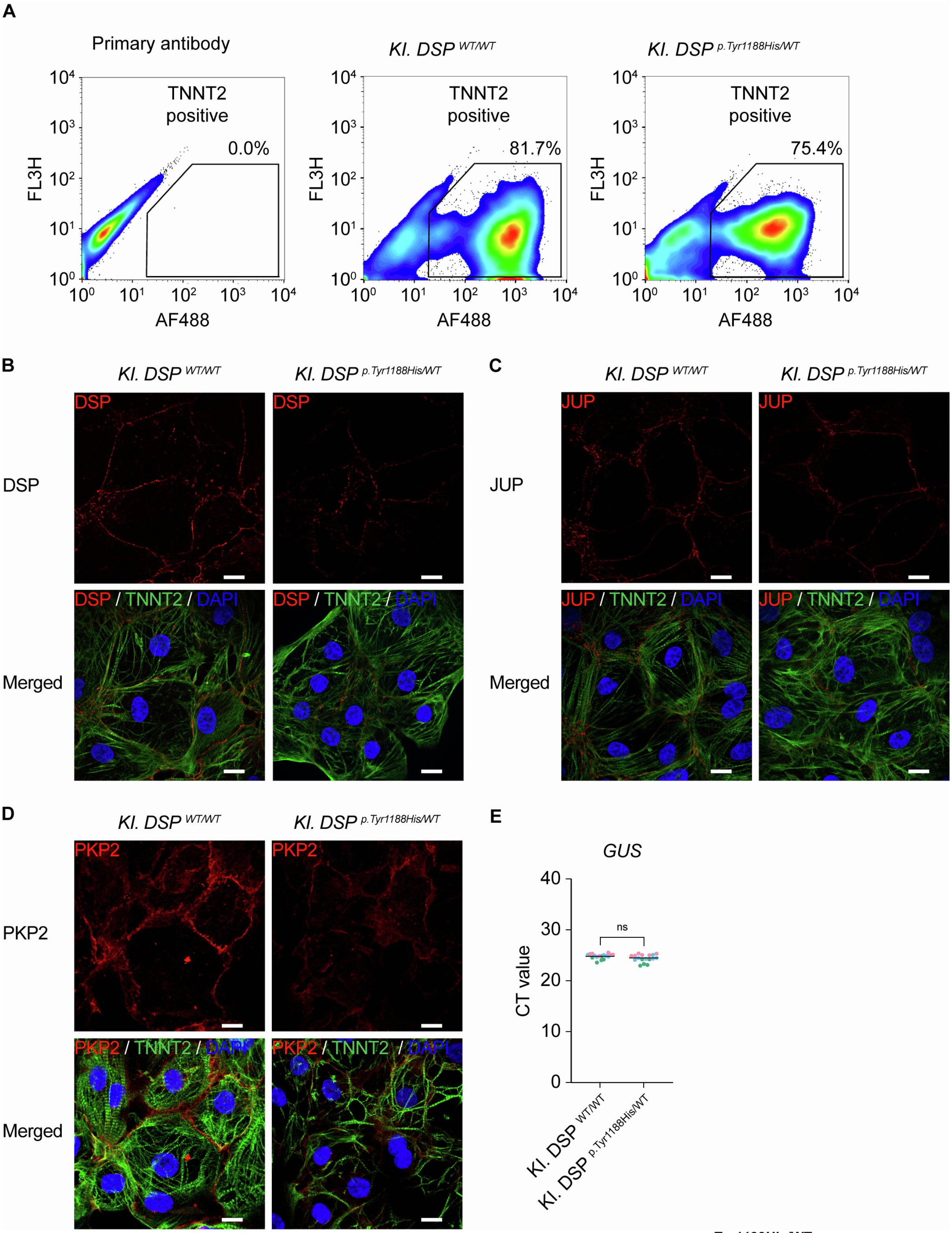

Figure S4. Differentiation and molecular characteristics of *KI. DSP* $^{p.Tyr1188His/WT}$ and isogenic control cardiomyocytes. Related to Figure 4.

(A) Representative FACS plots showing the percentage of cardiomyocytes present after differentiation. Cells were stained with cardiac Troponin T (TNNT2, in green; AF488). (B-D) Representative immunofluorescence images for DSP (B), JUP (C) and PKP2 (D) in one-month-old *KI. DSP* $^{WT/WT}$ and *KI. DSP* $^{p.Tyr1188His/WT}$ cardiomyocytes. DSP, JUP and PKP2 in red; cardiac troponin T (TNNT2) in green; DAPI in blue. Scalebar: 10 µm. (E) Cycle threshold (CT) levels for *GUS* in one-month-old wildtype and mutant hiPSC-derived cardiomyocytes. Data plotted as mean. The dots in (E) represent technical replicates, whereas the color of each dot indicates the experimental origin (3 independent experiments;

5-6 technical replicates). Significance has been assessed by a two-tailed unpaired Student's t-test or two-tailed Mann-Whitney test when data were not normally distributed (ns: not significant).

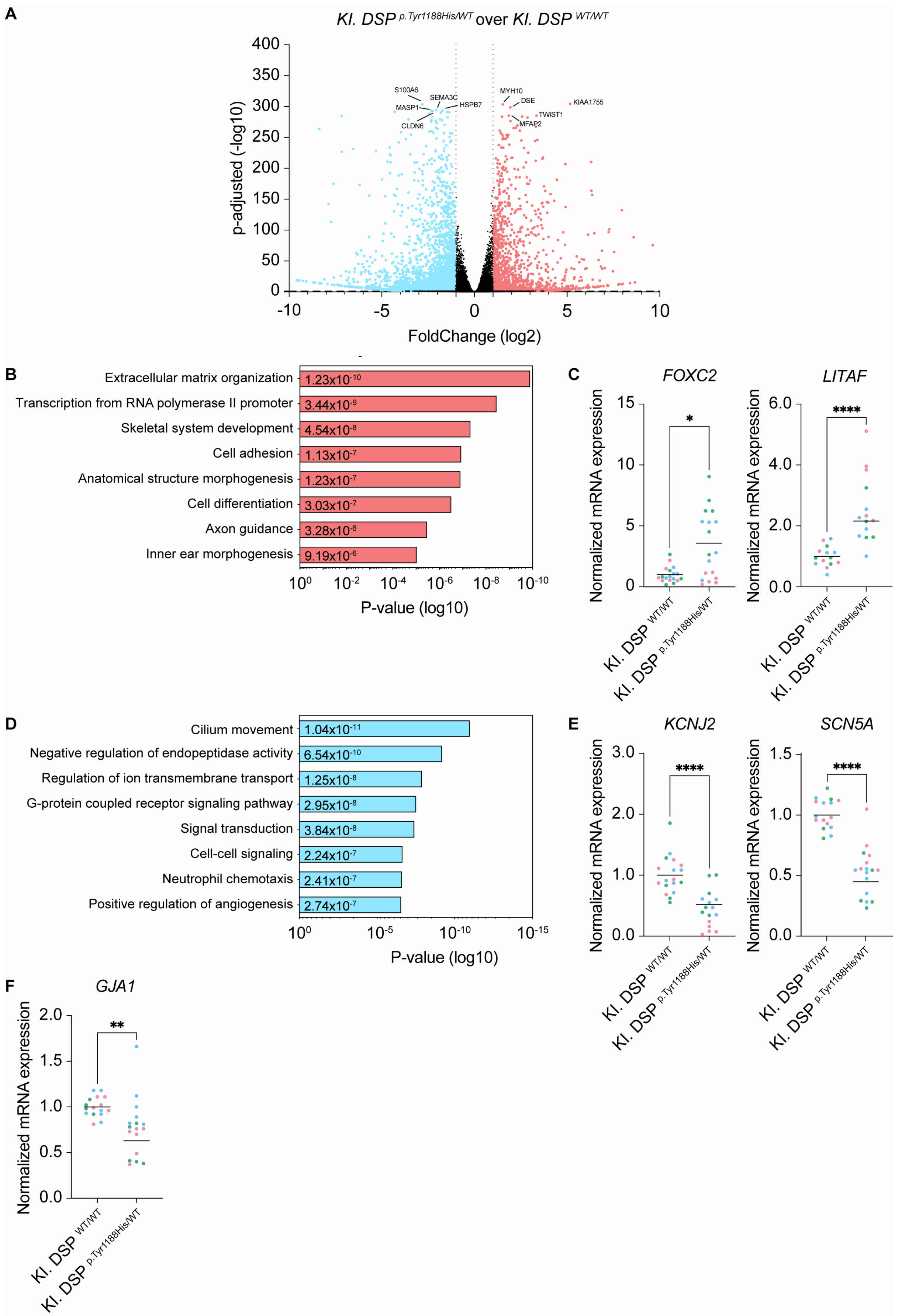

Figure S5. mRNA-sequencing analysis on *KI. DSP* $^{p.Tyr1188His/WT}$ cardiomyocytes reveals ion-handling as enriched term for repressed genes. Related to Figure 4.

(A-E) mRNA-sequencing analyses on one-month-old *KI. DSP* $^{WT/WT}$ and *KI. DSP* $^{p.Tyr1188His/WT}$ cardiomyocytes. (A) Volcano plot showing the up- and downregulated genes (fold change (log2) >1 and <-1; P-adj < 0.05) in mutant cardiomyocytes compared to the isogenic control. The top five up- and downregulated genes are indicated. (B) Gene ontology analysis on the upregulated (fold change (log2) >1) genes. (C) Validation of *FOXC2* and *LITAF*, both belonging to the "Transcription from RNA polymerase II promoter" term. (D) Gene ontology analysis on the downregulated (fold change (log2) <-1) genes. (E) Validation of *KCNJ2* and *SCN5A*, both belonging to the "Regulation of ion transmembrane transport" term. mRNA-sequencing was performed on four replicates obtained from one hiPSC differentiation. (F) Gene expression of *GJA1* in *KI. DSP* $^{WT/WT}$ and *KI. DSP* $^{p.Tyr1188His/WT}$ cardiomyocytes. Data plotted as mean. The dots in (C and E-F) represent technical replicates, whereas the color of each dot indicates the experimental origin (3 independent experiments; 4-6 technical replicates). Significance has been assessed by a two-tailed unpaired Student's t-test or two-tailed Mann-Whitney test when data were not normally distributed (* P-value < 0.05, ** P-value < 0.01, **** P-value < 0.0001).

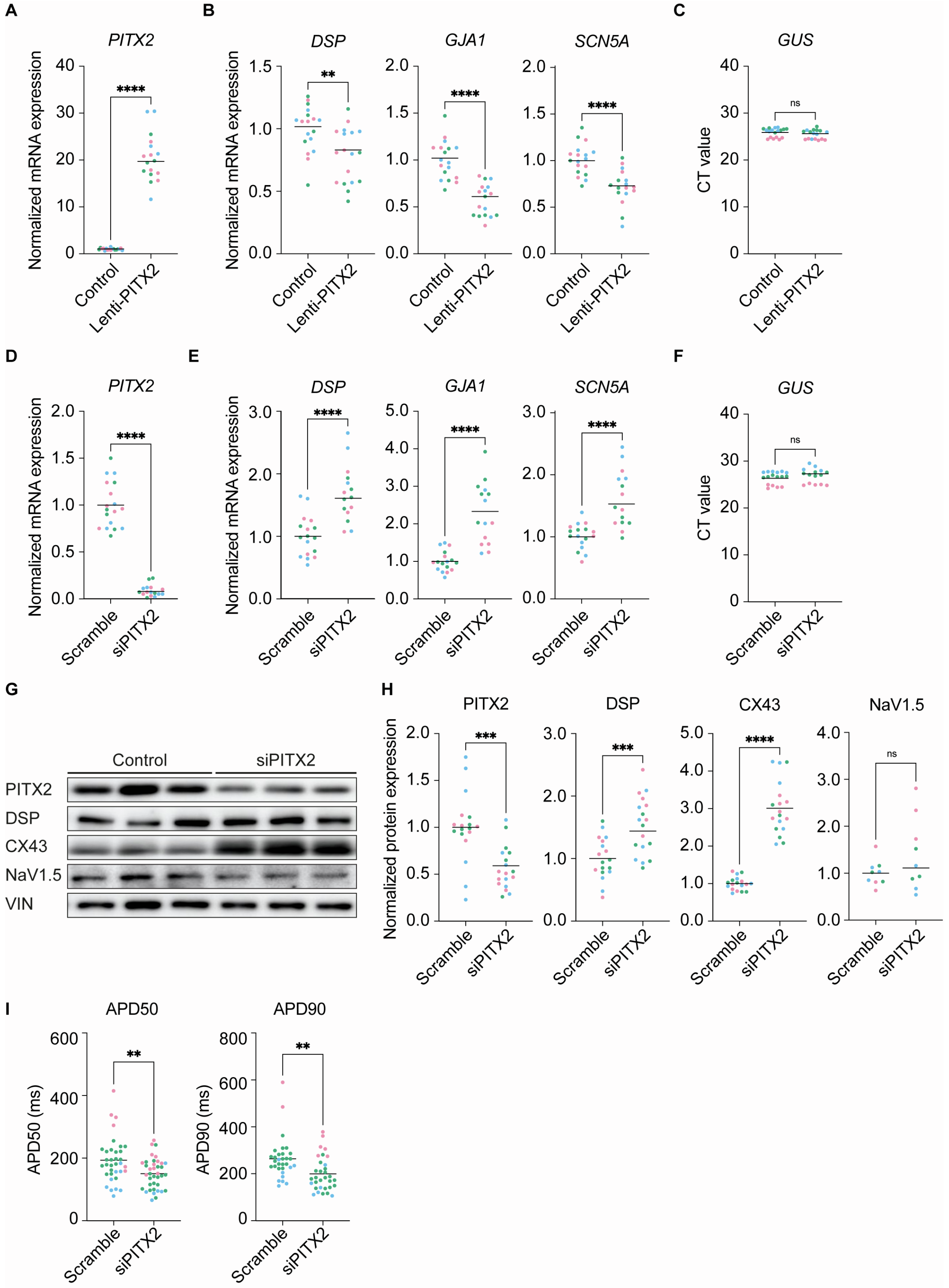

Figure S6. Overexpression and knockdown of paired-like homeodomain 2 in control cardiomyocytes represses and induces expression of cardiac ion- and structural-related genes, respectively. Related to Figure 6.

(A-C) Molecular analyses on one-month-old control hiPSC-derived cardiomyocytes treated with either control lentiviral particles or particles encoding for PITX2 (Lenti-PITX2). Data obtained from three independent experiments. (A) Gene expression levels for *PITX2*. (B) Gene expression levels for *DSP*,

GJA1 and *SCN5A*. (C) Cycle threshold (CT) levels for *GUS* in the corresponding experiments. (D-I) Molecular and functional analyses on one-month-old control hiPSC-derived cardiomyocytes treated with scramble or siRNA against *PITX2*. Data obtained from three independent experiments. (D) Gene expression levels for *PITX2*. (E) Gene expression levels for *DSP*, *GJA1* and *SCN5A* normalized to *GUS*. (F) Cycle threshold (CT) levels for *GUS* in the corresponding differentiations. (G) Representative immunoblots for PITX2, DSP, CX43 and NaV1.5. (H) Quantification of PITX2, DSP, CX43 and NaV1.5 protein levels. Values normalized to VIN. (I) Action potential duration (APD) measured at 50% and 90% of repolarization (control, n= 31 cell clusters; siPITX2, n= 32 cell clusters). Data plotted as mean. The dots in (A-F and H-I) represent technical replicates, whereas the color of each dot indicates the experimental origin (3 independent experiments; 4-20 technical replicates). Significance has been assessed by a two-tailed unpaired Student's t-test or two-tailed Mann-Whitney test when data were not normally distributed (* P-value < 0.05, ** P-value < 0.01, *** P-value < 0.001, **** P-value < 0.0001, ns: not significant).

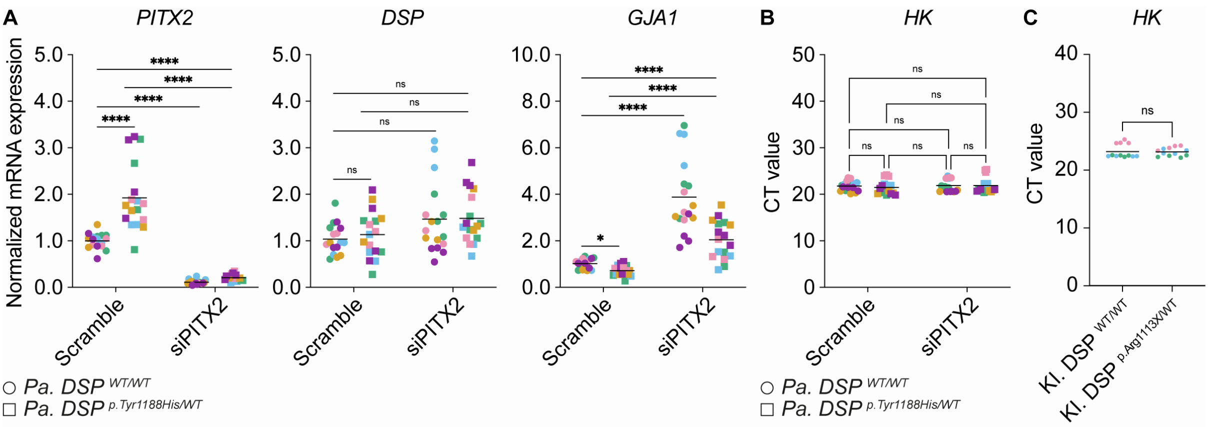

Figure S7. Knockdown of paired-like homeodomain 2 in *Pa. DSP* $^{p.Tyr1188His/WT}$ cardiomyocytes rescues the expression of structural and ion-related genes. Related to Figure 7.

(A) Gene expression levels for *PITX2*, *DSP* and *GJA1*. Values normalized to the average of *GUS*, *HARP* and *RPL32*. (B) Average cycle threshold (CT) levels for *GUS*, *HARP* and *RLP32* in one-month-old *Pa. DSP* $^{p.Tyr1188His/WT}$ cardiomyocytes treated with scramble siRNA or siRNA targeting *PITX2* for 72 h. (C) Average CT levels for *GUS* in one-month-old *KI. DSP* $^{p.Arg1113X/WT}$ cardiomyocytes (Related to Figures 7C-E). Data plotted as mean. The dots in (A-C) represent technical replicates, whereas the color of each dot indicates the experimental origin (3-5 independent experiments; 3-4 technical replicates). For (A-B), significance has been assessed on log-transformed data using an ordinary two-way ANOVA followed by a Tukey's multiple comparisons test (single pooled variance; alpha = 0.05). For (C), significance has been assessed by a two-tailed unpaired Student's t-test (* P-value < 0.05, **** P-value < 0.0001, ns: not significant). HK, housekeeping gene.

Supplemental Tables

Table S1. Clinical features of the patient.

Genetic mutation	*DSP c.3562 T>C/WT*
Protein change	DSP p.Tyr1188His/WT
Age at diagnosis	34 years
Sex	Female
Cardiac abnormalities	- Monomorphic ventricular tachycardia.<br>- Low QRS voltages.<br>- Abnormal repolarization.<br>- Akinesia in the RV apex.<br>- Late enhancement in the lateral wall of the LV.
Implant	1-chamber ICD

ACM, arrhythmogenic cardiomyopathy; DSP, desmoplakin; LV, left ventricle; RV, right ventricle; ICD, implantable cardioverter-defibrillator.

Table S2. Overview of human induced pluripotent stem cell lines used in this study.

Line	Origin	Age at biopsy	Sex	Ethnicity	Reprogramming method	Mutation - Gene	Protein change	Catalog number
Pa. DSP $^{p.Tyr1188His/WT}$	Patient - skin fibroblasts	34 years	F	Caucasian	Sendai virus	DSP c.3562T>C	p.Tyr1188His/WT	N.A.
Pa. DSP $^{WT/WT}$	Patient - skin fibroblasts	34 years	F	Caucasian	Sendai virus	N.A.	WT/WT	N.A.
KI. DSP $^{WT/WT}$	Healthy individual - bone marrow CD34+ cells	31 years	M	Caucasian	Sendai virus	N.A.	WT/WT	ATCC, ACS-1026™
KI. DSP $^{p.Tyr1188His/WT}$	Healthy individual - bone marrow CD34+ cells	31 years	M	Caucasian	N.A.	DSP c.3562T>C	p.Tyr1188His/WT	N.A.
KI. DSP $^{p.Arg1113X/WT}$	Healthy individual - bone marrow CD34+ cells	31 years	M	Caucasian	N.A.	DSP c.3337C>T	p.Arg1113X/WT	N.A.

c., chromosome; DSP, desmoplakin; F, Female; KI, knock-in; M, Male; N.A. not applicable; p., protein; Pa, patient; WT, wildtype.

Table S3. Primers and DNA templates

Name	Sequence (5'->3')	Purpose
DSP_patient-KI_gRNA1_FW	CACCGAGGTTGAGGGTTCTACTGC	CRISPR/Cas9
DSP_patient-KI_gRNA1_RV	AAACGCAGTAGAACCCTCAACCTC	CRISPR/Cas9
DSP_patient-KI_gRNA2_FW	CACCGTACGAGATTGAAAGGTTGA	CRISPR/Cas9
DSP_patient-KI_gRNA2_RV	AAACTCAACCTTTCAATCTCGTAC	CRISPR/Cas9
DSP_patient-KI_gRNA3_FW	CACCGTAAGAAACCACTATAATG	CRISPR/Cas9
DSP_patient-KI_gRNA3_RV	AAACCATTATAGTGGTTTCTTAC	CRISPR/Cas9
DSP_patient-KI_gRNA4_FW	CACCGTACTCATCTCCTCATTATAG	CRISPR/Cas9
DSP_patient-KI_gRNA4_RV	AAACCTATAATGAGGAGATGAGTAC	CRISPR/Cas9
DSP-universal_FW	ATATGAGCAGCTGGTGCAAG	Genotyping
DSP-universal_RV	CTTGGCCTCCTCCTGAAAC	Genotyping
DSP_patient_ssODN	TGTGAAAAGGAGAACCTTGGTTGG CAGAAATTAGAGTCTGAGAAAGCCA TCAAGGAGAAGGAGTACGAGATTG AAAGGTTGAGGGTTCTACTACAAGA AGAAGGTACCCGGAAGAGAGAATA TGAAAATGAGCTGGCAAAGGTAAG AAACCACTATAATGAGGAGATGAGT AATTTAAGGAACAAGTATGAAACAG AGAT	DNA template – CRISPR/Cas9
DSP_KI_ssODN1	AGAAGGAGTACGAGATTGAAAGGT TGAGGGTTCTACTGCAAGAAGAAG GTACCCGGAAGAGAGAACATGAAA ATGAGCTGGCAAAGGTAAGAAACC ACTATAATGAGGAGATGAGTAATTT AAGGAA	DNA template – CRISPR/Cas9
DSP_KI_ssODN2	AGAAGGAGTACGAGATTGAAAGGT TGAGGGTTCTACTGCAAGAAGAAG GTACCCGGAAGAGAGAATATGAAA ATGAGCTGGCAAAGGTAAGAAACC ACTATAATGAGGAGATGAGTAATTT AAGGAA	DNA template – CRISPR/Cas9
Off-target 1 - DSP_patient-KI_FW	AGCTTCCCTCCCTTGACTCT	Amplification of potential off-target site
Off-target 1 - DSP_patient-KI_RV	GAAGCTACAGGGCAAGATGG	Amplification of potential off-target site
Off-target 2 - DSP_patient-KI_FW	GCTCAAAGAACAAGCTGCTGA	Amplification of potential off-target site
Off-target 2 - DSP_patient-KI_RV	GGATGATCTCATATTAAGATTTTCC AA	Amplification of potential off-target site
Off-target 3 - DSP_patient-KI_FW	GCCTCTTGGGTTCAAACAAT	Amplification of potential off-target site
Off-target 3 - DSP_patient-KI_RV	ACCCTGCAGGAGAAAAACCT	Amplification of potential off-target site

DSP; desmoplakin; FW, forward; gRNA, single guide RNA; KI, knock-in; RV, reverse; ssODN, single-stranded oligodeoxynucleotide.

Table S4. Antibodies and dilutions.

Target	Host species	Dilution / Application	Product ID	Company
Plakophilin (PKP2)	Goat	1:1000 - IB	Ab189323	Abcam
Plakophilin-2 (PKP2)	Mouse	1:100 – IF	651167	Santa Cruz
Plakoglobin (PKG)	Mouse	1:1000 - IB	sc398183	Santa Cruz
		1:500 - IF		
Desmoplakin (DSP)	Rabbit	1:1000 - IB	ab71690	Abcam
Desmoplakin (DSP)	Mouse	1:50 - IF	65146	Progen
Desmocollin (DSC)	Mouse	1:250 - IB	32-6200	ThermoFisher Scientific
Desmoglein (DSG)	Mouse	1:100 - IB	61002	Progen
Paired-like homeodomain 2 (PITX2)	Rabbit	1:1000 - IB	PA-1020-100	Capra Sciences
Connexin 43 (CX43)	Rabbit	1:2000 - IB	C6219	Sigma-Aldrich
Sodium channel protein type 5 subunit alpha (NaV1.5)	Rabbit	1:500 - IB	ASC-005	Alomone
Vinculin (VIN)	Mouse	1:1000 - IB	sc25336	Santa Cruz
Cardiac Troponin T (TNNT2)	Rabbit	1:400 - IF	ab45932	Abcam
Alpha-actinin-2 (ACTN2)	Mouse	1:400 - IF	A7811	Sigma-Aldrich
FLAG	Mouse	1:2500 - IP	F3165	Sigma-Aldrich
GFP	Mouse	1:2000 - IP	11814460001	Roche

IB, immunoblotting; IF, immunofluorescence; IP, immunoprecipitation.

Table S5. Quantitative PCR primers.

Name	Forward - sequence (5'->3')	Reverse - sequence (5'->3')
Desmocollin (*DSC*)	TGGTAGAGTTAACCTGAAAGAGTG	TGGTTCTCAGTGTTGGAAAGT
Desmoglein (*DSG*)	GGAACACAGCAGCTACACTT	ACCATCCCTTCAAGCACTTTAT
Desmoplakin (*DSP*)	GCACCAGCAGGATGTACTATT	TCAATTCAGGCTGCACGAT
Forkhead Box C2 (*FOXC2*)	CCTCCTGGTATCTCAACCACA	GAGGGTCGAGTTCTCAATCCC
Frizzled Class Receptor 2 (*FZD2*)	GTGCCATCCTATCTCAGCTACA	CTGCATGTCTACCAAGTACGTG
Gap junction alpha-1 protein (*GJA1*)	TTAAGGATCGGGTTAAGGGAAAG	TGTACCCAGGAGGAGACATAG
Glucuronidase Beta (*GUS*)	CCACCTAGAATCTGCTGGCTAC	GTGCCCGTAGTCGTGATACCAA
Ribosomal Protein Lateral Stalk Subunit P0 (*HARP*)	CACCATTGAAATCCTGAGTGATGT	TGACCAGCCCAAAGGAGAAG
Plakoglobin (*JUP*)	ACCAGGAGAGCAAGCTGAT	CTCCACAATGGCAGGCTTATT
Potassium Inwardly Rectifying Channel Subfamily J Member 2 (*KCNJ2*)	ACCGCTACAGCATCGTCTCT	TCCACACACGTGGTGAAGAT
Lipopolysaccharide Induced TNF Factor (*LITAF*)	ATGTCGGTTCCAGGACCTTAC	TACGAAGGAGGATTCATGCCC
Paired-like homeodomain 2 (*PITX2*)	ACTTTCCAGAGGAACCGCTAC	GTTGCGCTCCCTCTTTCTC
Plakophilin-2 (*PKP2*)	TGCTAAAGGCTGGCACAA	TAATCGCTGTGCGTGTAGTG
Ribosomal Protein L32 (*RPL32*)	CAACGTCAAGGAGCTGGAAG	TGGGGTTGGTGACTCTGATG
Sodium Voltage-Gated Channel Alpha Subunit 5 (*SCN5A*)	TCTCTATGGCAATCCACCCCA	GAGGACATACAAGGCGTTGGT
Secreted Frizzled Related Protein 4 (*SFRP4*)	CGAGCTGCCTGTCTATGACC	ATCCACTTAACATCCTCCGGG

Supplemental Experimental Procedures

Co-immunoprecipitation

HEK293T cells were grown in Dulbecc's Modified Eagl's Medium (ThermoFisher Scientific, 11965084) supplemented with 10% fetal bovine serum (Merck) and 100 U/mL penicillin-streptomycin (ThermoFisher Scientific, 15140122) on a 145 cm^2 dish until 60-70% confluency. Cells were then transfected with 25 μg of each plasmid using 1 mg/mL polyethylenimine (PEI; Polysciences, 23966). Medium was refreshed 18 h after transfection. Cells were harvested after 48 h using 7 mL cold PBS with 1 mM EDTA, followed by centrifugation at 300 RCF for 5 minutes. Supernatant was aspirated and cell pellet was resuspended in 1 mL NP-40 lysis buffer (150 mM NaCl, 50 mM Tris-HCl pH 7.6, 0.2% NP-40, 1 mM EDTA, 10% Glycerol) and incubated for 30 minutes at 4°C. After incubation, 25 μL of lysis product was collected for the input. Dynabeads™ Protein G (Invitrogen, 10004D) were coated with 2 μg/reaction of Monoclonal anti-FLAG® M2 (Sigma-Aldrich, F3165) according to the manufacturer's instructions. All washing steps were performed using PBS pH 7.4 with 0.02% Tween™ 20 (Sigma-Aldrich, P1379-1L) and the beads were coated at room temperature for 20 minutes. Coated beads were subsequently added to 1 mL of lysis product and incubated overnight at 4°C while rotating. Beads were processed according to the manufacturer's instructions. For the elution step, beads were placed on a magnetic stand followed by removal of all supernatant. Next, each reaction was incubated for 30 minutes at 4°Cwith 75 μL TBS (150 mM NaCl, 50 mM Tris-HCL pH 7.6 in water) supplemented with 25 μg/reaction 3x FLAG peptide (Sigma-Aldrich, F4799). Supernatant was transferred to a new 1.5 mL tube followed by addition of 4x loading dye (250 mM Tris, 8% SDS, 40% Glycerol, 20% 2-mercapto-ethanol). Samples were incubated for 3 minutes at 95°Cand stored at -20°C. Immunoprecipitation reactions (25 μL) together with 2 μL of input were then analyzed on a 7% SDS-PAGE gel using the antibodies listed in Table S4.

Cell culture

hiPSCs were cultured on Geltrex™ lactose dehydrogenase elevating virus-free, human embryonic stem cell-Qualified, Reduced Growth Factor Basement Membrane Matrix-coated wells (Gibco, A1413302). Cells were refreshed with Essential 8™ Medium (Gibco, A1517001) on a daily basis. hiPSCs were passaged at 80-100% confluency. Briefly, medium was aspirated and TrypLE Express Enzyme (Gibco, 12605010) was added for 5 minutes at 37°C. After incubation, 4 mL of Essential 8™ Medium, supplemented with 2 μM thiazovivin (Sigma-Aldrich, 420220), was added to the dissociated cells and transferred to a 15 mL Falcon tube. Cells were centrifuged for 3 minutes at 300 RCF. Subsequently, cells were seeded at a density of 15,000 cells/cm^2 in Essential 8™ Medium, supplemented with 2 μM thiazovivin. Medium was refreshed the next day with plain Essential 8™.

Genome targeting with CRISPR/Cas9

gRNA sequences were selected with the CCTop- CRISPR/Cas9 target online predictor tool (Labuhn et al., 2018; Stemmer et al., 2015). No changes were made to the default parameters. Four gRNAs were selected (Table S3). gRNAs were cloned into the pSpCas9(BB)-2A-GFP vector (Addgene, #48138). A T7-endonuclease assay was performed to estimate the cutting efficiency of each gRNA. Briefly, HEK293T cells were grown in a 6-wells well until a confluency of 70%. Cells were then transfected with 1 μg of each gRNA using 6 μg PEI. After 48h, genomic DNA was isolated using the DNeasy Blood and Tissue kit (Qiagen, 69506) according to the instructions of the manufacturer. Modified DNA regions were amplified by polymerase chain reaction (PCR) using GoTaq® Green Master Mix (Promega, M712) and the primers shown in Table S3. NEBuffer 2.1 (BioLabs®$_{Inc}$ New England, B7202S) was added to each reaction, followed by denaturation at 95ºC for 5 minutes and ramping down the temperature to 25ºC (-5ºC/minutes). Next, T7 endonuclease I (BioLabs®$_{Inc}$ New England, M0302S) enzyme was added to each reaction and incubated for 30 minutes at 37ºC. Digested products were analyzed on a 2% weight/volume agarose gel. The most effective gRNA was selected based on the cutting efficiency and its proximity to the mutation-of-interest; for which gRNAs closer to this site are predicted to be better. Next, the mutation-of-interest was introduced into hiPSCs. Briefly, hiPSCs were pretreated with Essential 8™ Medium supplemented with 2 μM thiazovivin for at least 1 hour. hiPSCs were then dissociated with TrypLE Express Enzyme for 5 minutes at 37°C. Dissociated cells were collected in 4 mL of Essential 8™ Medium, supplemented with 2 μM thiazovivin and centrifuged for 3 minutes at 300 RCF. Supernatant was removed and the cell pellet was resuspended in 1 mL of Essential 8™ Medium, supplemented with 2 μM thiazovivin. Cell number was assessed with the Countess II FL (Invitrogen, AMQAF1000). Two million cells were collected in a 15 mL Falcon tube and centrifuged for 3 minutes at 300 RCF. Supernatant was aspirated and the cell pellet was resuspended in 100 μL prewarmed (37°C)

nucleofection mix (Lonza Human Stem Cell Nucleofector™ Kit 1, VPH-5012) supplemented with 5 μg pSpCas9(BB)-2A-GFP vector containing the gRNA sequence and 1 μg of asymmetrically designed single-stranded oligodeoxynucleotides containing the mutation-of-interest (IDT ultramer; Table S3)(Richardson et al., 2016). Cells were transferred to an electroporation cuvette and placed in the Nucleofector™ device (Lonza), and were then nucleofected using the A-023 program for human stem cells. 500 μL of warm Essential 8™ Medium supplemented with 2 μM thiazovivin was added to the cuvette. Cells were then plated into two Geltrex coated wells in a 6-well plate. After 24 h, cells were dissociated as described above and single-cell sorted (BD biosciences, FACSJazz™) based on GFP expression in 96-well plates coated with irradiated mouse embryonic fibroblasts (ThermoFisher Scientific, A34961). 96-well plates were centrifuged for 1 minute at 200RCF and then incubated at 37°C. After 10-14 days, clones were dissociated for 5-10 minutes using Versene Solution (Gibco, 15040066) and passaged into two wells of a 96-well plate. Genomic DNA was isolated 1-2 days later using a standard ethanol-based precipitation method. The genomic region-of-interest of each clone was amplified using GoTaq® Green Master Mix and the primers listed in Table S3. Amplified product was then sent for Sanger sequencing (Macrogen, Amsterdam, The Netherlands).

Karyotyping

Genomic DNA integrity of modified hiPSCs was assessed by karyotyping. Cells grown in the exponential phase were incubated with 0.05 µg/mL colcemid (Gibco, 152120-012) for 90 minutes at 37ºC. Medium was collected and the cells were rinsed once with dPBS (Gibco, 14190094). Cells were then dissociated for 5 minutes using TrypLE Express Enzyme and centrifuged for 5 minutes at 300 RCF. Supernatant was removed and the cell pellet was resuspended in 1 mL of prewarmed 75 mM KCl while shaking constantly. After 10 min, 50 μL of freshly prepared methanol:acetic acid (3:1 ratio) solution was added to the cells in a dropwise manner. Cells were centrifuged for 5 minutes at 300 RCF. Supernatant was removed and the cell pellet was resuspended in 1 mL of methanol:acetic acid (3:1 ratio) solution while shaking constantly. The mixture was left at room temperature for 20 min, after which the cells were centrifuged for 5 minutes at 300 RCF. The last two steps were repeated twice. After the last centrifugation step, the cell pellet was resuspended in 0.5 mL methanol:acetic acid (3:1 ratio) solution. Drops of the cell suspension were then dropped from approximately 50 cm high on a glass microscope slide. The slides were dried in an incubator. Finally, the slides were mounted with ProLong™ Gold Antifade Mountant with DAPI (ThermoFisher Scientific, P36935). Chromosome spreads were imaged using the Leica TCS SPE Confocal Microscope.

Karyo-sequencing

Approximately 1000 hiPSCs were collected as a pellet. Five μL of 2 μg Proteinase K (NEB, P8107S) in 1x CutSmart Buffer (NEB, B6004S) was added for 2 h at 55°C followed by 10 minutes at 80°C. DNA was digested using 10 μL of 10 U NLAIII (NEB, R0125S) in 1x CutSmart Buffer for 2 h at 37°C followed by 20 minutes at 80°C. DNA fragments were ligated to adapters by adding 20 μL of 800 U T4 DNA ligase (NEB, M0202S), 1 mM ATP (ThermoFisher Scientific, R0441) and 50 nM adapter in 1x T4 DNA ligase buffer and incubating at 16°C overnight. The library preparation, sequencing and analysis was performed as described previously (Bolhaqueiro et al., 2019).

Off-targets

Top three exonic or intronic off-target sites, as predicted by the CCTop tool, were PCR amplified using GoTaq® G2 Hot Start Master Mix and the primers lister in Table S3.

Cardiomyocyte differentiation

hiPSCs were maintained as described above and grown until 80-90% confluency (Day 0). Medium was aspirated and cells were washed once with dPBS. Next, cells were fed with RPMI-1640-Medium-GlutaMAX™Supplement-HEPES (Gibco, 72400-021) supplemented with 0.5 mg/mL human recombinant albumin (Sigma-Aldrich, A9731), 0.2 mg/mL L-Ascorbic Acid 2-Phosphate (Sigma-Aldrich, A8960), and 4 μM CHIR99021 (Sigma-Aldrich, 361559). After 48 h (Day 2), medium was aspirated and cells were washed once with RPMI-1640-Medium-GlutaMAX™Supplement-HEPES, followed by addition of RPMI-1640-Medium-GlutaMAX™Supplement-HEPES supplemented with 0.5 mg/mL human recombinant albumin, 0.2 mg/mL L-Ascorbic Acid 2-Phosphate, and 5 μM IWP2 (Sigma-Aldrich, 681671). On day 4 and day 6, cells were refreshed with RPMI-1640-Medium-GlutaMAX™Supplement-HEPES supplemented with 0.5 mg/mL human recombinant albumin and 0.2 mg/mL L-Ascorbic Acid 2-Phosphate. From day 8 onwards, cells were refreshed every 3-4 days with RPMI-1640-Medium-GlutaMAX™-Supplement-HEPES supplemented with B-27™Supplement (50x)-serum free (Gibco, 17504001). hiPSC-derived cardiomyocytes were subsequently dissociated with TrypLE™ Select

Enzyme (10x) without phenol red (Gibco, A1217703) for a maximum of 45 minutes at 37°C. Cardiomyocytes were seeded at a density of 100,000 cells/cm^2 in Geltrex-coated wells for downstream molecular applications.

Cardiomyocyte purity
hiPSC-derived cardiomyocytes were dissociated with TrypLE™ Select Enzyme (10x) without phenol red. One million cardiomyocytes were centrifuged for 5 minutes at 300 RCF. Medium was removed and cells were washed once with dPBS. Cells were then resuspended in 1 mL of ice-cold 70% ethanol. After fixation, cells were centrifuged for 4 minutes at 300 RCF, followed by aspiration of the fixative. Cells were resuspended in PBS (pH7.2-7.4) supplemented with 5% fetal bovine serum, 1% BSA, and 0.5% Triton X-100 (blocking buffer) and permeabilized for 10 minutes on ice. Permeabilized cells were centrifuged for 4 minutes at 300 RCF and the supernatant was aspirated. The cell pellet was resuspended in 100 μL blocking buffer supplemented with anti-Cardiac Troponin T antibody and incubated for 1 hour at 4°C. After 1 hour, 500 μL of blocking solution was added and the cells were centrifuged for 4 minutes at 300 RCF. Supernatant was aspirated and cells were resuspended once more in 500 μL blocking buffer, followed by centrifugation for 4 minutes at 300 RCF and resuspension of the cells in 100 μL blocking buffer supplemented with Alexa 488-anti-rabbit antibody. After 30 minutes incubation at room temperature, 500 μL of blocking buffer was added to the cells, followed by a centrifugation step of 4 minutes at 300 RCF. The supernatant was removed and cells were resuspended again with 500 μL of blocking buffer, followed by another centrifugation for 4 minutes at 300 RCF. Lastly, the supernatant was aspirated and the cells were resuspended in 1 mL of dPBS and analyzed by FACS (BD biosciences, FACSCalibur™). Antibodies and the dilutions used can be found in Table S4.

Electrophysiology
hiPSC-derived cardiomyocytes were seeded per 100,000 cells on Geltrex-coated glass coverslips, to allow cluster formation. Coverslips were incubated for 15 minutes at 37°Cwith 1:1000 voltage sensitive dye FluoVolt and 1:1000 Powerload (Thermo Fischer Scientific, F10488) in RPMI-1640-Medium-GlutaMAX™-Supplement-HEPES supplemented with B-27™Supplement (50x)-serum free. During measurements, cells were immersed in a solution containing in mM: NaCl (130), KCl (4), CaCl2 (1.8), MgCl2 (1.2), NaHCO3 (18), HEPES (10) and glucose (10), pH 7.4. A custom-build microscope (Cairn Research, Kent, UK) with a 10x objective was used for the recordings. Excitation of the dye was done by a blue light, using a 482/35 excitation filter (Semrock FF01-482/35-25), and captured, using a 514 long-pass emission filter (Semrock LP02-514RU-25), with a high-speed camera (Andor Zyla 5.5.CL3, Oxford Instruments). A custom MATLAB script was used for analysis of the action potentials (Boer). Action potential durations were corrected for the beating rate using an adjustment of the Fredericia formula used to correct the QT interval for heart rate: APDcorrected=APD/($\sqrt[3]{(60/BPM)}$)(Blinova et al., 2017).

Immunoblotting
hiPSC-derived cardiomyocytes were dissociated with TrypLE™ Select Enzyme (10x) and collected in a 1.5 mL Eppendorf tube. Cells were centrifuged for 5 minutes at 300 RCF. Supernatant was removed and cells were resuspended in 1 mL dPBS followed by centrifugation at 300 RCF for 5 min. Cells were lysed in RIPA buffer (50 mM Tris-pH7.5, 150 mM NaCl, 0.1% SDS, 0.5% sodium deoxycholate, 1% Triton X-100) supplemented with one tablet of cOmplete™ EDTA-free Protease Inhibitor Cocktail (Roche, 11836170001) and one tablet of PhosSTOP™ (Roche, 4906837001) per 10 mL of RIPA buffer. Immunoblotting was performed using 10-15 μg of protein extract. For detection, the corresponding secondary antibody coupled to horseradish peroxidase was used in combination with the Clarity™ Western ECL Substrate kit (Bio Rad, 1705061). Immunoblots were imaged with an Amersham Imager 680RGB device (GE Healthcare, 29270772) and quantified with ImageJ. Antibodies and the dilutions used can be found in Table S4.

Immunocytochemistry
hiPSC-derived cardiomyocytes were seeded on Geltrex-coated glass coverslips with a diameter of 12 mm. Seven days later, cells were washed once with dPBS and then fixed with 2% paraformaldehyde for 30 minutes at room temperature. Cardiomyocytes were washed 3x with PBS and permeabilized with 0,1% Triton X-100 in PBS for 8 minutes at room temperature. Permeabilized cells were blocked with 4% goat serum in PBS for 1 hour at room temperature. Cells were then incubated with primary antibody diluted in 4% goat serum solution and incubated overnight at 4°C. Next, cells were washed three times with PBS and incubated with the corresponding secondary antibody dissolved in 4% goat serum solution for 1 hour at room temperature. Nuclei were stained with DAPI for 5 minutes at room

temperature. Cells were mounted with Mowiol (24% (w/v) Glycerol (Baker, 7044), 9.6% (w/v) Mowiol 4-88 (Calbiochem, 475904), 0.1 M Tris-HCl pH 8.5) and imaged using a Leica TCS SPE Confocal Microscope. Antibodies and the dilutions used can be found in Table S4.

Quantitative PCR

Total RNA was isolated from hiPSC-derived cardiomyocytes using the RNeasy Mini Kit (Qiagen, 74104) following the protocol supplied by the manufacturer. Total RNA was reverse transcribed using the iScript™ cDNA Synthesis Kit (Bio Rad, 1708891). Quantitative PCR (qPCR) reactions were performed on a Bio Rad CFX96 Real-Time PCR Detection System using the iQ SYBR Green Supermix kit (Bio Rad, 170-8885). Primers used for amplification can be found in Table S5. The ΔΔCt-method was used to analyze the data. Control groups were set at 1. Gene expression data presented in Figure 7 and Figure S8 were corrected for session-to-session variation prior to statistical analyses using a previously published method (Ruijter et al., 2006).

mRNA-sequencing

mRNA-sequencing was performed on one-month-old hiPSC-derived *Pa. DSP* $^{WT/WT}$, *Pa. DSP* $^{p.Tyr1188His/WT}$, *KI. DSP* $^{WT/WT}$ and *KI. DSP* $^{p.Tyr1188His/WT}$ cardiomyocytes (four technical replicates per line). Total RNA isolation was performed as described above. RNA libraries were prepared with the TruSeq Stranded mRNA polyA kit (Illumina) according to the manufacturer's protocol. Strand-specific single-end 75 bp reads were generated on an Illumina NextSeq 500 system. On average, we obtained 33.000.000 reads per sample, which were used for subsequent analysis. Reads were checked for their quality using FastQC, and aligned against the human genome (assembly GRCh37) using STAR (STAR_2.4.2a). Counting aligned sequencing reads per gene was done with the HTSeq-count package using the union mode. Finally, differential expression was calculated using DESeq2 v1.2 with pooled dispersion estimates (Love et al., 2014). Genes were considered significantly different when FoldChange > 2.0 or < -2.0 and p adjusted < 0.05.

Enrichment analysis

Differentially expressed genes, obtained with the DESeq2 pipeline, were further analyzed for functional enrichment using the STRINGv11.5 database (Szklarczyk et al., 2021). No changes were made to the default settings. Homo sapiens served as background. Only the molecular function (GO-MF), biological process (GO-BP), cellular component (GO-CC), and KEGG data sources were considered for enrichment analysis.

Knockdown experiments

Three-week-old hiPSC-derived cardiomyocytes were dissociated using TrypLE™ Select Enzyme (10x), no phenol red and seeded at a cell density of 100,000 cells/cm^2 in Geltrex-coated wells with RPMI-1640-Medium-GlutaMAX™Supplement-HEPES supplemented with B-27™ Supplement (50x)-serum free and 2 µM thiazovivin. After 24 h, medium was refreshed with RPMI-1640-Medium-GlutaMAX™Supplement-HEPES supplemented with B-27™ Supplement (50x)-serum free. Seven days after reseeding, cells were transfected with either 10 nM scramble or siRNA oligo duplexes targeting *PITX2* (OriGene, SR321325) utilizing Lipofectamine™ RNAiMAX (ThermoFisher, 13778150) according to the manufacturers' instructions. RNA and protein samples were collected as described above after 48 h and 72 h, respectively.

Overexpression experiments

Two-week-old hiPSC-derived cardiomyocytes were dissociated using TrypLE™ Select Enzyme (10x), no phenol red and seeded at a cell density of 100,000 cells/cm^2 in Geltrex-coated wells with RPMI-1640-Medium-GlutaMAX™Supplement-HEPES supplemented with B-27™ Supplement (50x)-serum free and 2 µM thiazovivin. After 24 h, medium was refreshed with RPMI-1640-Medium-GlutaMAX™Supplement-HEPES supplemented with B-27™ Supplement (50x)-serum free. Seven days after reseeding, cells were infected with either PITX2C (ENST00000644743.1) cloned into the pLVX-IRES-Hygro (Clontech, 632185) vector backbone or pLVX-EGFP vector. After seven days, RNA and protein samples were collected (as described above). The viral particles used were harvested 48 h after transfection of 7x10^6 HEK293X cells with 7 µg pLVX-IRES-Hygro or pLVX-EGFP together with 8 µg psPAX2 (Addgene, #12260) and 3 µg pMD2G (Addgene, #12259).

Human material

The study fulfilled the Dutch criteria of the code of proper use of human tissue. Written informed consent was obtained for the generation and use of the patient-derived hiPSCs.

Supplemental References

Blinova, K., Stohlman, J., Vicente, J., Chan, D., Johannesen, L., Hortigon-Vinagre, M.P., Zamora, V., Smith, G., Crumb, W.J., Pang, L., et al. (2017). Comprehensive translational assessment of human-induced pluripotent stem cell derived cardiomyocytes for evaluating drug-induced arrhythmias. Toxicol. Sci. *155*, 234–247.

Boer, T. de Peaks, software for analysing cardiac APs and calcium transients.

Bolhaqueiro, A.C.F., Ponsioen, B., Bakker, B., Klaasen, S.J., Kucukkose, E., van Jaarsveld, R.H., Vivié, J., Verlaan-Klink, I., Hami, N., Spierings, D.C.J., et al. (2019). Ongoing chromosomal instability and karyotype evolution in human colorectal cancer organoids. Nat. Genet. *51*, 824–834.

Labuhn, M., Adams, F.F., Ng, M., Knoess, S., Schambach, A., Charpentier, E.M., Schwarzer, A., Mateo, J.L., Klusmann, J.H., and Heckl, D. (2018). Refined sgRNA efficacy prediction improves largeand small-scale CRISPR-Cas9 applications. Nucleic Acids Res. *46*, 1375–1385.

Love, M.I., Huber, W., and Anders, S. (2014). Moderated estimation of fold change and dispersion for RNA-seq data with DESeq2. Genome Biol. *15*, 550.

Richardson, C.D., Ray, G.J., DeWitt, M.A., Curie, G.L., and Corn, J.E. (2016). Enhancing homology-directed genome editing by catalytically active and inactive CRISPR-Cas9 using asymmetric donor DNA. Nat. Biotechnol. *34*, 339–344.

Stemmer, M., Thumberger, T., del Sol Keyer, M., Wittbrodt, J., and Mateo, J.L. (2015). CCTop: An Intuitive, Flexible and Reliable CRISPR/Cas9 Target Prediction Tool. PLoS One *10*, e0124633.

Szklarczyk, D., Gable, A.L., Nastou, K.C., Lyon, D., Kirsch, R., Pyysalo, S., Doncheva, N.T., Legeay, M., Fang, T., Bork, P., et al. (2021). The STRING database in 2021: Customizable protein-protein networks, and functional characterization of user-uploaded gene/measurement sets. Nucleic Acids Res. *49*, D605–D612.
